# Implementation Strategies for Knowledge Products in Primary Health Care: Systematic Review of Systematic Reviews

**DOI:** 10.2196/38419

**Published:** 2022-07-11

**Authors:** Claude Bernard Uwizeye, Hervé Tchala Vignon Zomahoun, André Bussières, Aliki Thomas, Dahlia Kairy, José Massougbodji, Nathalie Rheault, Sébastien Tchoubi, Leonel Philibert, Serigne Abib Gaye, Lobna Khadraoui, Ali Ben Charif, Ella Diendéré, Léa Langlois, Michèle Dugas, France Légaré

**Affiliations:** 1 Learning Health System Component of the Québec Strategy for Patient-Oriented Research (SPOR) - Support for People and Patient-Oriented and Trials (SUPPORT) Unit Québec, QC Canada; 2 VITAM Research Center on Sustainable Health Laval University Québec, QC Canada; 3 Centre Intégré Universitaire de Santé et de Services Sociaux de la Capitale-Nationale (CIUSSS-CN) Québec, QC Canada; 4 Department of Social and Preventive Medicine Laval University Québec, QC Canada; 5 School of Physical and Occupational Therapy Faculty of Medicine and Health Sciences McGill University Montreal, QC Canada; 6 Centre de Recherche Interdisciplinaire en Réadaptation du Montréal métropolitain (CRIR) Montreal, QC Canada; 7 Réseau Provincial de recherche en Adaptation-Réadaptation (REPAR) Montreal, QC Canada; 8 Institut Universitaire sur la Réadaptation en Déficience Physique de Montréal (IURDPM) Montreal, QC Canada; 9 Institut National de Santé Publique du Québec Québec, QC Canada; 10 Faculty of Nursing Laval University Québec, QC Canada; 11 Tier 1 Canada Research Chair in Shared Decision Making and Knowledge Translation Laval University Québec, QC Canada; 12 CubecXpert Québec, QC Canada; 13 Department of Family Medicine and Emergency Medicine Laval University Québec, QC Canada

**Keywords:** knowledge translation, knowledge product, implementation strategies, review, health care professionals, primary care

## Abstract

**Background:**

The underuse or overuse of knowledge products leads to waste in health care, and primary care is no exception.

**Objective:**

This study aimed to characterize which knowledge products are frequently implemented, the implementation strategies used in primary care, and the implementation outcomes that are measured.

**Methods:**

We performed a systematic review (SR) of SRs using the Cochrane systematic approach to include eligible SRs. The inclusion criteria were any primary care contexts, health care professionals and patients, any Effective Practice and Organization of Care implementation strategies of specified knowledge products, any comparators, and any implementation outcomes based on the Proctor framework. We searched the MEDLINE, EMBASE, CINAHL, Ovid PsycINFO, Web of Science, and Cochrane Library databases from their inception to October 2019 without any restrictions. We searched the references of the included SRs. Pairs of reviewers independently performed selection, data extraction, and methodological quality assessment by using *A Measurement Tool to Assess Systematic Reviews 2*. Data extraction was informed by the Effective Practice and Organization of Care taxonomy for implementation strategies and the Proctor framework for implementation outcomes. We performed a descriptive analysis and summarized the results by using a narrative synthesis.

**Results:**

Of the 11,101 records identified, 81 (0.73%) SRs were included. Of these 81, a total of 47 (58%) SRs involved health care professionals alone. Moreover, 15 SRs had a high or moderate methodological quality. Most of them addressed 1 type of knowledge product (56/81, 69%), common clinical practice guidelines (26/56, 46%) or management, and behavioral or pharmacological health interventions (24/56, 43%). Mixed strategies were used for implementation (67/81, 83%), predominantly education-based (meetings in 60/81, 74%; materials distribution in 59/81, 73%; and academic detailing in 45/81, 56%), reminder (53/81, 36%), and audit and feedback (40/81, 49%) strategies. Education meetings (*P*=.13) and academic detailing (*P*=.11) seemed to be used more when the population was composed of health care professionals alone. Improvements in the adoption of knowledge products were the most commonly measured outcome (72/81, 89%). The evidence level was reported in 12% (10/81) of SRs on 62 outcomes (including 48 improvements in adoption), of which 16 (26%) outcomes were of moderate or high level.

**Conclusions:**

Clinical practice guidelines and management and behavioral or pharmacological health interventions are the most commonly implemented knowledge products and are implemented through the mixed use of educational, reminder, and audit and feedback strategies. There is a need for a strong methodology for the SR of randomized controlled trials to explore their effectiveness and the entire cascade of implementation outcomes.

## Introduction

### Background

The effective implementation of knowledge products is essential for improving and sustaining the well-being of populations and reducing waste in health care. In 2019, health care spending represented 17.7% of the US gross domestic products [1] and 11.5% of that for Canada [2]. However, the underuse of effective knowledge products that would be beneficial to the population, combined with the misuse or overuse of knowledge products that offer no added value or even provide more harm than benefits to populations, contribute to this lack of impact and waste [3,4]. Knowledge products include a wide range of health interventions or policies, programs, practices, or processes of technological, pharmacological, behavioral, or managerial nature and guidelines [5,6].

Given this gap between the production of knowledge products and their application in clinical practices and health policies, a growing emphasis has been placed on knowledge translation (KT) [7,8] and implementation strategies [8-10]. Implementation strategies can be understood as an actively planned and deliberately initiated set of processes, methods, techniques, activities, and resources, with the intention of translating a given knowledge product into practice within a particular setting and context [5,11-13].

In recent years, given the many constraints on resources (human and financial) faced by most, if not all, health care systems, which have recently been made even worse by the COVID-19 pandemic [14], there has been a growing urgency in regard to synthetizing what is known about effective implementation strategies [9,15-24]. Despite these efforts, gaps in KT remain in relation to overviews of variable methodological and reporting qualities [25], which sometimes lead to conflicting conclusions and make it challenging for health care stakeholders to decide which strategies are effective for the implementation of a given knowledge product. This concern has not been explicitly addressed in the existing literature.

Therefore, we planned a 3-phase project, with the ultimate goal to identify, for each category of knowledge product, the most effective implementation strategies for their uptake into health care professionals’ clinical practice. The first phase was to critically analyze the existing literature overviews to determine their strengths and weaknesses. This allowed us to highlight many methodological challenges such as the definition of eligibility criteria and literature search, the way in which data were synthesized, the methodological quality assessment of the literature reviews included, and the assessment of the evidence level. These points informed the realization of the present systematic review (SR) of SRs, which is the second phase of our project.

### Objective

We sought to characterize which knowledge products are frequently implemented, the implementation strategies used in primary care, and the implementation outcomes measured.

## Methods

### Project Design and Registration

To optimize the identification of effective implementation strategies in the area of primary care, we conducted a 3-phase project using SR methodologies. In phase 1 (completed review), we conducted a critical analysis of the methodological strengths and weaknesses of the existing overviews. In phase 2 (current overview), we conducted an SR of SRs to characterize the most frequently implemented knowledge products, implementation strategies used, implementation outcomes measured, and reported levels of evidence in individuals or stakeholders participating in the provision of health care (referred to as health care professionals) or in health care professionals and end users (patients and clients) in the context of primary health care. In the included SRs, primary studies may either be of more robust experimental designs (randomized controlled trials [RCTs]) or less robust designs. Therefore, the effectiveness of key knowledge products and key implementation strategies was measured in a separate phase 3 (future review) using an SR of RCTs.

The protocol of the project was registered on the Open Science Framework platform on February 7, 2020 [26] and then published [27]. The review was conducted following the Cochrane methodology [28] and is reported in accordance with the PRISMA (Preferred Reporting Items for Systematic Reviews and Meta-Analyses) guidelines [29].

### Eligibility Criteria

We used the population, intervention, comparison and outcomes format [30] to delineate our inclusion criteria.

#### Population and Clinical Context

We included any person involved in health care provision, that is, health care professionals or caregivers and end users (patients). By caregivers, we mean the parents, guardians, friends of patients, community health workers, or any other nonclinician who provides health care. The empirical studies in the included reviews could concern either health care professionals or caregivers alone, or health care professionals or caregivers and patients. They were excluded from cases in which only the patients were concerned. We did not place restrictions on age, gender, or health conditions. Reviews had to cover the primary care setting [31], as it is a major level of health service use. Rather than targeting the physical location of activities, we were interested in primary health care services, such as health promotion and prevention, diagnosis, and treatment of illness and injury. By primary health care services, we refer to family physicians, nurse practitioners, and pharmacists who ensure the direct provision of health care services to clients and coordinate to ensure the continuity of care to upper levels [31].

#### Intervention

We focused on implementation strategies that were predetermined in our protocol [27] and based on the Effective Practice and Organization of Care (EPOC) [8] to include the following implementation strategies: audit, feedback, audit and feedback, clinical incident reporting, monitoring the performance of the delivery of health care, communities of practice, continuous quality improvement, educational games, educational materials, educational meetings, educational outreach visits or academic detailing, clinical practice guidelines, interprofessional education, local consensus processes, local opinion leaders, managerial supervision, patient-mediated interventions, public release of performance data, reminders, routine patient-reported outcome measures, and tailored interventions. A review may have included primary studies that use exclusively 1 type of implementation strategy (mono-faceted) or exclusively more than 1 type (multifaceted). Within the same review, some primary studies may have used exclusively one implementation strategy, whereas others may have used exclusively more than one implementation strategy (mixed). We excluded interventions that were used to develop the knowledge product and the scaling up and sustainability of interventions (studies that were housed under a separate project). Knowledge products are tools used to share knowledge with users [32,33]. They include tools such as clinical practice guidelines, decision support tools, policy briefs or decision-making tools, one pagers, and health interventions (technological, pharmacological, behavioral, or management). In the clinical practice guidelines category, we included clinical practice guidelines, disease management protocols, clinical recommendations, and clinical procedures. For health interventions, we included knowledge products for which the implementation aimed to change professional behavior or attitude (behavioral), professional competencies or processes or quality of care (management), prescribing or testing (pharmacological), and the use of technologies (technological). In the shared decision-making and support tools category, we included clinical decision support systems and tools aimed at improving clinical decision-making. In a given SR, 1 type of knowledge product (single) or more than 1 type (multiple) may have been implemented. A review was included if the knowledge product and implementation strategies were specified.

#### Comparators

We considered either usual practice (no predetermined implementation strategies as defined previously) or any of the predetermined implementation strategies defined earlier.

#### Outcomes

Our interest was focused on implementation outcomes, including acceptability, adoption, appropriateness, feasibility, adherence or fidelity, implementation costs, and penetration or reach of a knowledge product, as defined in the taxonomies by Proctor et al [34] and Lewis et al [35]. Detailed definitions are provided in Multimedia Appendix 1. Several of these outcomes may have been studied in the same SR.

#### Design of Included Reviews

We included both Cochrane and non-Cochrane SRs (with or without meta-analyses) and mixed method reviews that used a comprehensive and reproducible approach and met our inclusion criteria. The reviews may have included one or more types of experimental or observational primary study designs. We excluded reviews of reviews, non-SRs, original research, protocols, comments, editorials, conference abstracts, working groups and colloquium reports, experts’ opinions, and pilot studies.

### Information Sources and Search Strategy

We searched MEDLINE (Ovid), EMBASE, CINAHL (EBSCO), Ovid PsycINFO (Ovid), Web of Science (Web of Science), and Cochrane Library (Cochrane Library) databases from their inception to October 18, 2019, without restrictions on language or geographic settings. We searched the bibliographies of the included reviews to identify additional relevant ones.

We followed an extensive literature search process to identify SRs of interventions that implement health knowledge products. In March 2017, an information specialist (NR) designed the search strategy for each database. The initial search strategy developed in MEDLINE was reviewed and approved by some of the team members before its translation into other bibliographic databases by the information specialist. During the selection process, gaps were identified in the search strategy. The search strategy was modified and rerun in October 2019. We used the following main concepts: KT, strategies, reviews, health professionals, and primary care. Multimedia Appendix 2 details the search strategy for each of the aforementioned databases. The records found were exported to the EndNote software (Clarivate), and duplicates were removed.

### Study Selection

**Figure 1:**
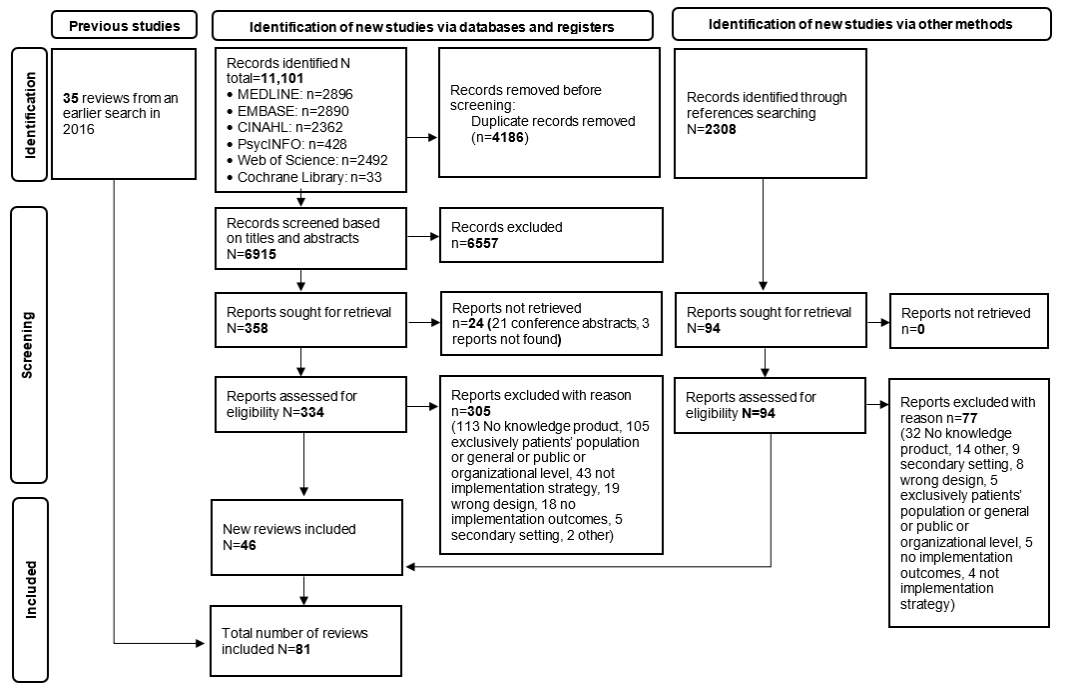
PRISMA (Preferred Reporting Items for Systematic Reviews and Meta-Analyses) flowchart of study screening and selection.

We used Microsoft Excel developed for our review to perform the study selection in 3 steps. First, our reviewers performed pilot selection and held discussions regarding any discordance to ensure a common understanding of the eligibility criteria before subsequent steps were taken. Second, pairs of reviewers independently screened the titles and abstracts. Records coded as *included* or *unclear* were eligible for a full report review against the inclusion criteria by pairs of reviewers. Third, full reports were coded on one side as *included* or *unclear* and as *excluded* on the other side. At each step, consensus discussions were held to resolve disagreements. A senior reviewer validated the final list of included SRs. We did not need to contact any of the review authors. A flow diagram, according to the PRISMA guidelines [29], was produced to summarize the process of study selection (Figure 1).

### Data Extraction

We used the piloted Microsoft Excel format developed for our review to extract the data. To develop the format, we used the taxonomies of the EPOC [8] for the categories of implementation strategies and complemented the information by specifying whether the implementation strategies were mono-faceted, multifaceted, or mixed. For the outcome definitions, we used the Proctor et al [34] and Lewis et al [35] evaluation frameworks. These frameworks integrate more dimensions not found in other frameworks, such as acceptability, appropriateness, feasibility, and implementation costs. They also provide outcome synonyms found in the literature, thus facilitating recategorization when needed. For each outcome, we specified whether the measurement was objective and the measurement tools used, if reported. Evidence-based interventions are practices in which health professionals use available evidence-based information to make decisions for individual patients or community health [6,36]. It operates by appraising evidence and formulating recommendations or guidelines [6,37,38] and by integrating evidence and community preferences for policy and practice changes at the public health level (health interventions) [6,38]. We were unable to find a formal taxonomy of knowledge products; therefore, we used the literature [5,6,32,33] to categorize whether they were clinical practice guidelines, health interventions, or shared decision-making and support tools. In cases where they were health interventions, we specified their technological, pharmacological, behavioral, or management nature. Furthermore, we extracted information regarding whether the type of implemented knowledge product was single (eg, clinical practice guidelines alone) or multiple (eg, clinical practice guidelines and health interventions). The population was defined as health care professionals only or health care professionals and patients and their number and characteristics of age and gender were extracted where available.

To give context to our review, the following additional information was also extracted: general characteristics of the included review (such as year of publication, number and names of databases searched, search date ranges considered, any language restriction, method of synthesizing data, medical area of concern, settings, designs, and number of primary studies), whether the authors of the included reviews completed methodological quality assessment (tools used and overall result), whether they completed publication bias assessment (tool used and whether any treatment was done), and whether they completed the assessment of quality of evidence (tool used and level of evidence by each reported outcome).

Pairs of reviewers piloted the tool on at least 2 reviews and independently carried out extractions and validations by comparing the extracted information. Discussions for consensus were held in case of discrepancies.

### Assessment of Methodological Quality

The methodological quality of the included reviews was assessed using *A Measurement Tool to Assess Systematic Reviews* (AMSTAR; AMSTAR 2) [39]. In contrast to the first version, this updated version allows the assessment of SRs that include RCTs, nonrandomized studies of health interventions, or both [39]. We conducted a pilot phase and held discussions on discordance. Where necessary, the pilot phase was extended until a common understanding of the assessment criteria was achieved. Pairs of assessors independently scored each of these 16 items. An overall rating was also provided, which indicated high (no or one noncritical flaw), moderate (more than one noncritical flaw), low (one critical flaw with or without noncritical flaws), or critically low (more than one critical flaw with or without noncritical flaws) ratings [39]. Critical flaws included protocol not registered before the beginning of the review (standard 2), lack of adequacy and comprehensiveness of the search strategy (standard 4), no provision of the justification for excluding individual reviews (standard 7), the use of an unsatisfactory technique to assess the risk of bias from individual included reviews (standard 9), the inappropriateness of meta-analytical methods (standard 11), no consideration of the risk of bias when interpreting the results of the review (standard 13), and lack of suitability for the assessment of the presence and the likely impact of publication bias (standard 15) [39]. Reviewers compared their results and reached a consensus in cases of disagreement by discussion or by the arbitration of a third reviewer.

### Data Synthesis

For the second phase, reanalysis by meta-analysis was not performed [27]. Using SAS software (SAS Institute Inc) and taking the included review as the unit of analysis, we performed a descriptive analysis that aimed to summarize the characteristics of the implemented knowledge products, implementation strategies used, outcomes measured, and levels of evidence reported. We summarized the data as numbers and percentages for categorical variables and as means and SDs or medians and IQRs for continuous variables. Counts were performed overall and then stratified according to methodological quality scores (high, moderate, low, and very low). We grouped *Technological Health Interventions and Decision Support Tools* as implemented clinical decision support tools were electronic or computerized decision support systems.

For reviews in which the level of evidence of outcomes was measured and reported, we summarized what was reported as the level of evidence for the reported implementation outcome by the implementation strategy used and by the specific implemented single knowledge product. We used the number of outcomes as the unit of analysis.

## Results

### Search and Selection Process

Our database search identified 11,101 records, of which 6915 (62.29%) titles and abstracts were screened after removing duplicates. Among these 6915, a total of 428 (6.19%) full reports were screened for eligibility, after which 81 (18.9%) admissible SRs remained [40-120] (Figure 1). The reasons for the exclusion of each examined full report are provided in Multimedia Appendix 3.

### General Characteristics of the Included Reviews and Participants

Table 1 shows the key general characteristics of the 81 included SRs. They were published between 1989 and 2019, with a mean of 8 (SD 5.8) years since the last bibliographic search in 2019. The authors of SRs searched an average of 6 databases, whereas more than half of the reviews (41/81, 51% of the SRs) restricted their search to SRs in the English language [40-43,45,47,49,50,52,55-57,64,66,69-73,79,83-85,88-91,93,96-100, 104,105,107,109,114,118-120]. Individual SRs included a mean number of 29 primary studies. Of 81 SRs, with the exception of 8 (10%) SRs [43,46,54,68,70,78,89,107], all remaining SRs (n=73, 90%) included primary studies designed as RCTs. Non-RCTs were included in 42% (34/81) of SRs [40,42,45,48,50,51,53,55,64,67,69-74,76,79-81,83,84,88, 90-93,96,97,101,108,109,114,115]. The settings covered were either primary or secondary health care (56/81, 69%) or primary health care alone in 31% (25/81) of SRs [43,45,46,48,50,52,57,71,78,81,83,86,88,90,91,95-98, 102,104-106,111,114]. A total of 58% (47/81) of SRs involved health care professionals alone [40,42,44,45,49,50,53-55, 60,62,63,65-68,70,71,73-76,79,81-84,86-88,93-95,99,101,102, 107-110,112-115,117,119,120], whereas the remaining 42% (34/81) involved health care professionals and patients at the same time. In 83% (67/81) of SRs, primary studies were critically appraised for methodological quality [40-42,44, 45,47-53,55-68,70-87,89,91-96,99,100,102,104,106-115,117,119], and the narrative approach was used to synthesize information in 80% (65/81) of SRs [40-43,45,46,48-56, 60,62-64,66-70,72-76,78-80,83,85-101,103, 105-109,112-120]. A detailed table of the key general characteristics for each included review is available in Multimedia Appendix 4 [40-120].

**Table 1:** General characteristics of included reviews overall and by methodological quality scores (reviews: N=81).

Characteristics			Overall		Methodological quality						
					High		Moderate		Low		Critically low
Analyzed systematic reviews, n (%)			81 (100)		9 (11)		6 (7)		17 (21)		49 (61)
**Age of reviews (years), n (%)**			81 (100)		9 (11)		6 (7)		17 (21)		49 (61)
	Value, mean (SD)	8.0 (5.8)		4.8 (3.1)		9.1 (5.9)		6.0 (3.3)		9.1 (6.6)	
	Value, median (IQR)	6.8 (3.8-10.8)		3.8 (2.8-6.8)		7.3 (5.8-8.8)		5.8 (3.8-7.8)		7.8 (3.8-12.8)	
**Databases searched in included reviews, n (%)**			81 (100)		9 (11)		6 (7)		17 (21)		49 (61)
	Value, mean (SD)	6.3 (3.8)		10.3 (5.6)		9.7 (4.1)		6.6 (3.3)		5.0 (2.8)	
	Value, median (IQR)	5.0 (3.0)		7.0 (10.0)		9.5 (3.0)		6.0 (4.0)		5.0 (4.0)	
**Search language restriction in included reviews, n (%)**			81 (100)		9 (11)		6 (7)		17 (21)		49 (61)
	Yes	48 (59)		1 (11)		2 (33)		12 (70)		33 (67)	
	No	19 (24)		5 (56)		4 (67)		4 (24)		6 (12)	
	Not reported	14 (17)		3 (33)		0 (0)		1 (6)		10 (21)	
**Language restrictions, n (%)**			48 (100)		1 (2)		2 (4)		12 (25)		33 (69)
	English only	41 (85)		1 (100)		2 (100)		11 (92)		27 (82)	
	English and other languages	7 (15)		0 (0)		0 (0)		1 (8)		6 (18)	
**Primary studies included in included reviews, n (%)**			80^a^ (100)		9 (11)		6 (8)		17 (21)		48 (60)
	Value, mean (SD)	29.2 (34.7)		20.3 (12.2)		16.8 (13.1)		27.2 (20.3)		33.1 (42.3)	
	Value, median (IQR)	19.5 (10.5-34.5)		19.0 (12.0-26.0)		14.0 (8.0-19.0)		22.0 (11.0-38.0)		19.0 (11.0-38.0)	
**How many reviews included the following designs of primary studies^b^, n (%)**			81 (100)		9 (11)		6 (7)		17 (21)		49 (61)
	Randomized controlled trials	73 (90)		9 (100)		6 (100)		15 (88)		43 (88)	
	Nonrandomized controlled trials	34 (42)		2 (22)		4 (67)		9 (53)		19 (39)	
	Interrupted time series	20 (25)		4 (44)		3 (50)		5 (29)		8 (16)	
	Cohorts	11 (17)		1 (11)		0 (0)		2 (12)		8 (16)	
	Before-after	26 (32)		0 (0)		2 (33)		5 (29)		19 (39)	
	Other	22 (27)		1 (11)		0 (0)		5 (29)		16 (33)	
**Settings (health domains), n (%)**			81 (100)		9 (11)		6 (7)		17 (21)		49 (61)
	Primary and secondary health care	56 (69)		9 (100)		6 (100)		13 (76)		28 (57)	
	Primary health care only	25 (31)		0 (0)		0 (0)		4 (24)		21 (43)	
**Method of analysis for included reviews,** **n (%)**			81 (100)		9 (11)		6 (7)		17 (21)		49 (61)
	Narrative	65 (80)		5 (56)		5 (83)		15 (88)		40 (82)	
	Mixed synthesis	10 (12)		3 (33)		1 (17)		1 (6)		5 (10)	
	Meta-analysis	6 (8)		1 (11)		0 (0)		1 (6)		4 (8)	
**Were primary studies critically appraised for methodological quality, n (%)**			81 (100)		9 (11)		6 (7)		17 (21)		49 (61)
	Yes	67 (83)		9 (100)		6 (100)		17 (100)		35 (71)	
	No	10 (12)		0 (0)		0 (0)		0 (0)		10 (21)	
	Not reported	4 (5)		0 (0)		0 (0)		0 (0)		4 (8)	
**Population of reviews, n (%)**			81 (100)		9 (11)		6 (7)		17 (21)		49 (61)
	Health care professionals only	47 (58)		6 (67)		5 (83)		8 (47)		28 (57)	
	Health care professionals and patients	34 (42)		3 (33)		1 (17)		9 (53)		21 (43)	

^a^One study without a number of included studies.

^b^Categories are not mutually exclusive.

### Implementation Strategies, Knowledge Products, and Outcomes

#### Implementation Strategies

In 6% (5/81) of SRs [57,98,102,110,117], all primary studies in SR used only 1 type of implementation strategy (mono-faceted). In 11% (9/81) of SRs [40,58,60,66,86,89,97,111,112], all primary studies in SR exclusively used more than 1 type of implementation strategy (multifaceted). In the remaining 83% (67/81) of SRs, some primary studies used 1 type of implementation strategy, whereas others used more than 1 type of implementation strategy (mixed; Table 2). Educational strategies were the most frequently used, mainly educational meetings in 74% (60/81) of SRs [40-42,44-46,49,50,52,53,55,58-66,68-71,73-79,81-89,91,92,94-100, 103,104,107,109,111-114,118-120], educational materials distribution in 73% (59/81) of SRs [40-42, 44-46,48-55,58,60-66,69-71,73-77,79-81,83-89,91,92,94-97,100, 103,105,108,110,112-116,118-120] and educational outreach in 56% (45/81) of SRs [40-42,44,46,48,50-52, 54,55,60,63-65,69,71,73-77,79,81-83,85-88,92,94,95,97,99, 100,103-105,108,112-115,119] (Table 2). Other frequent strategies used were reminders in 65% (53/81) of SRs [41,42,44,47,48,50,51,53-58,60-64,66,67,69-71,73,76,77,79,81, 83,85,86,88,89,91,92,94-97,99-102,105,106,111-116,118,119], audit and feedback for 49% (40/81) of SRs [40,42,44,50,51,53-55,60,61,63-65,70,73,74,76,77,79,81-83,85,87, 91-94,97,99,100,104,106,111,113-116,118,119] and the use of local opinion leaders for 43% (35/81) of SRs [40,41,44-46,49,51,52,54,60,63,66,69,73-77,79,81,85,86,89,91,92, 94,96,97,99,105,108,109,112,118,119] (Table 2).

**Table 2:** Characteristics of included reviews related to knowledge products, implementation strategies, and outcomes by methodological quality (reviews: N=81).

Characteristics		Overall, n (%)	Methodological quality, n (%)			
			High	Moderate	Low	Critically low
Analyzed systematic reviews		81 (100)	9 (11)	6 (7)	17 (21)	49 (61)
**Type of knowledge products implemented**		81 (100)	9 (11)	6 (7)	17 (21)	49 (61)
	Single	56 (69)	4 (44)	4 (67)	13 (76)	35 (71)
	Multiple	25 (31)	5 (56)	2 (33)	4 (24)	14 (29)
**Categories of single knowledge products**		56 (100)	4 (7)	4 (7)	13 (23)	35 (63)
	Clinical practice guidelines	26 (46)	0 (0)	1 (25)	9 (70)	16 (46)
	Management, behavioral, and pharmacological health interventions	24 (43)	3 (75)	3 (75)	2 (15)	16 (46)
	Health technology interventions and decision support tools	6 (11)	1 (25)	0 (0)	2 (15)	3 (8)
**Types of implementation strategies**		81 (100)	9 (11)	6 (7)	17 (21)	49 (61)
	Mixed	67 (83)	7 (78)	5 (83)	15 (88)	40 (82)
	Multifaceted only	9 (11)	2 (22)	1 (17)	2 (12)	4 (8)
	Mono-faceted only	5 (6)	0 (0)	0 (0)	0 (0)	5 (10)
**Implementation strategies categories^a^**		81 (100)	9 (11)	6 (7)	17 (21)	49 (61)
	Educational meetings	60 (74)	8 (89)	5 (83)	12 (71)	35 (71)
	Educational materials	59 (73)	8 (88.9)	6 (100)	10 (59)	35 (71)
	Reminders	53 (64)	8 (89)	2 (33)	12 (71)	31 (63)
	Educational outreach visits or academic detailing	45 (56)	5 (56)	5 (83)	9 (53)	26 (53)
	Audit and feedback	40 (49)	6 (67)	4 (67)	10 (59)	20 (41)
	Local opinion leaders	35 (43)	3 (33)	4 (67)	7 (41)	21 (43)
	Feedback	32 (40)	5 (56)	1 (17)	5 (29)	21 (43)
	Clinical practice guidelines	23 (28)	3 (33)	1 (17)	4 (24)	15 (31)
	Local consensus processes	18 (21)	2 (22)	2 (33)	2 (12)	12 (25)
	Tailored interventions	15 (17)	3 (33)	0 (0)	2 (12)	10 (20)
	Audit	13 (16)	1 (11)	0 (0)	4 (24)	8 (16)
	Patient-mediated interventions	11 (14)	1 (11)	0 (0)	1 (6)	9 (18)
	Interprofessional education	9 (11)	1 (11)	0 (0)	3 (18)	5 (10)
	Continuous quality improvement	9 (10)	2 (22)	0 (0)	2 (12)	5 (10)
	Monitoring the performance of the delivery of health care	6 (7)	1 (11)	0 (0)	1 (6)	4 (8)
	Managerial supervision	6 (7)	0 (0)	0 (0)	0 (0)	6 (12)
	Educational games	5 (6)	0 (0)	0 (0)	1 (6)	4 (8)
	Communities of practice	2 (3)	0 (0)	0 (0)	0 (0)	2 (4)
	Clinical incident reporting	1 (1)	0 (0)	0 (0)	0 (0)	1 (2)
	Routine patient-reported outcome measures	1 (1)	1 (11)	0 (0)	0 (0)	0 (0)
	Public release of performance data	0 (0)	0 (0)	0 (0)	0 (0)	0 (0)
**How outcomes were measured^a^**		81 (100)	9 (11)	6 (7)	17 (21)	49 (61)
	Not reported	47 (58)	4 (44)	4 (67)	7 (41)	32 (65)
	Objective	29 (36)	6 (67)	4 (67)	8 (47)	11 (23)
	Both	18 (22)	1 (11)	1 (17)	4 (24)	12 (25)
	Self-administered	11 (14)	0 (0)	2 (33)	1 (6)	8 (16)
**Implementation outcomes^a^**		81 (100)	9 (11)	6 (7)	17 (21)	49 (61)
	Adoption	72 (89)	8 (89)	6 (100)	14 (82)	44 (90)
	Other	28 (35)	4 (44)	4 (67)	4 (24)	16 (33)
	Implementation costs	16 (20)	1 (11)	2 (33)	3 (18)	10 (20)
	Acceptability	15 (19)	2 (22)	2 (33)	3 (18)	8 (16)
	Fidelity	9 (11)	1 (11)	0 (0)	1 (6)	7 (14)
	Penetration	6 (7)	0 (0)	0 (0)	0 (0)	6 (12)
	Appropriateness	5 (6)	0 (0)	0 (0)	1 (5.9)	4 (8)
	Sustainability	4 (5)	0 (0)	0 (0)	0 (0)	4 (8)
	Feasibility	3 (4)	0 (0)	0 (0)	0 (0)	3 (6)
**Other outcomes^a^**		28	4 (14)	4 (14)	4 (14)	16 (58)
	Knowledge	19 (68)	1 (25)	4 (100)	3 (75)	11 (69)
	Attitudes	10 (36)	2 (50)	2 (50)	1 (25)	5 (31)
	Performance in a test situation	9 (32)	1 (25)	1 (25)	1 (25)	6 (38)
	Satisfaction	8 (29)	2 (50)	2 (50)	1 (25)	3 (19)

^a^Categories are not mutually exclusive.

#### Knowledge Products

Of the 81 SRs, 56 (69%) focused on the implementation of single-type knowledge products, including 26 (46%) for clinical practice guidelines [41,48,51,54-56,69,70,72-78,81,83,91, 100,101,108,110-112,114,115], 24 (43%) for health interventions of management and behavioral or pharmacological nature [46,52,57-60,67,71,82,84,86,88,92,94-96,98,102-104, 106,107,113,116], and 6 (11%) for health technology interventions and decision support tools [47,53,65,68,80,93]. In the remaining 31% (25/81) of SRs, multiple knowledge products were the subjects of implementation (Table 2).

#### Knowledge Products by Implementation Strategy

The strategies used varied based on the knowledge product being implemented; clinical practice guidelines were commonly implemented using educational material distribution (21/26, 81%) [41,48,51,54,55,69,70,73-77,81,83,91,100,108,110,112, 114,115], reminders (20/26, 77%) [41,48,51,54-56,69, 70,73,76,77,81,83,91,100,101,111,112,114,115], and academic detailing (18/26, 69%) [41,48,51,54,55,69,73-77,81,83, 100,108,112,114,115]. The simultaneous use of these 3 strategies to implement clinical practice guidelines was reported in 60% (15/26) of SRs [41,48,51,54,55,69,73,76, 77,81,83,100,112,114,115].

For health interventions of management and behavioral or pharmacological nature, the implementation strategies included education meetings (19/24, 79%) [46,52,58-60,71, 82,84,86,88,92,94-96,98,103,104,107,113], educational material distribution (15/24, 63%) [46,52,58,60,71,84,86,88, 92,94-96,103,113,116], and reminders (15/24, 63%) [57,58, 60,67,71,86,88,92,94-96,102,106,113,116]. Their simultaneous use was reported in 42% (10/24) of SRs [58,60, 71,86,88,92,94-96,113].

The same pattern was observed for health technology interventions and decision support tools implemented using education meetings (3/6, 50%) [53,65,68], educational material distribution (3/6, 50%) [53,65,80], and audit and feedback (3/6, 50%) [53,65,93]. The simultaneous use of these strategies has been reported in 17% (1/6) of SRs [53].

We compared the proportions of implementation strategies used when the population was health care professionals alone and when the population was health care professionals and patients. Education meetings and academic detailing seem to be used when the population is composed of health care professionals alone, without any statistical significance (Table 3).

**Table 3:** Implementation strategies used by type of population (reviews: N=81).

Implementation strategies^a^	Health care professionals alone (n=47), n (%)	Health care professionals and patients (n=34), n (%)	*P* value
Education meetings	38 (81)	22 (65)	.13
Educational materials	37 (78)	22 (65)	.21
Academic detailing	30 (64)	15 (44)	.11
Reminders	30 (64)	23 (68)	.81
Audit and feedback	26 (55)	14 (41)	.26
Local opinion leaders	21 (45)	14 (41)	.82

^a^Categories are not mutually exclusive.

#### Outcomes

The adoption of knowledge products was the commonly measured implementation outcome in 89% (72/81) of SRs [40-50,53,54,56-60,62-77,79-84,86-88,90-95,97-113,115-120], followed by implementation cost in 20% (16/81) of SRs [42,43,47,50,61,73,78,85,87,90,97,99,106,108,112,114], and acceptability in 19% (15/81) of SRs [40,43,47,50,58,61,70,89,96,97,107,108,114,115,118]. Knowledge (19/81, 24%) and attitudes (10/81, 12%) were the other outcomes reported (Table 2). Further details are provided in Multimedia Appendix 5 [40-120].

### Quality Assessment for the Included Reviews

**Figure 2:**
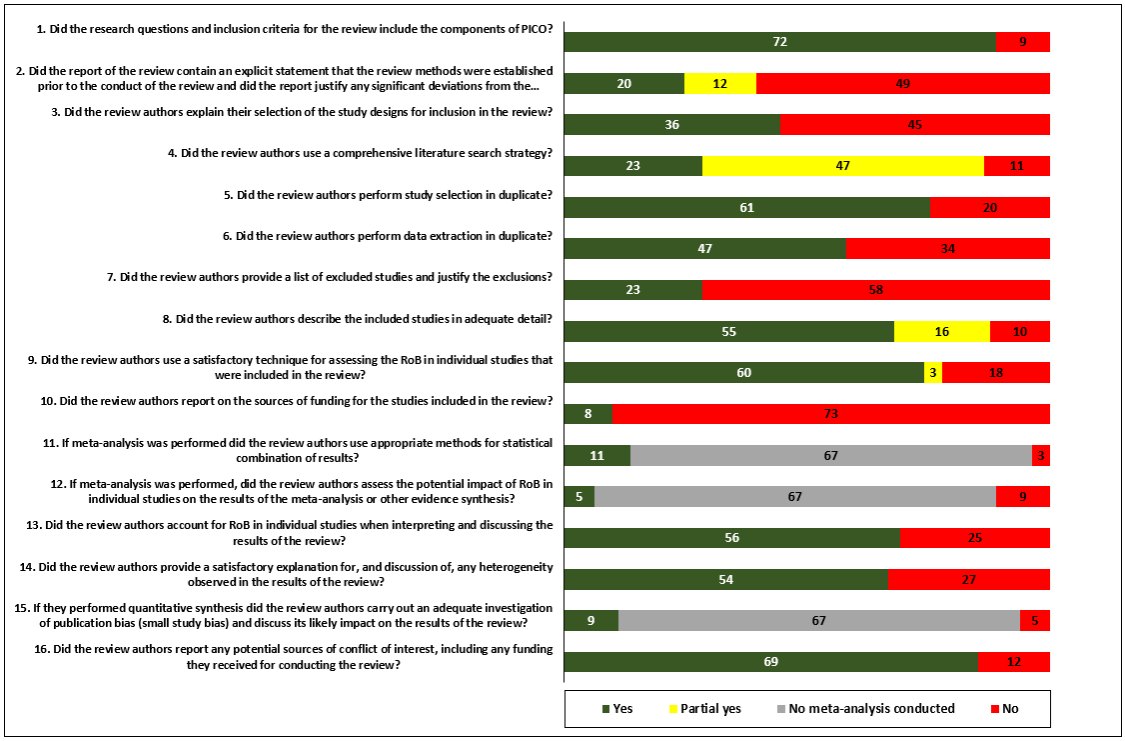
Number of included systematic reviews by A Measurement Tool to Assess Systematic Reviews 2 (AMSTAR 2) methodological quality items and rating scores. PICO: patient or population, intervention, comparison, and outcomes; RoB: risk of bias.

Of the 81 included SRs, 15 (19%) received scores that indicated high or moderate methodological quality [40,44,58,60-65,67,84,87,92,94,108]. The remaining 81% (66/81) received scores that indicated low or very low methodological quality (Table 1). The quality was lowered by 3 criteria: lack of reporting on the funding sources of the studies included in the reviews (73/81, 90%), nonprovision of the list of excluded studies (with reasons for their exclusion; 58/81,72%), and no statement on the existence of the protocol or methodology before the conduct of the review (49/81, 61%; Figure 2). Details for each included SR are provided in Multimedia Appendix 6 [40-120].

### Reported Effectiveness of Implementation Strategies and Level of Evidence of Outcomes in the Included Reviews

We synthesized the evidence by combining information on the reported level of evidence for the outcomes measured, among single knowledge products implemented, and by the implementation strategy used (Table 4). For SRs in which single knowledge products were implemented (56/81, 69%), the level of evidence was reported in 10 SRs with 62 specified outcomes (Table 4). Of these, 50 outcomes were related to the implementation of clinical practice guidelines in 6 SRs [74,77,83,91,110,114], 5 outcomes on management and behavioral or pharmacological health interventions in 3 SRs [60,67,103], and 7 outcomes for health technology interventions or decision support tools in one SR [47] (Table 4).

Regarding clinical practice guidelines (50 outcomes with the level of evidence reported), the following implementation strategies were used: educational material distribution for 42% (21/50) of outcomes [74,77,83,91,110], 71% (15/21) were about adoption [74,77,83,91,110], and 27% (4/15) provided a high or moderate level of evidence [77,91,110]; educational meetings for 30% (15/50) of outcomes [74,77,83,91], all were about adoption, 13% (2/15) provided a high or moderate level of evidence [74,77]; audit and feedback for 26% (13/50) of outcomes [74,77,83,91], all were about adoption, 23% (3/13) of them provided a high or moderate level of evidence [74,77,91]; and reminders for 24% (12/50) of outcomes [77,83,91]; all were about adoption, 33% (4/12) provided a high or moderate level of evidence [77,91] (Table 4).

For management and behavioral or pharmacological health interventions, educational meetings were evaluated for all 100% (5/5) of outcomes [60,67,103], all were about adoption, and 20% (1/5) of them provided a high or moderate level of evidence [103]. Feedback was evaluated for 80% (4/5) of outcomes [60,67,103]; all were about adoption, and 25% (1/4) provided a high or moderate level of evidence [103] (Table 4).

Health technology interventions or decision support tools used reminders for 100% (7/7) of outcomes [47], 43% (3/7) for acceptability [47], with 33% (1/3) providing a high or moderate level of evidence [47]; furthermore, there were 29% (2/7) for implementation costs [47], with 50% (1/2) providing a high or moderate level of evidence [47]. In addition, feedback was used for 86% (6/7) of outcomes [47], 33% (2/6) for acceptability [47], with 50% (1/2) providing a high or moderate level of evidence [47]; furthermore, there were 33% (2/6) for implementation costs [47], with 50% (1/2) providing a high or moderate level of evidence [47] (Table 4).

**Table 4:** Reported level of evidence for measured outcomes for single knowledge products and by implementation strategies used (outcomes: N=62).

Knowledge products, implementation strategies^a^, and categories of outcomes^b,c^			Level of evidence
**Clinical practice guidelines (n=50 outcomes)**			
	**Educational meetings (n=15)**		
		Adoption (n=15)	High (n=1) Moderate (n=1) Low (n=12) Very low (n=1)
	**Educational materials (n=21)**		
		Adoption (n=15)	High (n=1) Moderate (n=3) Low (n=10) Very low (n=1)
		Knowledge (n=3)	Moderate (n=2) Low (n=1)
		Performance in a test situation (n=2)	Low (n=2)
		Satisfaction (n=1)	Low (n=1)
	**Reminders (n=12)**		
		Adoption (n=12)	High (n=1) Moderate (n=3) Low (n=7) Very low (n=1)
	**Educational outreach visits or academic detailing (n=6)**		
		Adoption (n=6)	High (n=1) Moderate (n=1) Low (n=3) Very low (n=1)
	**Audit and feedback (n=13)**		
		Adoption (n=13)	High (n=1) Moderate (n=2) Low (n=9) Very low (n=1)
	**Local opinion leaders (n=4)**		
		Adoption (n=4)	High (n=1) Moderate (n=1) Low (n=2)
	**Feedback (n=12)**		
		Adoption (n=12)	High (n=1) Moderate (n=1) Low (n=9) Very low (n=1)
	**Clinical practice guidelines (n=3)**		
		Adoption (n=3)	High (n=1) Low (n=1) Very low (n=1)
	**Local consensus processes (n=3)**		
		Adoption (n=3)	Moderate (n=1) Low (n=2)
	**Tailored interventions (n=5)**		
		Adoption (n=5)	High (n=1) Moderate (n=2) Low (n=1) Very low (n=1)
	**Audit (n=11)**		
		Adoption (n=11)	High (n=1) Moderate (n=1) Low (n=8) Very low (n=1)
	**Interprofessional education (n=3)**		
		Adoption (n=3)	High (n=1) Low (n=1) Very low (n=1)
	**Continuous quality improvement (n=10)**		
		Adoption (n=10)	High (n=1) Low (n=8) Very low (n=1)
	**Monitoring the performance of the delivery of health care (n=3)**		
		Adoption (n=3)	High (n=1) Low (n=1) Very low (n=1)
**Management and behavioral or pharmacological health interventions (5 outcomes)**			
	**Educational meetings (n=5)**		
		Adoption (n=5)	Moderate (n=1) Low (n=3) Very low (n=1)
	**Educational materials (n=2)**		
		Adoption (n=2)	Moderate (n=1) Very low (n=1)
	**Reminders (n=2)**		
		Adoption (n=2)	Low (n=1) Very low (n=1)
	**Educational outreach visits, or academic detailing (n=2)**		
		Adoption (n=2)	Moderate (n=1) Very low (n=1)
	**Audit and feedback (n=1)**		
		Adoption (n=1)	Very low (n=1)
	**Local opinion leaders (n=1)**		
		Adoption (n=1)	Very low (n=1)
	**Feedback (n=4)**		
		Adoption (n=4)	Moderate (n=1) Low (n=2) Very low (n=1)
	**Local consensus processes (n=2)**		
		Adoption (n=2)	Moderate (n=1) Very low (n=1)
	**Patient-mediated interventions (n=1)**		
		Adoption (n=1)	Moderate (n=1)
**Health technology interventions and decision support tools (7 outcomes)**			
	**Reminders (n=7)**		
		Acceptability (n=3)	Moderate (n=1) Low (n=2)
		Adoption (n=1)	Low (n=1)
		Fidelity (n=1)	Low (n=1)
		Implementation costs (n=2)	Moderate (n=1) Low (n=1)
	**Feedback (n=6)**		
		Acceptability (n=2)	Moderate (n=1) Low (n=1)
		Adoption (n=1)	Low (n=1)
		Fidelity (n=1)	Low (n=1)
		Implementation costs (n=2)	Moderate (n=1) Low (n=1)
	**Clinical practice guidelines (n=1)**		
		Implementation costs (n=1)	Moderate (n=1)

^a^Categories are not mutually exclusive.

^b^Positive outcome (eg, increase in adoption and increase in knowledge).

^c^Within the same review, it may have implemented 1 type of single knowledge product (eg, clinical practice guidelines) but used different specific practices (eg, general obstetric care guidelines and emergency obstetric care guidelines). Although these practices may report the same category of implementation outcome (eg, adoption), if those practices presented and reported different levels of evidence specific for each one (eg, *low* for general obstetric care guidelines and *moderate* for emergency obstetric care guidelines), then their outcomes were extracted separately and analyzed separately.

## Discussion

### Principal Findings

In this paper, we report the results of an SR of SRs, thus providing a detailed portrait of (1) the knowledge products or innovations implemented in primary health care, (2) the implementation strategies used by health care professionals in primary care, and (3) implementation outcomes evaluated as well as their reported level of evidence in primary care.

The findings of this review will be used to inform future SRs of RCTs on the effectiveness of implementation strategies for specific knowledge products.

In this review, which summarized a total of 81 studies, for most (56/81, 69%) of the included SRs, only 1 type of knowledge product (single) was implemented, the majority of which were clinical practice guidelines or health interventions (of management and behavioral or pharmacological nature). Implementation strategies commonly combine education-based strategies (material distributions, meetings, and outreach), reminders, and audits and feedback. Improvement in the adoption of knowledge products was the most measured outcome.

Education-based strategies, audits, feedback, and reminders were mainly used to improve the adoption of clinical practice guidelines and health interventions related to management, behavior, or pharmacology. In contrast, reminders and audit and feedback were used to improve the acceptability and implementation costs of health technology interventions. The reported effectiveness of these strategies was of a high or moderate level of evidence in a few cases and of a low or very low level of evidence in most cases.

### Comparison With Prior Work

Clinical practice guidelines and management, behavioral or pharmacological health interventions, and health technology interventions and decision support tools have been developed to improve clinical practice and patient health outcomes. Despite their comparable effectiveness, the level or degree of implementation varies widely. For instance, as seen in this review, health technology interventions and decision support tools appear to be less implemented or less frequently reported. This does not mean that they are less developed than other knowledge products, but they are probably less commonly addressed in formal research or possibly less known by end users. These interventions, which are generally in the format of mobile-based or computerized-based interventions, are created to accelerate the accessibility and use of KT interventions. A decade ago, such interventions were considered new in the health domain, and it was reasonable that they were minimally implemented [65]. Currently, it is unclear why this situation persists, when health technology interventions and decision support tools are generally recognized as important aspects of care and the way of the future. The reasons may be attributed to the policy-making and funding level (health technologies are often short-term projects, ie, no long-term vision, nonexistence, and unpredictable changes in policies and regulations, financial constraints, eg, affordability, lack of infrastructure [such as office space, supplies, equipment, etc], human resource availability, and digital literacy) [121,122], or the implementation level (unawareness of the technology, perceived usefulness, ie, acceptability, etc) [121,122]. Finally, barriers may differ across settings and cultures [121,122].

The predominance of education-based, reminder, and audit and feedback implementation strategies suggests that they were prioritized based on existing barriers and facilitators of the implementation of the mentioned knowledge products. In fact, some of the most recent systematic and scoping reviews on the topic highlighted a lack of both provider awareness and knowledge of the existence of guidelines, and unfavorable attitudes about them [123-125], in response to which educational and audit and feedback strategies were judged to be suitable [123,125]. In contrast, a lack of access to guidelines and limited time available to providers was also mentioned [124,125], thereby calling for the use of decision support systems or reminders [125]. However, it is important to know whether these strategies are effective in implementing knowledge products. The included SRs demonstrated an *all-directions effect*, which was sometimes consistently positive or negative, or inconsistent, depending on factors such as single versus combined strategies [41,50,71,100] or type of comparator [110]. For example, in a study by Al Zoubi et al [41], single educational strategies appeared to have a small effect, whereas multifaceted strategies that combine educational strategies and other types of strategies, such as reminders, appeared to be more effective, although inconsistent. Kovacs et al [81] found the opposite result, showing that a single intervention is more effective. Others found that effectiveness may depend on the format in which education strategies are delivered; for example, by multimedia and computers [96].

Adoption, which is also referred to as “uptake or utilization,” is “the intention, initial decision, or action to try or employ an innovation or evidence-based practice” [34]. This outcome occurs early or in the middle of the implementation process, is preceded by acceptability and appropriateness, and occurs at the same time as feasibility, followed by fidelity, implementation costs, penetration, and sustainability [34]. All proximal and distal implementation outcomes are important for measurement. The ongoing focus of the literature on the proximal outcome of adoption is more easily understood for recently introduced health technology interventions and strategies but is more difficult to explain when traditional strategies, such as education, are predominantly used.

Regarding the level of evidence of effectiveness, very few SRs have evaluated the level of evidence, as most reviews are narrative. The authors were unable to perform meta-analyses owing to high heterogeneity. In contrast, among the few reviews that assessed the level of evidence, most scored a low or very low grade. This makes it difficult to recognize potentially effective strategies and calls for more methodologically strong SRs to obtain reliable conclusions on the topic.

### Strengths and Limitations

One strength of this SR is its broad objective, which included all EPOC strategies used to implement a variety of health knowledge products, with consideration given to the different implementation outcomes. No type of health care provider was excluded, and even if our target was primary health care, most included SRs covered both primary and secondary health care settings. We did not target any health area. We performed an extensive search and included both Cochrane and non-Cochrane reviews. It has been estimated that including only Cochrane reviews may lead to a loss or change in a median of 31% of the outcome data [126]. The objective of this phase was to characterize rather than measure effectiveness. Both types of SRs offered a large database of 81 reviews for future projects dealing with individual, unique RCTs. Therefore, we believe that our conclusions can be applied to many different contexts.

Few of the included reviews were of high or moderate methodological quality. The criteria lowering the scores may be linked to the unavailability of reporting guidelines at the time of publication or nonadherence to those guidelines when they were available. As per many other overviews, we used the AMSTAR tool and, as suggested, did so in a dual independent team format with a consensus process [25]. It is also possible to exclude reviews based on methodological quality issues when the aim is to produce a detailed picture of a topic [127], as in our case.

With regard to the quality and completeness of the extracted information, in many of the included SRs, the categories of knowledge products, implementation strategies, and outcomes were not reported as per the standard taxonomies used, thereby requiring us to recategorize. This may have introduced some misclassification of information. We addressed this issue and its potential impact on our conclusion by piloting our data extraction process and reaching a consensus for all disagreements (by reviewing the discordant information together).

In the field of overviews, overlapping occurs when one primary study is included in more than one review or when more than one review addresses the same topic [128]. We cannot guarantee that our review will be free of overlapping issues. In addition, we did not evaluate the quality of evidence of outcomes for the included reviews, as we did not intend to demonstrate the effectiveness of the interventions. These 2 issues will be addressed in future projects on the effectiveness of strategies using the design of SRs of individual RCTs. For the comprehensiveness of our strategy, we searched 5 key databases in the field of intervention studies. In addition, we searched the reference lists of all included SRs. However, gray literature was not searched. For efficiency considerations, it was planned to update the search strategy in phase 3 of the project to avoid missing any recently published RCTs on the effectiveness of implementation strategies. We could look for gray literature in this phase.

### Conclusions and Future Directions

Through this SR of SRs, we demonstrated that in the field of implementation, clinical practice guidelines and management, behavioral, or pharmacological health interventions are the most commonly implemented knowledge products, mainly through educational, reminder, and audit and feedback implementation strategies. However, the literature still focuses on the proximal outcomes of improving the adoption of knowledge products, generally with a limited level of evidence.

This SR aimed to provide insight into which knowledge products are frequently implemented, how they are implemented (implementation strategies), and the implementation outcomes measured, rather than providing information on the effectiveness of the implementation strategies. Therefore, in this step, we do not suggest changes in practice; rather, this review provides a good foundation for planning future research on effectiveness. Only detailed and contextualized information on knowledge products and implementation strategies will lead to changes in practice.

We constructed a database of SRs that may be used to strengthen the methodology for the SR of RCTs to overcome the issue of the variable effectiveness of commonly used implementation strategies, such as educational, reminder, and audit and feedback strategies. Future well-designed SRs of RCTs should fully describe the implementation strategy attributes of dose and intensity, format and duration of delivery, geographic location of interventions, and so on. In addition, qualitative studies and reviews involving a variety of collaborators from different domains and levels should be conducted to better understand the barriers and facilitators that contribute to why health technology interventions remain poorly implemented. Future implementation research should explore the entire cascade of implementation outcomes, including proximal and distal outcomes.

## Appendix

Multimedia Appendix 1 Definitions of implementation outcomes.

Multimedia Appendix 2 Search strategy.

Multimedia Appendix 3 Excluded studies and reasons.

Multimedia Appendix 4 General characteristics of included reviews.

Multimedia Appendix 5 Knowledge products, implementation strategies, and outcomes measured in included reviews.

Multimedia Appendix 6 A Measurement Tool to Assess Systematic Reviews 2 quality appraisal of included reviews.

## Abbreviations

AMSTAR: A Measurement Tool to Assess Systematic Reviews
EPOC: Effective Practice and Organization of Care
KT: knowledge translation
PRISMA: Preferred Reporting Items for Systematic Reviews and Meta-Analyses
RCT: randomized controlled trial
SR: systematic review
