# Supplementary material for: Implementation Strategies for Knowledge Products in Primary Health Care: Systematic Review of Systematic Reviews
Source: Interact J Med Res. 2022 Jul 11;11(2):e38419. doi: 10.2196/38419 (PMC9315889; doi:10.2196/38419)
Supplement: Multimedia Appendix 1 [file ijmr_v11i2e38419_app1.docx]

**Definitions of implementation outcomes, based on the Proctor taxonomy**

| **№** | **Implementation outcomes** | **Definition** |
| --- | --- | --- |
| 1 | Acceptability of a knowledge product | - Perception among implementation providers or consumers that the knowledge product was agreeable, palatable, or satisfactory; Or - Implementation providers or consumers satisfaction with various aspects of the knowledge product (e.g. content, complexity, comfort, delivery, and credibility). |
| 2 | Appropriateness of a knowledge product | - For providers or consumers or organization, this is the perceived fit, relevance, or compatibility, suitability; usefulness; practicability of the knowledge product for a given practice setting, provider, or consumer; - And/or perceived fit of the knowledge product to address a particular issue or problem. |
| 3 | Adoption of a knowledge product | - Intention, initial decision, or action by providers/organization to try or employ knowledge product. - Also referred to as uptake; utilization; initial implementation; intention to try. |
| 4 | Feasibility of a knowledge product | - The extent to which a knowledge product has been successfully used or carried out for everyday use (practicability) within a given organization/setting, by providers. |
| 5 | Adherence/fidelity to a knowledge product | - Degree to which providers implemented the knowledge product as intended in the original protocol or in the program design. |
| 6 | Implementation costs of a knowledge product | - The cost impact of an implementation effort, including marginal cost; cost-effectiveness; cost-benefice. |
| 7 | Penetration/reach of a knowledge product | - The integration of a practice within a service setting and its subsystems. Often reported in the literature as level of institutionalization, spread, service access. |
