# Supplementary material for: Implementation Strategies for Knowledge Products in Primary Health Care: Systematic Review of Systematic Reviews
Source: Interact J Med Res. 2022 Jul 11;11(2):e38419. doi: 10.2196/38419 (PMC9315889; doi:10.2196/38419)
Supplement: Multimedia Appendix 2 [file ijmr_v11i2e38419_app2.docx]

**
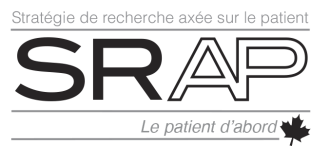

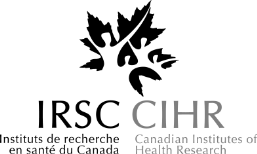
**
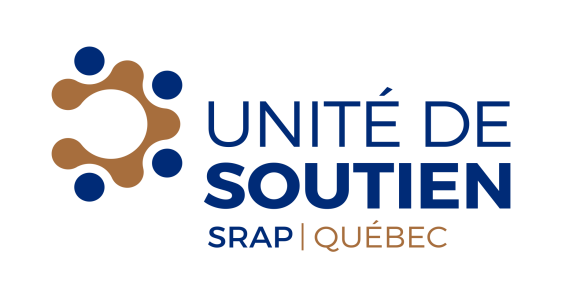


**Revue des revues en transfert des connaissances en santé**

***Stratégie de recherche***

Composante – Recherche sur les systèmes de santé et services sociaux, l’application des connaissances et la mise en œuvre

22 octobre 2019

Nathalie Rheault, M.S.I., Spécialiste de l’information

Roxane Lépine, M.S.I., Spécialiste de l’information

France Légaré

José Massougbodji

Léa Langlois

Michèle Dugas

Ali Ben Charif

Jasmine Sawadogo

Hervé Tchala Vignon Vignon Zomahoun, BSc, MSC, PhD

Table des matières

[Méthodologie 3](#_Toc42608179)

[Sources 4](#_Toc42608180)

[Ovid Medline (2019-10-18) 5](#_Toc42608181)

[Embase (2019-10-18) 11](#_Toc42608182)

[Cinahl (2019-10-17) 16](#_Toc42608183)

[Ovid PsycInfo (2019-10-18) 24](#_Toc42608184)

[Web of Science (2019-10-18) 32](#_Toc42608185)

[Cochrane Library (2019-10-18) 36](#_Toc42608186)

[Consulted Sources 42](#_Toc42608187)

### Méthodologie

Research question: In the context of health care, what is the impact of implementation strategies to increase the application of evidence-based tools?

Population : health professionals

I : implementation strategies

C : Usual practices

O: Outcomes related to patients

S: Primary Care

S: systematic review

Date Limit: Since 2002

### Sources

**Sources utilisées jusqu’à présent**

**Bases de données**

1. Cinahl
2. Cochrane Library
3. Embase
4. Medline
5. PsycInfo
6. Web of Science

**Autres sources**

1. Nous avons retiré les revues incluses dans les revues des revues suivantes :

- Bero 1998
- Brouwers 2011
- Chan 2017
- Cheung 2012
- Durieux 2000
- Durieux 2014
- Freheim 2006
- Getting evidence into practice
- Goldner 2014
- Grimshaw 2001
- Lau 2015
- Lewin 2008
- Pantoja 2014
- Prior 2008

### Ovid Medline (2019-10-18)

| ***Concepts*** | **Research strategy keywords** | **Research** | **# Results** |
| --- | --- | --- | --- |
| Knowledge translation (Controlled vocabulary) | TRANSLATIONAL MEDICAL RESEARCH/ or Information Dissemination/ or exp "diffusion of innovation"/ or exp Education, Continuing/mt, st [Methods, Standards] | #1 | 53 913 |
| Strategies | (strategy or strategies or tool* or framework* or intervention* or program or programs or programme*).ti. or (strategy or strategies or tool* or framework* or intervention* or program or programs or programme*).ab. or (strategy or strategies or tool* or framework* or intervention* or program or programs or programme* ).kf. | #2 | 3 073 185 |
| Knowledge translation strategies | 1 AND 2 | #3 | 17 053 |
| Knowledge translation (Free text) | (("knowledge to action" or "KT" or implementation or implementing or disseminat* ) adj3 (strategy or strategies or tool* or framework* or intervention* or program or programs or programme*) ).ti. or (("knowledge to action" or "KT" or implementation or implementing or disseminat*) adj3 (strategy or strategies or tool* or framework* or intervention* or program or programs or programme*)).ab. or (("knowledge to action" or "KT" or implementation or implementing or disseminat*) adj3 (strategy or strategies or tool* or framework* or intervention* or program or programs or programme*)).kf. | #4 | 33 415 |
|  | (knowledge adj3 (transfer* or translat* or broker* or uptake or "up take" or exchange* or application or utili#ation or cycle or transform* or action or diffusion) adj3 (strategy or strategies or tool* or framework* or intervention* or program or programs or programme*)).ti. or (knowledge adj3 (transfer* or translat* or broker* or uptake or "up take" or exchange* or application or utili#ation or cycle or transform* or action or diffusion) adj3 (strategy or strategies or tool* or framework* or intervention* or program or programs or programme*)).ab. or (knowledge adj3 (transfer* or translat* or broker* or uptake or "up take" or exchange* or application or utili#ation or cycle or transform* or action or diffusion) adj3 (strategy or strategies or tool* or framework* or intervention* or program or programs or programme*) ).kf. | #5 | 1 166 |
|  | (translat* adj3 gap adj3 (strategy or strategies or tool* or framework* or intervention* or program or programs or programme*) ).ti. or (translat* adj3 gap adj3 (strategy or strategies or tool* or framework* or intervention* or program or programs or programme*)).ab. or (translat* adj3 gap adj3 (strategy or strategies or tool* or framework* or intervention* or program or programs or programme*)).kf. | #6 | 6 |
|  | (research adj3 uptake adj3 (strategy or strategies or tool* or framework* or intervention* or program or programs or programme*)).ti. or (research adj3 uptake adj3 (strategy or strategies or tool* or framework* or intervention* or program or programs or programme*)).ab. or (research adj3 uptake adj3 (strategy or strategies or tool* or framework* or intervention* or program or programs or programme*)).kf. | #7 | 19 |
|  | (educational adj3 outreach adj3 (strategy or strategies or tool* or framework* or intervention* or program or programs or programme*) ).ti. or (educational adj3 outreach adj3 (strategy or strategies or tool* or framework* or intervention* or program or programs or programme*)).ab. or (educational adj3 outreach adj3 (strategy or strategies or tool* or framework* or intervention* or program or programs or programme*)).kf. | #8 | 161 |
|  | ((opinion or education* or influential) adj2 (leader or leaders) adj3 (strategy or strategies or tool* or framework* or intervention* or program or programs or programme*) ).ti. or ((opinion or education* or influential) adj2 (leader or leaders) adj3 (strategy or strategies or tool* or framework* or intervention* or program or programs or programme*)).ab. or ((opinion or education* or influential) adj2 (leader or leaders) adj3 (strategy or strategies or tool* or framework* or intervention* or program or programs or programme*)).kf. | #9 | 113 |
|  | (evidence* adj2 practice* adj3 (strategy or strategies or tool* or framework* or intervention* or program or programs or programme*) ).ti. or (evidence* adj2 practice* adj3 (strategy or strategies or tool* or framework* or intervention* or program or programs or programme*)).ab. or (evidence* adj2 practice* adj3 (strategy or strategies or tool* or framework* or intervention* or program or programs or programme*)).kf. | #10 | 737 |
|  | (education* adj1 (intervention* or strateg*) ).ti. or (education* adj1 (intervention* or strateg*)).ab. or (education* adj1 (intervention* or strateg*)).kf. | #11 | 16 020 |
| Knowledge Translation (Free text) | ((evidence or research or knowledge) adj5 (bridge or close) ).ti. or ((evidence or research or knowledge) adj5 (bridge or close)).ab. or ((evidence or research or knowledge) adj5 (bridge or close)).kf. | #12 | 4 147 |
| Strategies (Free text) | (strategy or strategies or tool* or framework* or intervention* or program or programs or programme*).ti. or (strategy or strategies or tool* or framework* or intervention* or program or programs or programme*).ab. or (strategy or strategies or tool* or framework* or intervention* or program or programs or programme*).kf. | #13 | 3 073 185 |
|  | 12 AND 13 | #14 | 1 275 |
| Knowledge translation strategies | 3 OR 4 OR 5 OR 6 OR 7 OR 8 OR 9 OR 10 OR 11 OR 14 | #15 | 66 338 |
| Filter for review (free text) | (bibliographic* or review? or meta-analy* or metaanaly* or overview* or ((research or literature) adj3 synthesis) or ((information or data or evidence*) adj3 synthesis) or (data adj2 extract*)).ti. or (bibliographic* or review? or meta-analy* or metaanaly* or overview* or ((research or literature) adj3 synthesis) or ((information or data or evidence*) adj3 synthesis) or (data adj2 extract*)).ab. or (cinahl or (cochrane adj3 trial*) or embase or medline or psyclit or (psycinfo not "psycinfo database") or pubmed or scopus or "sociological abstracts" or "web of science").ab. or ("cochrane database of systematic reviews" or evidence report technology assessment or evidence report technology assessment summary ).jn. or (review or Meta-Analysis).pt. | #16 | 3 268 268 |
| Filter for review (Controlled vocabulary) | meta-analysis as topic/ or review literature as topic/ | #17 | 23 381 |
| Filter for review | 16 OR 17 | #18 | 3 274 192 |
| Health Professionals (Controlled vocabulary) | exp Professional-Patient Relations/ or exp Health Personnel/ | #19 | 607 089 |
| Health Professionals (Free text) | (nurse* or physician* or clinician* or doctor* or generalist* or practitioner* or provider* or professional* or personnel* or resident* or staff* or team or teams).ti. or (nurse* or physician* or clinician* or doctor* or generalist* or practitioner* or provider* or professional* or personnel* or resident* or staff* or team or teams).ab. or (nurse* or physician* or clinician* or doctor* or generalist* or practitioner* or provider* or professional* or personnel* or resident* or staff* or team or teams).kf. | #20 | 1 615 747 |
| Health Professionals | 19 or 20 | #21 | 1 910 527 |
| Primary Care (Controlled Vocabulary) | Primary Health Care/ OR General Practice/ or Family Practice/ or Physicians, Family/ or General Practitioners/ or Physicians, Primary Care/ or Group practice/ or Ambulatory Care/ OR Community Health Services/ OR Community Health Centers/ or Community Mental Health Services/ or Community Mental Health Centers/ OR RURAL HEALTH SERVICES/ OR NURSE PRACTITIONERS/ or exp Preventive Health Services/ | #22 | 822 310 |
| Primary Care (Free text) | ((primary adj3 care) or ("primary healthcare" or "primary health" or "first line") or ((family or general or group) adj2 (doctor or doctors or physician* or practice* or medicine or nurs*)) or (rural adj3 (physician* or practice or service*)) or generalist* or (ambulatory adj2 (care or clinic)) or (health adj3 (center* or centre*)) or consult* or (visit* adj3 (clinic* or care or outpatient)) or (community adj3 (care or worker* or service* or nurs*)) or "clinical practice*" or (preventive* adj3 (care or cares or medicine* or service* or health*))).ti. or ((primary adj3 care) or ("primary healthcare" or "primary health" or "first line") or ((family or general or group) adj2 (doctor or doctors or physician* or practice* or medicine or nurs*)) or (rural adj3 (physician* or practice or service*)) or generalist* or (ambulatory adj2 (care or clinic)) or (health adj3 (center* or centre*)) or consult* or (visit* adj3 (clinic* or care or outpatient)) or (community adj3 (care or worker* or service* or nurs*)) or "clinical practice*" or (preventive* adj3 (care or cares or medicine* or service* or health*))).ab. or ((primary adj3 care) or ("primary healthcare" or "primary health" or "first line") or ((family or general or group) adj2 (doctor or doctors or physician* or practice* or medicine or nurs*)) or (rural adj3 (physician* or practice or service*)) or generalist* or (ambulatory adj2 (care or clinic)) or (health adj3 (center* or centre*)) or consult* or (visit* adj3 (clinic* or care or outpatient)) or (community adj3 (care or worker* or service* or nurs*)) or "clinical practice*" or (preventive* adj3 (care or cares or medicine* or service* or health*))).kf. | #23 | 689 144 |
| Primary Care | 22 or 23 | #24 | 1 335 458 |
| Total result | #15 AND #18 AND #21 AND #24 | #25 | 3 391 |
| Filter for date | limit 25 to yr="2002 -Current" | #26 | 2 896 |
| **Search strategy run in 2017 March** | | | |
| Knowledge translation strategies | (("knowledge to action" or "KT" or (knowledge adj3 (transfer* or translat* or broker* or mobili#ation or uptake or "up take" or adapt* or implement* or exchange* or application or utili#ation or communicat* or cycle or transform* or action or diffusion or dissemination))) adj3 (strategy or strategies or tool* or framework* or intervention* or program or programs or programme*)).ti. or ( ("knowledge to action" or "KT" or (knowledge adj3 (transfer* or translat* or broker* or mobili#ation or uptake or "up take" or adapt* or implement* or exchange* or application or utili#ation or communicat* or cycle or transform* or action or diffusion or dissemination))) adj3 (strategy or strategies or tool* or framework* or intervention* or program or programs or programme*) ).ab. | #27 | 1 589 |
|  | (((evidence or research or knowledge or theory) adj3 (practice or policy) adj3 gap adj5 (bridge or close)) and (strategy or strategies or tool* or framework* or intervention* or program or programs or programme*)).ti. or (((evidence or research or knowledge or theory) adj3 (practice or policy) adj3 gap adj5 (bridge or close)) and (strategy or strategies or tool* or framework* or intervention* or program or programs or programme*) ).ab. | #28 | 207 |
|  | (implementation adj3 (strategy or strategies or tool* or framework*)).ti. or ("implementation strategy" or "implementation strategies" or "implementation tool*" or (framework adj3 implementation) ).ab. or "implementation program" .ti. or "implementation program".ab. | #29 | 5 050 |
| Knowledge translation strategies | 27 or 28 or 29 | #30 | 6 758 |
| Filter for review (free text) | (bibliographic* or review* or meta-analy* or metaanaly* or ((research or literature) adj3 (overview or synthesis)) or ((information or data) adj3 synthesis) or (data adj2 extract*)).ti. or ( bibliographic* or review* or meta-analy* or metaanaly* or ((research or literature) adj3 (overview or synthesis)) or ((information or data) adj3 synthesis) or (data adj2 extract*) ).ab. or (cinahl or (cochrane adj3 trial*) or embase or medline or psyclit or (psycinfo not "psycinfo database") or pubmed or scopus or "sociological abstracts" or "web of science").ab. or ("cochrane database of systematic reviews" or evidence report technology assessment or evidence report technology assessment summary ).jn. or review.pt. or Meta-Analysis.pt. | #31 | 3 507 194 |
| Filter for review (Controlled vocabulary) | meta-analysis as topic/ or review literature as topic/ | #32 | 23 381 |
| Filter for review | 31 or 32 | #33 | 3 513 049 |
| Total Result | 30 and 33 | #34 | 1 953 |
| Filter for date | limit 33 to yr="1860 - 2015" | #35 | 1 122 |
| **Total number of references to screen** | | | |
| New search strategy without results of the original search | 26 not 35 | #36 | 2 667 |

### Embase (2019-10-18)

| *Concepts* | Research strategy keywords | Research | # Results |
| --- | --- | --- | --- |
| Knowledge translation (Controlled vocabulary) | 'translational research'/exp or 'dissemination'/exp or 'information dissemination'/exp or 'continuing education'/exp | #1 | 67 009 |
| Strategies | (strategy or strategies or tool* or framework* or intervention* or program or programs or programme*):ti,ab,kw | #2 | 4 124 469 |
| Knowledge translation strategies | #1 AND #2 | #3 | 18 585 |
| Knowledge translation (Free text) | (("knowledge to action" or "KT" or implementation or implementing or disseminat*) NEAR/3 (strategy or strategies or tool* or framework* or intervention* or program or programs or programme*)):ti,ab,kw | #4 | 45 639 |
|  | (knowledge NEAR/3 (transfer* or translat* or broker* or uptake or "up take" or exchange* or application or utili?ation or cycle or transform* or action or diffusion) NEAR/3 (strategy or strategies or tool* or framework* or intervention* or program or programs or programme*)):ti,ab,kw | #5 | 1 782 |
|  | (translat* NEAR/3 gap NEAR/3 (strategy or strategies or tool* or framework* or intervention* or program or programs or programme*)):ti,ab,kw | #6 | 10 |
|  | (research NEAR/3 uptake NEAR/3 (strategy or strategies or tool* or framework* or intervention* or program or programs or programme*)):ti,ab,kw | #7 | 31 |
|  | (educational NEAR/3 outreach NEAR/3 (strategy or strategies or tool* or framework* or intervention* or program or programs or programme*)):ti,ab,kw | #8 | 272 |
|  | ((opinion or education* or influential) NEAR/2 (leader or leaders) NEAR/3 (strategy or strategies or tool* or framework* or intervention* or program or programs or programme*)):ti,ab,kw | #9 | 140 |
|  | (evidence* NEAR/2 practice* NEAR/3 (strategy or strategies or tool* or framework* or intervention* or program or programs or programme*)):ti,ab,kw | #10 | 1 144 |
|  | (education* NEAR/1 (intervention* or strateg*)):ti,ab,kw | #11 | 22 986 |
| Knowledge Translation (Free text) | ((evidence or research or knowledge) NEAR/5 (bridge or close)):ti,ab,kw | #12 | 5 323 |
| Strategies (Free text) | (strategy or strategies or tool* or framework* or intervention* or program or programs or programme*):ti,ab,kw | #13 | 4 124 469 |
|  | #12 AND #13 | #14 | 1 748 |
| Knowledge translation strategies | #3 OR #4 OR #5 OR #6 OR #7 OR #8 OR #9 OR #10 OR #11 OR #14 | #15 | 88 501 |
| Filter for review (free text) | (bibliographic* or review? or meta-analy* or metaanaly* or overview* or ((research or literature) NEAR/3 synthesis) or ((information or data or evidence*) NEAR/3 synthesis) or (data NEAR/2 extract*)):ti,ab | #16 | 649 596 |
|  | (cinahl or (cochrane NEAR/3 trial*) or embase or medline or psyclit or (psycinfo not "psycinfo database") or pubmed or scopus or "sociological abstracts" or "web of science"):ab | #17 | 238 285 |
|  | ("cochrane database of systematic reviews" or "evidence report technology assessment" or "evidence report technology assessment summary"):jt | #18 | 21 002 |
|  | (review or Meta-Analysis):it | #19 | 2 526 319 |
|  | #16 OR #17 OR #18 OR #19 | #20 | 2 964 619 |
| Filter for review (Controlled vocabulary) | 'review'/exp OR 'meta analysis'/exp OR 'meta analysis (topic)'/de | #21 | 2 666 917 |
| Filter for review | #20 OR #21 | #22 | 3 129 802 |
| Health Professionals (Controlled vocabulary) | 'health care personnel'/exp OR 'professional-patient relationship'/exp OR 'staff'/exp OR 'multidisciplinary team'/exp | #23 | 1 621 239 |
| Health Professionals (Free text) | (nurse* or physician* or clinician* or doctor* or generalist* or practitioner* or provider* or professional* or personnel* or resident* or staff* or team or teams):ti,ab,kw | #24 | 2 193 117 |
| Health Professionals | #23 or #24 | #25 | 3 000 711 |
| Primary Care (Controlled Vocabulary) | 'ambulatory care'/exp OR 'clinical practice'/de OR 'community care'/exp OR 'community mental health'/exp OR 'community mental health center'/exp OR 'consultation'/exp OR 'family medicine'/exp OR 'general practice'/exp OR 'general practitioner'/exp OR 'group practice'/exp OR 'health center'/exp OR 'preventive medicine'/exp OR 'primary health care'/exp OR 'preventive health service'/de OR 'rural health care'/exp | #26 | 861 047 |
| Primary Care (Free text) | ((primary NEAR/3 care) or ("primary healthcare" or "primary health" or "first line") or ((family or general or group) NEAR/2 (doctor or doctors or physician* or practice* or medicine or nurs*)) or (rural NEAR/3 (physician* or practice or service*)) or generalist* or (ambulatory NEAR/2 (care or clinic)) or (health NEAR/3 (center* or centre*)) or consult* or (visit* NEAR/3 (clinic* or care or outpatient)) or (community NEAR/3 (care or worker* or service* or nurs*)) or "clinical practice*" or (preventive* NEAR/3 (care or cares or medicine* or service* or health*))):ti,ab,kw | #27 | 1 021 553 |
| Primary Care | #26 or #27 | #28 | 1 444 792 |
| Total result | #15 AND #22 AND #25 AND #28 | #29 | 3 209 |
| Filter for date | #15 AND #22 AND #25 AND #28 AND [2002-2019]/py | #30 | 2 890 |
| **Search strategy run in 2017 March** | | | |
| Knowledge translation strategies | (("knowledge to action" or "KT") near/3 (strategy or strategies or tool* or framework* or intervention* OR program OR programs OR programme*)):ti,ab | #31 | 541 |
|  | (knowledge near/3 (transfer* or translat* or broker* or mobilisation or mobilization or uptake or "up take" or adapt* or implement* or exchange* or application or utilisation or utilization or communicat* or cycle or transform* or action or diffusion or dissemination) near/3 (strategy or strategies or tool* or framework* or intervention* or program or programs or programme*)):ti,ab | #32 | 2 080 |
|  | (((evidence or research or knowledge or theory) near/3 (practice or policy) near/3 gap near/5 (bridge or close)) AND (strategy or strategies or tool* or framework* or intervention* OR program OR programs OR programme*)):ti,ab | #33 | 274 |
|  | (implementation near/3 (strategy or strategies or tool* or framework*)):ti | #34 | 1 270 |
|  | ("implementation strategy" or "implementation strategies" or "implementation tool*" or (framework near/3 implementation)):ab | #35 | 5 359 |
|  | "implementation program":ti,ab | #36 | 274 |
| Knowledge translation strategies | #31 OR #32 OR #33 OR #34 OR #35 OR #36 | #37 | 8 901 |
| Filter for review (free text) | (bibliographic* or review* or "meta-analy*" or metaanaly* or ((research or literature) NEAR/3 (overview or synthesis)) or ((information or data) NEAR/3 synthesis) or (data NEAR/2 extract*)):ti,ab | #38 | 2 694 583 |
|  | (cinahl or (cochrane NEAR/3 trial*) or embase or medline or psyclit or (psycinfo not "psycinfo database") or pubmed or scopus or "sociological abstracts" or "web of science"):ab | #39 | 238 285 |
|  | ("cochrane database of systematic reviews" or "evidence report technology assessment" or "evidence report technology assessment summary"):jt | #40 | 21 002 |
|  | review:it | #41 | 2 526 319 |
|  | #38 OR #39 OR #40 OR #41 | #42 | 4 215 165 |
| Filter for review (Controlled vocabulary) | review/de or "meta-analysis (topic)"/de or "Meta Analysis"/de or "systematic review"/de or "systematic review (topic) "/de | #43 | 2 674 388 |
| Filter for review | #42 OR #43 | #44 | 4 339 374 |
| Total Result | #37 AND #44 | #45 | 2 688 |
| Filter for date | #37 AND #44 AND [<1966-2015]/py | #46 | 1 603 |
| **Total number of references to screen** | | | |
| New search strategy without results of the original search | #30 NOT #46 | #47 | 2 633 |

### Cinahl (2019-10-17)

| ***Concepts*** | **Research strategy keywords** | **Research** | **# Results** |
| --- | --- | --- | --- |
| Knowledge translation (Controlled vocabulary) | (MH "Diffusion of Innovation+") OR (MH "Education, Continuing+") | #1 | 43 876 |
| Strategies | TI ( strategy or strategies or tool* or framework* or intervention* or program or programs or programme* )  OR AB ( strategy or strategies or tool* or framework* or intervention* or program or programs or programme* )  OR SU ( strategy or strategies or tool* or framework* or intervention* or program or programs or programme* ) | #2 | 1 169 469 |
| Knowledge translation strategies | S1 AND S2 | #3 | 12 778 |
| Knowledge translation (Free text) | TI ( ("knowledge to action" or "KT" or implementation or implementing or disseminat*) N2 (strategy or strategies or tool* or framework* or intervention* or program or programs or programme*) )  OR AB ( ("knowledge to action" or "KT" or implementation or implementing or disseminat*) N2 (strategy or strategies or tool* or framework* or intervention* or program or programs or programme*) )  OR SU ( ("knowledge to action" or "KT" or implementation or implementing or disseminat*) N2 (strategy or strategies or tool* or framework* or intervention* or program or programs or programme*) ) | #4 | 38 206 |
|  | TI ( (knowledge) N2 (transfer* or translat* or broker* or uptake or "up take" or exchange* or application or utili#ation or cycle or transform* or action or diffusion) N2 (strategy or strategies or tool* or framework* or intervention* or program or programs or programme*) )  OR AB ( (knowledge) N2 (transfer* or translat* or broker* or uptake or "up take" or exchange* or application or utili#ation or cycle or transform* or action or diffusion) N2 (strategy or strategies or tool* or framework* or intervention* or program or programs or programme*) )  OR SU ( (knowledge) N2 (transfer* or translat* or broker* or uptake or "up take" or exchange* or application or utili#ation or cycle or transform* or action or diffusion) N2 (strategy or strategies or tool* or framework* or intervention* or program or programs or programme*) ) | #5 | 745 |
|  | TI ( translat* N2 gap N2 (strategy or strategies or tool* or framework* or intervention* or program or programs or programme*) )  OR AB ( translat* N2 gap N2 (strategy or strategies or tool* or framework* or intervention* or program or programs or programme*) )  OR SU ( translat* N2 gap N2 (strategy or strategies or tool* or framework* or intervention* or program or programs or programme*) ) | #6 | 3 |
|  | TI ( research N2 uptake N2 (strategy or strategies or tool* or framework* or intervention* or program or programs or programme*) )  OR AB ( research N2 uptake N2 (strategy or strategies or tool* or framework* or intervention* or program or programs or programme*) )  OR SU ( research N2 uptake N2 (strategy or strategies or tool* or framework* or intervention* or program or programs or programme*) ) | #7 | 9 |
|  | TI ( educational N2 outreach N2 (strategy or strategies or tool* or framework* or intervention* or program or programs or programme*) )  OR AB ( educational N2 outreach N2 (strategy or strategies or tool* or framework* or intervention* or program or programs or programme*) )  OR SU ( educational N2 outreach N2 (strategy or strategies or tool* or framework* or intervention* or program or programs or programme*) ) | #8 | 90 |
|  | TI ( (opinion or education* or influential) N1 (leader or leaders) N2 (strategy or strategies or tool* or framework* or intervention* or program or programs or programme*) )  OR AB ( (opinion or education* or influential) N1 (leader or leaders) N2 (strategy or strategies or tool* or framework* or intervention* or program or programs or programme*) )  OR SU ( (opinion or education* or influential) N1 (leader or leaders) N2 (strategy or strategies or tool* or framework* or intervention* or program or programs or programme*) ) | #9 | 59 |
|  | TI ( evidence* N1 practice* N2 (strategy or strategies or tool* or framework* or intervention* or program or programs or programme*) )  OR AB ( evidence* N1 practice* N2 (strategy or strategies or tool* or framework* or intervention* or program or programs or programme*) )  OR SU ( evidence* N1 practice* N2 (strategy or strategies or tool* or framework* or intervention* or program or programs or programme*) ) | #10 | 751 |
|  | TI ( education* N0 (intervention* or strateg*) )  OR AB ( education* N0 (intervention* or strateg*) )  OR SU ( education* N0 (intervention* or strateg*) ) | #11 | 10 375 |
| Knowledge Translation (Free text) | TI ( (evidence or research or knowledge) N4 (bridge or close) )  OR AB ( (evidence or research or knowledge) N4 (bridge or close) )  OR SU ( (evidence or research or knowledge) N4 (bridge or close) ) | #12 | 1 258 |
| Strategies (Free text) | TI ( strategy or strategies or tool* or framework* or intervention* or program or programs or programme* )  OR AB ( strategy or strategies or tool* or framework* or intervention* or program or programs or programme* )  OR SU ( strategy or strategies or tool* or framework* or intervention* or program or programs or programme* ) | #13 | 1 169 469 |
|  | S12 AND S13 | #14 | 647 |
| Knowledge translation strategies | S3 OR S4 OR S5 OR S6 OR S7 OR S8 OR S9 OR S10 OR S11 OR S14 | #15 | 60 624 |
| Filter for review (free text) | TI ( bibliographic* or review# or meta-analy* or metaanaly* or overview* or ((research or literature) N2 synthesis) or ((information or data or evidence*) N2 synthesis) or (data N1 extract*) )  OR AB ( bibliographic* or review# or meta-analy* or metaanaly* or overview* or ((research or literature) N2 synthesis) or ((information or data or evidence*) N2 synthesis) or (data N1 extract*) ) | #16 | 497 235 |
|  | AB cinahl or (cochrane N2 trial*) or embase or medline or psyclit or (psycinfo not "psycinfo database") or pubmed or scopus or "sociological abstracts" or "web of science" | #17 | 71 775 |
|  | SO "cochrane database of systematic reviews" or evidence report technology assessment or evidence report technology assessment summary | #18 | 6 023 |
|  | PT review or Meta-Analysis | #19 | 218 209 |
|  | S16 or S17 or S18 or S19 | #20 | 624 544 |
| Filter for review (Controlled vocabulary) | (MH "Literature Review") OR (MH "Systematic Review") OR (MH "Meta Analysis") | #21 | 100 724 |
| Filter for review | S20 or S21 | #22 | 640 266 |
| Health Professionals (Controlled vocabulary) | (MH "Health Personnel+") OR (MH "Professional-Patient Relations+") OR (MH "Multidisciplinary Care Team") | #23 | 601 331 |
| Health Professionals (Free text) | TI ( nurse* or physician* or clinician* or doctor* or generalist* or practitioner* or provider* or professional* or personnel* or resident* or staff* or team or teams )  OR AB ( nurse* or physician* or clinician* or doctor* or generalist* or practitioner* or provider* or professional* or personnel* or resident* or staff* or team or teams )  OR SU ( nurse* or physician* or clinician* or doctor* or generalist* or practitioner* or provider* or professional* or personnel* or resident* or staff* or team or teams ) | #24 | 1 338 622 |
| Health Professionals | S23 OR S24 | #25 | 1 437 465 |
| Primary Care (Controlled Vocabulary) | (MH "Ambulatory Care") OR (MH "Ambulatory Care Facilities+") OR (MH "Ambulatory Care Nursing") OR (MH "Community Health Centers+") OR (MH "Community Health Services+") OR (MH "Community Health Workers") OR (MH "Community Mental Health Services") OR (MH "Family Practice") OR (MH "Group Practice+") OR (MH "Nurse Practitioners") OR (MH "Physicians, Family") OR (MH "Preventive Health Care+") OR (MH "Primary Health Care") OR (MH "Rural Health Centers") OR (MH "Rural Health Personnel") OR (MH "Rural Health Services") | #26 | 527 092 |
| Primary Care (Free text) | TI ( ((primary N2 care) or ("primary healthcare" or "primary health" or "first line") or ((family or general or group) N1 (doctor or doctors or physician* or practice* or medicine or nurs*)) or (rural N2 (physician* or practice or service*)) or generalist* or (ambulatory N1 (care or clinic)) or (health N2 (center* or centre*)) or consult* or (visit* N2 (clinic* or care or outpatient)) or (community N2 (care or worker* or service* or nurs*)) or "clinical practice*" or (preventive* N2 (care or cares or medicine* or service* or health*))) )  OR AB ( ((primary N2 care) or ("primary healthcare" or "primary health" or "first line") or ((family or general or group) N1 (doctor or doctors or physician* or practice* or medicine or nurs*)) or (rural N2 (physician* or practice or service*)) or generalist* or (ambulatory N1 (care or clinic)) or (health N2 (center* or centre*)) or consult* or (visit* N2 (clinic* or care or outpatient)) or (community N2 (care or worker* or service* or nurs*)) or "clinical practice*" or (preventive* N2 (care or cares or medicine* or service* or health*))) )  OR SU ( ((primary N2 care) or ("primary healthcare" or "primary health" or "first line") or ((family or general or group) N1 (doctor or doctors or physician* or practice* or medicine or nurs*)) or (rural N2 (physician* or practice or service*)) or generalist* or (ambulatory N1 (care or clinic)) or (health N2 (center* or centre*)) or consult* or (visit* N2 (clinic* or care or outpatient)) or (community N2 (care or worker* or service* or nurs*)) or "clinical practice*" or (preventive* N2 (care or cares or medicine* or service* or health*))) ) | #27 | 432 816 |
| Primary Care | S26 OR S27 | #28 | 743 933 |
| Total result | S15 AND S22 AND S25 AND S28 | #29 | 2 606 |
| Filter for date | Opérateurs de restriction - Date de publication: 20020101-20191231 | #30 | 2 362 |
| **Search strategy run in 2017 March** | | | |
| Knowledge translation strategies | TI(("knowledge to action" or "KT") N3 (strategy or strategies or tool* or framework* or intervention* OR program OR programs OR programme*))  OR AB( ("knowledge to action" or "KT") N3 (strategy or strategies or tool* or framework* or intervention* OR program OR programs OR programme*) ) | #31 | 262 |
|  | TI(knowledge N3 (transfer* or translat* or broker* or mobili?ation or uptake or "up take" or adapt* or implement* or exchange* or application or utili?ation or communicat* or cycle or transform* or action or diffusion or dissemination) N3 (strategy or strategies or tool* or framework* or intervention* OR program OR programs OR programme*))  OR AB(knowledge N3 (transfer* or translat* or broker* or mobili?ation or uptake or "up take" or adapt* or implement* or exchange* or application or utili?ation or communicat* or cycle or transform* or action or diffusion or dissemination) N3 (strategy or strategies or tool* or framework* or intervention* OR program OR programs OR programme*) ) | #32 | 1 245 |
|  | TI(((evidence or research or knowledge or theory) N3 (practice or policy) N3 gap N5 (bridge or close)) AND (strategy or strategies or tool* or framework* or intervention* OR program OR programs OR programme*))  OR AB(((evidence or research or knowledge or theory) N3 (practice or policy) N3 gap N5 (bridge or close)) AND (strategy or strategies or tool* or framework* or intervention* OR program OR programs OR programme*) ) | #33 | 182 |
|  | TI(implementation N3 (strategy or strategies or tool* or framework*)) or AB("implementation strategy" or "implementation strategies" or "implementation tool*" or (framework N3 implementation) ) | #34 | 3 044 |
|  | TI("implementation program") OR AB("implementation program" ) ) | #35 | 87 |
| Knowledge translation strategies | S31 OR S32 OR S33 OR S34 OR S35 | #36 | 4 551 |
| Filter for review (free text) | TI((bibliographic* or review* or "meta-analy*" or metaanaly* or ((research or literature) N3 (overview or synthesis)) or ((information or data) N3 synthesis) or (data N2 extract*))  or AB((bibliographic* or review* or "meta-analy*" or metaanaly* or ((research or literature) N3 (overview or synthesis)) or ((information or data) N3 synthesis) or (data N2 extract*) ) | #37 | 546 720 |
|  | AB(cinahl or (cochrane N3 trial*) or embase or medline or psyclit or (psycinfo not "psycinfo database") or pubmed or scopus or "sociological abstracts" or "web of science" ) | #38 | 71 831 |
|  | S37 OR S38 | #39 | 553 637 |
| Filter for review (Controlled vocabulary) | MH("Systematic review" or "Scoping review" or "Literature review" "Meta Analysis" ) | #40 | 74 504 |
| Filter for review | S39 OR S40 | #41 | 562 449 |
| Total Result | S36 AND S41 | #42 | 1 137 |
| Filter for date | Opérateurs de restriction - Date de publication: -20160231 | #43 | 605 |
| **Total number of references to screen** | | | |
| New search strategy without results of the original search | S30 NOT S43 | #44 | 2 223 |

### Ovid PsycInfo (2019-10-18)

| ***Concepts*** | **Research strategy keywords** | **Research** | **# Results** |
| --- | --- | --- | --- |
| Knowledge translation (Controlled vocabulary) | continuing education/ or information dissemination/ or knowledge transfer/ | #1 | 5 984 |
| Strategies | (strategy or strategies or tool* or framework* or intervention* or program or programs or programme*).ti.  or (strategy or strategies or tool* or framework* or intervention* or program or programs or programme*).ab.  or (strategy or strategies or tool* or framework* or intervention* or program or programs or programme*).hw. | #2 | 1 113 626 |
| Knowledge translation strategies | 1 AND 2 | #3 | 2 745 |
| Knowledge translation (Free text) | (("knowledge to action" or "KT" or implementation or implementing or disseminat*) adj3 (strategy or strategies or tool* or framework* or intervention* or program or programs or programme*)).ti.  or (("knowledge to action" or "KT" or implementation or implementing or disseminat*) adj3 (strategy or strategies or tool* or framework* or intervention* or program or programs or programme*)).ab.  or (("knowledge to action" or "KT" or implementation or implementing or disseminat*) adj3 (strategy or strategies or tool* or framework* or intervention* or program or programs or programme*)).hw. | #4 | 18 950 |
|  | (knowledge adj3 (transfer* or translat* or broker* or uptake or "up take" or exchange* or application or utili#ation or cycle or transform* or action or diffusion) adj3 (strategy or strategies or tool* or framework* or intervention* or program or programs or programme*)).ti.  or (knowledge adj3 (transfer* or translat* or broker* or uptake or "up take" or exchange* or application or utili#ation or cycle or transform* or action or diffusion) adj3 (strategy or strategies or tool* or framework* or intervention* or program or programs or programme*)).ab.  or (knowledge adj3 (transfer* or translat* or broker* or uptake or "up take" or exchange* or application or utili#ation or cycle or transform* or action or diffusion) adj3 (strategy or strategies or tool* or framework* or intervention* or program or programs or programme*)).hw. | #5 | 544 |
|  | (translat* adj3 gap adj3 (strategy or strategies or tool* or framework* or intervention* or program or programs or programme*)).ti.  or (translat* adj3 gap adj3 (strategy or strategies or tool* or framework* or intervention* or program or programs or programme*)).ab.  or (translat* adj3 gap adj3 (strategy or strategies or tool* or framework* or intervention* or program or programs or programme*)).hw. | #6 | 2 |
|  | (research adj3 uptake adj3 (strategy or strategies or tool* or framework* or intervention* or program or programs or programme*)).ti.  or (research adj3 uptake adj3 (strategy or strategies or tool* or framework* or intervention* or program or programs or programme*)).ab.  or (research adj3 uptake adj3 (strategy or strategies or tool* or framework* or intervention* or program or programs or programme*) ).hw. | #7 | 8 |
|  | (educational adj3 outreach adj3 (strategy or strategies or tool* or framework* or intervention* or program or programs or programme*)).ti.  or (educational adj3 outreach adj3 (strategy or strategies or tool* or framework* or intervention* or program or programs or programme*)).ab.  or (educational adj3 outreach adj3 (strategy or strategies or tool* or framework* or intervention* or program or programs or programme*) ).hw. | #8 | 54 |
|  | ((opinion or education* or influential) adj2 (leader or leaders) adj3 (strategy or strategies or tool* or framework* or intervention* or program or programs or programme*)).ti.  or ((opinion or education* or influential) adj2 (leader or leaders) adj3 (strategy or strategies or tool* or framework* or intervention* or program or programs or programme*)).ab.  or ((opinion or education* or influential) adj2 (leader or leaders) adj3 (strategy or strategies or tool* or framework* or intervention* or program or programs or programme*) ).hw. | #9 | 143 |
|  | (evidence* adj2 practice* adj3 (strategy or strategies or tool* or framework* or intervention* or program or programs or programme*)).ti.  or (evidence* adj2 practice* adj3 (strategy or strategies or tool* or framework* or intervention* or program or programs or programme*)).ab.  or (evidence* adj2 practice* adj3 (strategy or strategies or tool* or framework* or intervention* or program or programs or programme*) ).hw. | #10 | 615 |
|  | (education* adj1 (intervention* or strateg*)).ti.  or (education* adj1 (intervention* or strateg*)).ab.  or (education* adj1 (intervention* or strateg*) ).hw. | #11 | 8 103 |
| Knowledge Translation (Free text) | ((evidence or research or knowledge) adj5 (bridge or close)).ti.  or ((evidence or research or knowledge) adj5 (bridge or close)).ab.  or ((evidence or research or knowledge) adj5 (bridge or close) ).hw. | #12 | 2 938 |
| Strategies (Free text) | (strategy or strategies or tool* or framework* or intervention* or program or programs or programme*).ti.  or (strategy or strategies or tool* or framework* or intervention* or program or programs or programme*).ab.  or (strategy or strategies or tool* or framework* or intervention* or program or programs or programme* ).hw. | #13 | 1 113 626 |
|  | 12 AND 13 | #14 | 1 159 |
| Knowledge translation strategies | 3 OR 4 OR 5 OR 6 OR 7 OR 8 OR 9 OR 10 OR 11 OR 14 | #15 | 31 361 |
| Filter for review (free text) | (bibliographic* or review? or meta-analy* or metaanaly* or overview* or ((research or literature) adj3 synthesis) or ((information or data or evidence*) adj3 synthesis) or (data adj2 extract*) ).ti.  or (bibliographic* or review? or meta-analy* or metaanaly* or overview* or ((research or literature) adj3 synthesis) or ((information or data or evidence*) adj3 synthesis) or (data adj2 extract*) ).ab.  or (cinahl or (cochrane adj3 trial*) or embase or medline or psyclit or (psycinfo not "psycinfo database") or pubmed or scopus or "sociological abstracts" or "web of science" ).ab.  or ("cochrane database of systematic reviews" or evidence report technology assessment or evidence report technology assessment summary ).jn.  or (review or Meta-Analysis).pt. | #16 | 527 061 |
| Filter for review (Controlled vocabulary) | exp "literature review"/ or meta analysis/ | #17 | 26 947 |
| Filter for review | 16 OR 17 | #18 | 532 510 |
| Health Professionals (Controlled vocabulary) | exp health personnel/ | #19 | 156 045 |
| Health Professionals (Free text) | (nurse* or physician* or clinician* or doctor* or generalist* or practitioner* or provider* or professional* or personnel* or resident* or staff* or team or teams).ti.  or (nurse* or physician* or clinician* or doctor* or generalist* or practitioner* or provider* or professional* or personnel* or resident* or staff* or team or teams).ab.  or (nurse* or physician* or clinician* or doctor* or generalist* or practitioner* or provider* or professional* or personnel* or resident* or staff* or team or teams).hw. | #20 | 796 130 |
| Health Professionals | 19 or 20 | #21 | 835 313 |
| Primary Care (Controlled Vocabulary) | clinical practice/ or community mental health centers/ or community mental health services/ or family medicine/ or family physicians/ or general practitioners/ or preventive medicine/ or primary health care/ | #22 | 54 552 |
| Primary Care (Free text) | ((primary adj3 care) or ("primary healthcare" or "primary health" or "first line") or ((family or general or group) adj2 (doctor or doctors or physician* or practice* or medicine or nurs*)) or (rural adj3 (physician* or practice or service*)) or generalist* or (ambulatory adj2 (care or clinic)) or (health adj3 (center* or centre*)) or consult* or (visit* adj3 (clinic* or care or outpatient)) or (community adj3 (care or worker* or service* or nurs*)) or "clinical practice*" or (preventive* adj3 (care or cares or medicine* or service* or health*))).ti.  or ((primary adj3 care) or ("primary healthcare" or "primary health" or "first line") or ((family or general or group) adj2 (doctor or doctors or physician* or practice* or medicine or nurs*)) or (rural adj3 (physician* or practice or service*)) or generalist* or (ambulatory adj2 (care or clinic)) or (health adj3 (center* or centre*)) or consult* or (visit* adj3 (clinic* or care or outpatient)) or (community adj3 (care or worker* or service* or nurs*)) or "clinical practice*" or (preventive* adj3 (care or cares or medicine* or service* or health*))).ab.  or ((primary adj3 care) or ("primary healthcare" or "primary health" or "first line") or ((family or general or group) adj2 (doctor or doctors or physician* or practice* or medicine or nurs*)) or (rural adj3 (physician* or practice or service*)) or generalist* or (ambulatory adj2 (care or clinic)) or (health adj3 (center* or centre*)) or consult* or (visit* adj3 (clinic* or care or outpatient)) or (community adj3 (care or worker* or service* or nurs*)) or "clinical practice*" or (preventive* adj3 (care or cares or medicine* or service* or health*))).hw. | #23 | 214 189 |
| Primary Care | 22 or 23 | #24 | 216 305 |
| Total result | #15 AND #18 AND #21 AND #24 | #25 | 647 |
| Filter for date | limit 25 to yr="2002 -Current" | #26 | 428 |
| **Search strategy run in 2017 March** | | | |
| Knowledge translation strategies | (("knowledge to action" or "KT" or (knowledge adj3 (transfer* or translat* or broker* or mobili#ation or uptake or "up take" or adapt* or implement* or exchange* or application or utili#ation or communicat* or cycle or transform* or action or diffusion or dissemination))) adj3 (strategy or strategies or tool* or framework* or intervention* or program or programs or programme*)).ti,ab. | #27 | 810 |
|  | (((evidence or research or knowledge or theory) adj3 (practice or policy) adj3 gap adj5 (bridge or close)) and (strategy or strategies or tool* or framework* or intervention* or program or programs or programme*)).ti,ab. | #28 | 261 |
|  | (implementation adj3 (strategy or strategies or tool* or framework*)).ti. | #29 | 421 |
|  | ("implementation strategy" or "implementation strategies" or "implementation tool*" or (framework adj3 implementation)).ab. | #30 | 1 723 |
|  | "implementation program".ti,ab. | #31 | 81 |
| Knowledge translation strategies | 27 or 28 or 29 or 30 or 31 | #32 | 3 152 |
| Filter for review (free text) | (bibliographic* or review* or meta-analy* or metaanaly* or ((research or literature) adj3 (overview or synthesis)) or ((information or data) adj3 synthesis) or (data adj2 extract*)).ti,ab. | #33 | 541 353 |
|  | (cinahl or (cochrane adj3 trial*) or embase or medline or psyclit or (psycinfo not "psycinfo database") or pubmed or scopus or "sociological abstracts" or "web of science").ab. | #34 | 22 099 |
|  | 33 or 34 | #35 | 543 348 |
| Filter for review (Controlled vocabulary) | "literature review"/ or meta analysis/ | #36 | 26 823 |
| Filter for review | 35 or 36 | #37 | 547 829 |
| Total Result | 32 and 37 | #38 | 602 |
| Filter for date | limit 38 to yr="1860 - 2015" | #39 | 359 |
| **Total number of references to screen** | | | |
| New search strategy without results of the original search | 26 not 39 | #40 | 376 |

### Web of Science (2019-10-18)

| ***Concepts*** | **Research strategy keywords** | **Research** | **# Results** |
| --- | --- | --- | --- |
| Knowledge translation (Free text) | TS=(("knowledge to action" or "KT" or implementation or implementing or disseminat*) NEAR/2 (strategy or strategies or tool* or framework* or intervention* or program or programs or programme*)) | #1 | 121 237 |
|  | TS=(knowledge NEAR/2 (transfer* or translat* or broker* or uptake or "up take" or exchange* or application or utilisation or utilization or cycle or transform* or action or diffusion) NEAR/2 (strategy or strategies or tool* or framework* or intervention* or program or programs or programme*)) | #2 | 2 347 |
|  | TS=(translat* NEAR/2 gap NEAR/2 (strategy or strategies or tool* or framework* or intervention* or program or programs or programme*)) | #3 | 13 |
|  | TS=(research NEAR/2 uptake NEAR/2 (strategy or strategies or tool* or framework* or intervention* or program or programs or programme*)) | #4 | 22 |
|  | TS=(educational NEAR/2 outreach NEAR/2 (strategy or strategies or tool* or framework* or intervention* or program or programs or programme*)) | #5 | 273 |
|  | TS=((opinion or education* or influential) NEAR/1 (leader or leaders) NEAR/2 (strategy or strategies or tool* or framework* or intervention* or program or programs or programme*)) | #6 | 165 |
|  | TS=(evidence* NEAR/1 practice* NEAR/2 (strategy or strategies or tool* or framework* or intervention* or program or programs or programme*)) | #7 | 879 |
|  | TS=(education* NEAR/0 (intervention* or strateg*)) | #8 | 20 123 |
| Knowledge Translation (Free text) | TS=((evidence or research or knowledge) NEAR/4 (bridge or close)) | #9 | 18 440 |
| Strategies (Free text) | TS=(strategy or strategies or tool* or framework* or intervention* or program or programs or programme*) | #10 | 6 794 156 |
|  | #9 AND #10 | #11 | 5 873 |
| Knowledge translation strategies | #1 or #2 or #3 OR #4 OR #5 OR #6 OR #7 OR #8 OR #11 | #12 | 148 567 |
| Filter for review (free text) | TS=(bibliographic* or review or reviews or meta-analy* or metaanaly* or overview* or ((research or literature) NEAR/2 synthesis) or ((information or data or evidence*) NEAR/2 synthesis) or (data NEAR/1 extract*)) | #13 | 3 026 497 |
|  | TS=(cinahl or (cochrane NEAR/2 trial*) or embase or medline or psyclit or (psycinfo not "psycinfo database") or pubmed or scopus or "sociological abstracts" or "web of science") | #14 | 181 834 |
|  | SO=("cochrane database of systematic reviews" or evidence report technology assessment or evidence report technology assessment summary) | #15 | 11 378 |
| Filter for review | #13 OR #14 OR #15 | #16 | 3 045 769 |
| Health Professionals (Free text) | TS=(nurse* or physician* or clinician* or doctor* or generalist* or practitioner* or provider* or professional* or personnel* or resident* or staff* or team or teams) | #17 | 2 117 377 |
| Primary Care (Free text) | TS=((primary NEAR/2 care) or ("primary healthcare" or "primary health" or "first line") or ((family or general or group) NEAR/1 (doctor or doctors or physician* or practice* or medicine or nurs*)) or (rural NEAR/2 (physician* or practice or service*)) or generalist* or (ambulatory NEAR/1 (care or clinic)) or (health NEAR/2 (center* or centre*)) or consult* or (visit* NEAR/2 (clinic* or care or outpatient)) or (community NEAR/2 (care or worker* or service* or nurs*)) or "clinical practice*" or (preventive* NEAR/2 (care or cares or medicine* or service* or health*))) | #18 | 762 176 |
| Total result | #12 AND #16 AND #17 AND #18 | #19 | 2 694 |
| Filter for date | ESCI Timespan=2002-2019 | #20 | 2 492 |
| **Search strategy run in 2017 March** | | | |
| Knowledge translation strategies | TI=(("knowledge to action" or "KT") near/3 ("strategy" or "strategies" or tool* or framework* or intervention* OR "program" OR "programs" OR programme*)) | #21 | 48 |
|  | TI=("knowledge" near/3 (transfer* or translat* or broker* or "mobili?ation" or implement* or exchange* or "application") near/3 ("strategy" or "strategies" or tool* or framework* or intervention* OR "program" OR "programs" OR programme*)) | #22 | 654 |
|  | TI=(("implementation" near/3 ("strategy" or "strategies" or tool* or framework*)) or "implementation program") | #23 | 4 668 |
| Knowledge translation strategies | #21 AND #22 AND #23 | #24 | 5 332 |
| Filter for review (free text) | TS=(bibliographic* or review* or "meta-analy*" or metaanaly* or (("research" or "literature") NEAR/3 ("overview" or "synthesis")) or (("information" or "data") NEAR/3 "synthesis") or ("data" NEAR/2 extract*) or "cinahl" or ("cochrane" NEAR/3 trial*) or "embase" or "medline" or "psyclit" or ("psycinfo" not "psycinfo database") or "pubmed" or "scopus" or "sociological abstracts" or "web of science") | #25 | 2 839 475 |
| Total Result | #24 AND #25 | #27 | 659 |
| Filter for date | SCI Timespan=2002-2015 | #28 | 323 |
| **Total number of references to screen** | | | |
| New search strategy without results of the original search | #20 not #28 | #29 | 2 446 |

### Cochrane Library (2019-10-18)

| ***Concepts*** | **Research strategy keywords** | **Research** | **# Results** |
| --- | --- | --- | --- |
| Knowledge translation (Controlled vocabulary) | MeSH descriptor: [Translational Medical Research] explode all trees | #1 | 119 |
|  | MeSH descriptor: [Information Dissemination] this term only | #2 | 222 |
|  | MeSH descriptor: [Diffusion of Innovation] explode all trees | #3 | 159 |
|  | MeSH descriptor: [Education, Continuing] explode all trees | #4 | 1 133 |
|  | MeSH descriptor: [Health Plan Implementation] explode all trees | #5 | 156 |
|  | #1 OR #2 OR #3 OR #4 OR #5 | #6 | 1 732 |
| Strategies | (strategy or strategies or tool* or framework* or intervention* or program or programs or programme*):ti,ab,kw | #7 | 455 643 |
| Knowledge translation strategies | #6 AND #7 | #8 | 1 408 |
| Knowledge translation (Free text) | (("knowledge to action" or "KT" or implementation or implementing or disseminat*) NEAR/2 (strategy or strategies or tool* or framework* or intervention* or program or programs or programme*)):ti,ab,kw | #9 | 3 546 |
|  | (knowledge NEAR/2 (transfer* or translat* or broker* or uptake or "up take" or exchange* or application or utili#ation or cycle or transform* or action or diffusion) NEAR/2 (strategy or strategies or tool* or framework* or intervention* or program or programs or programme*)):ti,ab,kw | #10 | 159 |
|  | (translat* NEAR/2 gap NEAR/2 (strategy or strategies or tool* or framework* or intervention* or program or programs or programme*)):ti,ab,kw | #11 | 2 |
|  | (research NEAR/2 uptake NEAR/2 (strategy or strategies or tool* or framework* or intervention* or program or programs or programme*)):ti,ab,kw | #12 | 1 |
|  | (educational NEAR/2 outreach NEAR/2 (strategy or strategies or tool* or framework* or intervention* or program or programs or programme*)):ti,ab,kw | #13 | 36 |
|  | ((opinion or education* or influential) NEAR/1 (leader or leaders) NEAR/2 (strategy or strategies or tool* or framework* or intervention* or program or programs or programme*)):ti,ab,kw | #14 | 27 |
|  | (evidence* NEAR/1 practice* NEAR/2 (strategy or strategies or tool* or framework* or intervention* or program or programs or programme*)):ti,ab,kw | #15 | 21 |
|  | (education* NEAR/0 (intervention* or strateg*)):ti,ab,kw | #16 | 19 |
| Knowledge Translation (Free text) | ((evidence or research or knowledge) NEAR/4 (bridge or close)):ti,ab,kw | #17 | 165 |
| Strategies (Free text) | (strategy or strategies or tool* or framework* or intervention* or program or programs or programme*):ti,ab,kw | #18 | 455 643 |
|  | #17 AND #18 | #19 | 108 |
| Knowledge translation strategies | #8 OR #9 OR #10 OR #11 OR #12 OR #13 OR #14 OR #15 OR #16 OR #19 | #20 | 5 048 |
| Health Professionals (Controlled vocabulary) | MeSH descriptor: [Health Personnel] explode all trees | #21 | 8 271 |
|  | MeSH descriptor: [Professional-Family Relations] this term only | #22 | 202 |
|  | MeSH descriptor: [Professional-Patient Relations] explode all trees | #23 | 2 519 |
|  | MeSH descriptor: [Patient Care Team] explode all trees | #24 | 1 645 |
|  | #21 OR #22 OR #23 OR #24 | #25 | 11 708 |
| Health Professionals (Free text) | (nurse* or physician* or clinician* or doctor* or generalist* or practitioner* or provider* or professional* or personnel* or resident* or staff* or team or teams):ti,ab,kw | #26 | 145 585 |
| Health Professionals | #25 OR #26 | #27 | 147 550 |
| Primary Care (Controlled Vocabulary) | MeSH descriptor: [Primary Health Care] explode all trees | #28 | 6 749 |
|  | MeSH descriptor: [Primary Care Nursing] explode all trees | #29 | 30 |
|  | MeSH descriptor: [Physicians, Primary Care] this term only | #30 | 146 |
|  | MeSH descriptor: [General Practice] explode all trees | #31 | 2 411 |
|  | MeSH descriptor: [General Practitioners] explode all trees | #32 | 245 |
|  | MeSH descriptor: [Physicians, Family] this term only | #33 | 445 |
|  | MeSH descriptor: [Ambulatory Care] explode all trees | #34 | 3 576 |
|  | MeSH descriptor: [Ambulatory Care Facilities] explode all trees | #35 | 1 787 |
|  | MeSH descriptor: [Community Health Services] explode all trees | #36 | 12 947 |
|  | MeSH descriptor: [Community Mental Health Services] this term only | #37 | 702 |
|  | MeSH descriptor: [Community Mental Health Centers] explode all trees | #38 | 111 |
|  | MeSH descriptor: [Rural Health Services] explode all trees | #39 | 336 |
|  | MeSH descriptor: [Nurse Practitioners] explode all trees | #40 | 295 |
|  | MeSH descriptor: [Preventive Medicine] explode all trees | #41 | 180 |
|  | MeSH descriptor: [Preventive Health Services] explode all trees | #42 | 29 825 |
|  | MeSH descriptor: [Family Nursing] this term only | #43 | 36 |
|  | MeSH descriptor: [Group Practice] explode all trees | #44 | 329 |
|  | MeSH descriptor: [Nurses, Community Health] this term only | #45 | 15 |
|  | MeSH descriptor: [Community Health Nursing] explode all trees | #46 | 342 |
|  | MeSH descriptor: [Community Health Workers] this term only | #47 | 434 |
|  | #28 OR #29 OR #30 OR #31 OR #32 OR #33 OR #34 OR #35 OR #36 OR #37 OR #38 OR #39 OR #40 OR #41 OR #42 OR #43 OR #44 OR #45 OR #46 OR #47 | #48 | 48 879 |
| Primary Care (Free text) | ((primary NEAR/2 care) or ("primary healthcare" or "primary health" or "first line") or ((family or general or group) NEAR/1 (doctor or doctors or physician* or practice* or medicine or nurs*)) or (rural NEAR/2 (physician* or practice or service*)) or generalist* or (ambulatory NEAR/1 (care or clinic)) or (health NEAR/2 (center* or centre*)) or consult* or (visit* NEAR/2 (clinic* or care or outpatient)) or (community NEAR/2 (care or worker* or service* or nurs*)) or "clinical practice*" or (preventive* NEAR/2 (care or cares or medicine* or service* or health*))):ti,ab,kw | #49 | 97 081 |
| Primary Care | #48 OR #49 | #50 | 128 675 |
| Total result | #20 AND #27 AND #50 with Cochrane Library publication date from Jan 2002 to Dec 2019 | #51 | 1 694 |
| Reviews^1^ | Cochrane Reviews | - | 33 |
| **Search strategy run in 2017 March** | | | |
| Knowledge translation strategies | (("knowledge to action" or "KT") near/3 ("strategy" or "strategies" or tool* or framework* or intervention* OR "program" OR "programs" OR programme*)):ti,ab,kw | #52 | 102 |
|  | ("knowledge" near/3 (transfer* or translat* or broker* or "mobili?ation" or "uptake" or "up take" or adapt* or implement* or exchange* or "application" or "utili?ation" or communicat* or "cycle" or transform* or "action" or "diffusion" or "dissemination") near/3 ("strategy" or "strategies" or tool* or framework* or intervention* OR "program" OR "programs" OR programme*)):ti,ab,kw | #53 | 240 |
|  | ((("evidence" or "research" or "knowledge" or "theory") near/3 ("practice" or "policy") near/3 "gap" near/5 ("bridge" or "close")) AND ("strategy" or "strategies" or tool* or framework* or intervention* OR "program" OR "programs" OR programme*)):ti,ab,kw | #54 | 14 |
|  | ("implementation" near/3 ("strategy" or "strategies" or tool* or framework*)):ti or (("implementation strategy" or "implementation strategies" or "implementation tool*" or ("framework" near/3 "implementation")):ab,kw or "implementation program":ti,ab,kw) | #55 | 860 |
| Knowledge translation strategies | #52 OR #53 OR #54 OR #55 with Cochrane Library publication date to Feb 2015 | #56 | 286 |
| Reviews^1^ | Cochrane Reviews |  | 2 |
| **Total number of references to screen** | | | |
| New search strategy without results of the original search | #51 NOT #56 | #36 | 1 585 |
| Reviews^1^ | Cochrane Reviews | #37 | 32 |
| ^1^ Pour Cochrane Library, il n’y a pas eu de filtre de revue appliqué en tant que tel. Il a fallu déterminer le nombre de revue pour chaque ligne de recherche en regardant l’onglet *Cochrane Reviews* qui se retrouve en haut à gauche des résultats de recherche. | | | |

### Consulted Sources

Légaré  F, Adekpedjou  R, Stacey  D, Turcotte  S, Kryworuchko  J, Graham  ID, Lyddiatt  A, Politi  MC, Thomson  R, Elwyn  G, Donner‐Banzhoff  N. Interventions for increasing the use of shared decision making by healthcare professionals. Cochrane Database of Systematic Reviews 2018, Issue 7. Art. No.: CD006732. DOI: 10.1002/14651858.CD006732.pub4.
