# Supplementary material for: Implementation Strategies for Knowledge Products in Primary Health Care: Systematic Review of Systematic Reviews
Source: Interact J Med Res. 2022 Jul 11;11(2):e38419. doi: 10.2196/38419 (PMC9315889; doi:10.2196/38419)
Supplement: Multimedia Appendix 3 [file ijmr_v11i2e38419_app3.docx]

**Excluded studies and reasons (N=382)**

| **№** | **Author** | **Year** | **Title** | **Main Reason for exclusion** |
| --- | --- | --- | --- | --- |
| **Principal search** | | | | |
| 1 | Abbas | 2011 | Attitudes of medical students to medical leadership and management: a systematic review to inform curriculum development | Not implementation strategy |
| 2 | Abley | 2019 | Training interventions to improve general hospital care for older people with cognitive impairment: systematic review | Exclusively patients population or general/public/organizational level |
| 3 | Agustinus | 2013 | Factors affecting the attitudes of nurses towards palliative care in the acute and long term care setting: a systematic review | Not implementation strategy |
| 4 | Akbari | 2008 | Interventions to improve outpatient referrals from primary care to secondary care | Knowledge product(s) not specified |
| 5 | Al Daken | 2018 | The implementation of mindfulness-based interventions and educational interventions to support family caregivers of patients with cancer: A systematic review | Knowledge product(s) not specified |
| 6 | Al-Azri | 2014 | Problem-based learning in continuing medical education: Review of randomized controlled trials | Knowledge product(s) not specified |
| 7 | Alberto | 2017 | Screening for sepsis in general hospitalized patients: a systematic review | Exclusively patients population or general/public/organizational level |
| 8 | Alexander | 2012 | Health education strategies used by physical therapists to promote behaviour change in people with lifestyle-related conditions: a systematic review | Exclusively patients population or general/public/organizational level |
| 9 | Alimenti | 2019 | Improving perceptions of patient safety through standardizing handoffs from the emergency department to the inpatient setting: a systematic review | Knowledge product(s) not specified |
| 10 | Allen | 2009 | The Effectiveness of Integrated Care Pathways for Adults and Children in Health Care Settings: A Systematic Review | Exclusively patients population or general/public/organizational level |
| 11 | Altowaijri | 2013 | A systematic review of the clinical and economic effectiveness of clinical pharmacist intervention in secondary prevention of cardiovascular disease | Exclusively patients population or general/public/organizational level |
| 12 | Amin | 2012 | Interventions to enhance community pharmacists' cognitive services: A systematic review | Knowledge product(s) not specified |
| 13 | Anderson | 2017 | Patient education in the management of coronary heart disease | Exclusively patients population or general/public/organizational level |
| 14 | Anderson | 2004 | How can we increase the involvement of primary health care in the treatment of tobacco dependence? A meta-analysis | Knowledge product(s) not specified |
| 15 | Andrade | 2016 | Prevention of catheter-associated urinary tract infection: implementation strategies of international guidelines | Exclusively patients population or general/public/organizational level |
| 16 | Ang | 2018 | Strategies used in improving and assessing the level of reporting of implementation fidelity in randomised controlled trials of palliative care complex interventions: A systematic review | Exclusively patients population or general/public/organizational level |
| 17 | Appiah-Brempong | 2014 | Motivational interviewing interventions and alcohol abuse among college students: a systematic review | Exclusively patients population or general/public/organizational level |
| 18 | Ariyo | 2019 | Implementation strategies to reduce surgical site infections: A systematic review | Exclusively patients population or general/public/organizational level |
| 19 | Arnold | 2005 | Interventions to improve antibiotic prescribing practices in ambulatory care | Knowledge product(s) not specified |
| 20 | Arthurs | 2015 | The effectiveness of therapeutic patient education on adherence to oral anti-cancer medicines in adult cancer patients in ambulatory care settings: A systematic review | Exclusively patients population or general/public/organizational level |
| 21 | Asano | 2014 | Meta-analysis of three different types of fatigue management interventions for people with multiple sclerosis: Exercise, education, and medication | Exclusively patients population or general/public/organizational level |
| 22 | Aung | 2010 | Teaching cause-of-death certification: Lessons from international experience | Knowledge product(s) not specified |
| 23 | Bach-Mortensen | 2018 | Barriers and facilitators to implementing evidence-based interventions among third sector organisations: a systematic review | Exclusively patients population or general/public/organizational level |
| 24 | Baiardini | 2019 | Adherence to treatment in allergic respiratory diseases | Knowledge product(s) not specified |
| 25 | Ballotari | 2017 | Lifestyle-tailored interventions for South Asians with type 2 diabetes living in high-income countries: a systematic review | Exclusively patients population or general/public/organizational level |
| 26 | Balogun | 2017 | Health Facility Staff Training for Improving Breastfeeding Outcome: A Systematic Review for Step 2 of the Baby-Friendly Hospital Initiative | Knowledge product(s) not specified |
| 27 | Baral | 2012 | The Highest Attainable Standard of Evidence (HASTE) for HIV/AIDS Interventions: Toward a Public Health Approach to Defining Evidence | Not implementation strategy |
| 28 | Bartsch | 2016 | A Systematic Literature Review of Self-Reported Smoking Cessation Counseling by Primary Care Physicians | Knowledge product(s) not specified |
| 29 | Batchelor | 2019 | Facilitators and barriers to advance care planning implementation in Australian aged care settings: A systematic review and thematic analysis | Not implementation strategy |
| 30 | Beauchemin | 2019 | Clinical decision support for therapeutic decision-making in cancer: A systematic review | Secondary setting |
| 31 | Bennett | 2011 | Educational interventions by pharmacists to patients with chronic pain: systematic review and meta-analysis | Exclusively patients population or general/public/organizational level |
| 32 | Bergeron | 2017 | Theories, models and frameworks used in capacity building interventions relevant to public health: a systematic review | Not implementation strategy |
| 33 | Bernardes | 2019 | Contemporary Educational Interventions for General Practitioners (GPs) in Primary Care Settings in Australia: A Systematic Literature Review | Knowledge product(s) not specified |
| 34 | Berndt | 2017 | Effectiveness of distance learning strategies for continuing professional development (CPD) for rural allied health practitioners: a systematic review | Knowledge product(s) not specified |
| 35 | Berry | 2008 | The implementation of the NICE guidelines for schizophrenia: barriers to the implementation of psychological interventions and recommendations for the future | Not implementation strategy |
| 36 | Berthelsen | 2015 | The content, dissemination and effects of case management interventions for informal caregivers of older adults: A systematic review | Not implementation strategy |
| 37 | Bhurji | 2016 | Improving management of type 2 diabetes in South Asian patients: a systematic review of intervention studies | Exclusively patients population or general/public/organizational level |
| 38 | Bourcier | 2018 | A systematic review of regulatory and educational interventions to reduce the burden associated with the prescriptions of sedative-hypnotics in adults treated for sleep disorders | Knowledge product(s) not specified |
| 39 | Boyd | 2009 | Interventions for educating children who are at risk of asthma-related emergency department attendance | Exclusively patients population or general/public/organizational level |
| 40 | Boyde | 2011 | Educational interventions for patients with heart failure: a systematic review of randomized controlled trials | Exclusively patients population or general/public/organizational level |
| 41 | Bradford | 2012 | Psychosocial assessments for young people: a systematic review examining acceptability, disclosure and engagement, and predictive utility | Exclusively patients population or general/public/organizational level |
| 42 | Bradley | 2019 | Factors Affecting Pre-Exposure Prophylaxis Implementation for Women in the United States: A Systematic Review | Not implementation strategy |
| 43 | Brennan | 2013 | A systematic review of educational interventions to change behaviour of prescribers in hospital settings, with a particular emphasis on new prescribers | Knowledge product(s) not specified |
| 44 | Bridgwood | 2018 | Interventions for improving modifiable risk factor control in the secondary prevention of stroke | Exclusively patients population or general/public/organizational level |
| 45 | Browne | 2018 | Food and nutrition programs for Aboriginal and Torres Strait Islander Australians: an overview of systematic reviews | Exclusively patients population or general/public/organizational level |
| 46 | Brusamento | 2012 | Assessing the effectiveness of strategies to implement clinical guidelines for the management of chronic diseases at primary care level in EU Member States: A systematic review | No implementation outcomes |
| 47 | Buckley | 2010 | Service organisation for the secondary prevention of ischaemic heart disease in primary care | Exclusively patients population or general/public/organizational level |
| 48 | Byatt | 2015 | Enhancing Participation in Depression Care in Outpatient Perinatal Care Settings: A Systematic Review | Exclusively patients population or general/public/organizational level |
| 49 | Cardona-Morrell | 2010 | Reduction of diabetes risk in routine clinical practice: are physical activity and nutrition interventions feasible and are the outcomes from reference trials replicable? A systematic review and meta-analysis | Exclusively patients population or general/public/organizational level |
| 50 | Cawley | 2010 | Strategies for implementing school-located influenza vaccination of children: a systematic literature review | Exclusively patients population or general/public/organizational level |
| 51 | Cervero | 2015 | The impact of CME on physician performance and patient health outcomes: an updated synthesis of systematic reviews | Not implementation strategy |
| 52 | Chan | 2017 | ACC/AHA Special Report: Clinical Practice Guideline Implementation Strategies: A Summary **of Systematic Reviews** by the NHLBI Implementation Science Work Group | Wrong design |
| 53 | Chawla | 2019 | US Care Pathways: Continued Focus on Oncology and Outstanding Challenges | Not implementation strategy |
| 54 | Cherry | 2012 | Features of educational interventions that lead to compliance with hand hygiene in healthcare professionals within a hospital care setting. A BEME systematic review: BEME Guide No. 22 | Knowledge product(s) not specified |
| 55 | Cheung | 2008 | Review: more high quality studies needed to determine the effects on patient outcomes of psychiatric care guideline implementation | Wrong design |
| 56 | Chi | 2013 | Reducing Alaska Native paediatric oral health disparities: a systematic review of oral health interventions and a case study on multilevel strategies to reduce sugar-sweetened beverage intake | Exclusively patients population or general/public/organizational level |
| 57 | Chong | 2011 | Effectiveness of interventions to improve antidepressant medication adherence: a systematic review | Exclusively patients population or general/public/organizational level |
| 58 | Chopra | 2008 | Effects of policy options for human resources for health: an analysis of systematic reviews | Knowledge product(s) not specified |
| 59 | Chow | 2013 | Effectiveness of Psychoeducational Interventions on Sexual Functioning, Quality of Life and Psychological Outcomes in Patients with Gynecological Cancer | Wrong design |
| 60 | Cicutto | 2003 | Review: self management education improves outcomes in children and adolescents with asthma | Wrong design |
| 61 | Coxon | 2017 | Implementing enhanced recovery pathways: a literature review with realist synthesis | Knowledge product(s) not specified |
| 62 | Cuthbert | 2017 | What is the State of the Science on Physical Activity Interventions for Family Caregivers? A Systematic Review and RE-AIM Evaluation | Not implementation strategy |
| 63 | Cutrona | 2010 | Physician effectiveness in interventions to improve cardiovascular medication adherence: A systematic review | Exclusively patients population or general/public/organizational level |
| 64 | Desplenter | 2006 | The impact of informing psychiatric patients about their medication: a systematic review | Exclusively patients population or general/public/organizational level |
| 65 | Enticott | 2012 | A review on decision support for massive transfusion: Understanding human factors to support the implementation of complex interventions in trauma | Exclusively patients population or general/public/organizational level |
| 66 | Esponda | 2019 | Barriers and facilitators of mental health programmes in primary care in low-income and middle-income countries | Exclusively patients population or general/public/organizational level |
| 67 | Flodgren | 2010 | Interventions to change the behaviour of health professionals and the organisation of care to promote weight reduction in overweight and obese people | Knowledge product(s) not specified |
| 68 | Flodgren | 2017 | Interventions to change the behaviour of health professionals and the organisation of care to promote weight reduction in children and adults with overweight or obesity | Knowledge product(s) not specified |
| 69 | Flodgren | 2016 | Tools developed and disseminated by guideline producers to promote the uptake of their guidelines | No implementation outcomes |
| 70 | Fredericks | 2010 | Effects of the characteristics of teaching on the outcomes of heart failure patient education interventions: a systematic review | Exclusively patients population or general/public/organizational level |
| 71 | Gagnon | 2016 | m-Health adoption by healthcare professionals: a systematic review | Knowledge product(s) not specified |
| 72 | Glenton | 2011 | Can lay health workers increase the uptake of childhood immunisation? Systematic review and typology | Exclusively patients population or general/public/organizational level |
| 73 | Guy | 2011 | Efficacy of interventions to increase the uptake of chlamydia screening in primary care: A systematic review | Knowledge product(s) not specified |
| 74 | Ha | 2018 | Adopting clinical genomics: A systematic review of genomic literacy among physicians in cancer care | Knowledge product(s) not specified |
| 75 | Hall | 2019 | How Should End-of-Life Advance Care Planning Discussions Be Implemented According to Patients and Informal Carers? A Qualitative Review of Reviews | Knowledge product(s) not specified |
| 76 | Harris | 2019 | School-based self-management interventions for asthma in children and adolescents: A mixed methods systematic review | Exclusively patients population or general/public/organizational level |
| 77 | Harris | 2019 | Barriers and facilitators to screening and treating malnutrition in older adults living in the community: a mixed-methods synthesis | Not implementation strategy |
| 78 | Hesselink | 2019 | Geriatric Education Programs for Emergency Department Professionals: A Systematic Review | Knowledge product(s) not specified |
| 79 | Johnson | 2015 | Promoting professional behaviour change in healthcare: what interventions work, and why? A theory-led overview of systematic reviews | Wrong design |
| 80 | Kamarudin | 2013 | Educational interventions to improve prescribing competency: A systematic review | Knowledge product(s) not specified |
| 81 | Kang | 2018 | Discharge education delivered to general surgical patients in their management of recovery post discharge: A systematic mixed studies review | Exclusively patients population or general/public/organizational level |
| 82 | Kaufman | 2018 | Face-to-face interventions for informing or educating parents about early childhood vaccination | Knowledge product(s) not specified |
| 83 | Kaufman | 2013 | Face to face interventions for informing or educating parents about early childhood vaccination | Knowledge product(s) not specified |
| 84 | Kaur | 2009 | Interventions that can reduce inappropriate prescribing in the elderly: a systematic review | Exclusively patients population or general/public/organizational level |
| 85 | Kersten | 2015 | Bridging the gap between goal intentions and actions: a systematic review in patient populations | Exclusively patients population or general/public/organizational level |
| 86 | Kinnersley | 2013 | Interventions to promote informed consent for patients undergoing surgical and other invasive healthcare procedures | Exclusively patients population or general/public/organizational level |
| 87 | Lau | 2016 | Achieving change in primary care--causes of the evidence to practice gap: systematic reviews of reviews | Knowledge product(s) not specified |
| 88 | Liu | 2018 | Effectiveness of Interventions to Decrease Image Ordering for Low Back Pain Presentations in the Emergency Department: A Systematic Review | Exclusively patients population or general/public/organizational level |
| 89 | Lovink | 2017 | Effects of substituting nurse practitioners, physician assistants or nurses for physicians concerning healthcare for the ageing population: a systematic literature review | Knowledge product(s) not specified |
| 90 | Lun Gan | 2011 | Effectiveness of educational interventions to promote oral hypoglycaemic adherence in adults with Type 2 diabetes: a systematic review | Exclusively patients population or general/public/organizational level |
| 91 | Majka | 2014 | Care Coordination to Enhance Management of Long-Term Enteral Tube Feeding: A Systematic Review and Meta-Analysis | Exclusively patients population or general/public/organizational level |
| 92 | McDonagh | 2018 | Interventions to reduce inappropriate prescribing of antibiotics for acute respiratory tract infections: summary and update of a systematic review | Knowledge product(s) not specified |
| 93 | McKinney | 2013 | Simulation-based training for cardiac auscultation skills: Systematic review and meta-analysis | Knowledge product(s) not specified |
| 94 | Naylor | 2012 | Interventions to improve care related to colorectal cancer among racial and ethnic minorities: A systematic review | Exclusively patients population or general/public/organizational level |
| 95 | Neyens | 2011 | Effectiveness and Implementation Aspects of Interventions for Preventing Falls in Elderly People in Long-Term Care Facilities: A Systematic Review of RCTs | Exclusively patients population or general/public/organizational level |
| 96 | Niccolai | 2015 | Practice-and community-based interventions to increase human papillomavirus vaccine coverage a systematic review | Exclusively patients population or general/public/organizational level |
| 97 | Noblet | 2017 | Barriers to and facilitators of independent non-medical prescribing in clinical practice: a mixed-methods systematic review | Not implementation strategy |
| 98 | Parker | 2008 | Effectiveness of interventions that assist caregivers to support people with dementia living in the community: a systematic review | Knowledge product(s) not specified |
| 99 | Peetoom | 2017 | Does well-child care education improve consultations and medication management for childhood fever and common infections? A systematic review | Knowledge product(s) not specified |
| 100 | Pillai Riddell | 2015 | Process Interventions for Vaccine Injections: Systematic Review of Randomized Controlled Trials and Quasi-Randomized Controlled Trials | Knowledge product(s) not specified |
| 101 | Pinnock | 2015 | Implementing supported self-management for asthma: a systematic review and suggested hierarchy of evidence of implementation studies | Exclusively patients population or general/public/organizational level |
| 102 | Quaranta | 2018 | L'aderenza del paziente con malattia renale cronica e in dialisi. Esiti di interventi educativi. Il ruolo dell'Infermiere. Una revisione di revisioni | Exclusively patients population or general/public/organizational level |
| 103 | Ramsey | 2019 | Technology-Based Alcohol Interventions in Primary Care: Systematic Review | No implementation outcomes |
| 104 | Ranji | 2008 | Interventions to reduce unnecessary antibiotic prescribing - A systematic review and quantitative analysis | Knowledge product(s) not specified |
| 105 | Rashid | 2016 | Assessing Interventions To Improve Influenza Vaccine Uptake Among Health Care Workers | Knowledge product(s) not specified |
| 106 | Roque | 2014 | Educational interventions to improve prescription and dispensing of antibiotics: a systematic review | Knowledge product(s) not specified |
| 107 | Roshanov | 2011 | Can computerized clinical decision support systems improve practitioners' diagnostic test ordering behavior? A decision-maker-researcher partnership systematic review | Knowledge product(s) not specified |
| 108 | Rueda | 2006 | Patient support and education for promoting adherence to highly active antiretroviral therapy for HIV/AIDS | Exclusively patients population or general/public/organizational level |
| 109 | Sabatino | 2012 | Effectiveness of interventions to increase screening for breast, cervical, and colorectal cancers: Nine updated systematic reviews for the guide to community preventive services | Knowledge product(s) not specified |
| 110 | Schichtel | 2013 | Educational interventions for primary healthcare professionals to promote the early diagnosis of cancer: a systematic review | Knowledge product(s) not specified |
| 111 | Seidu | 2016 | A systematic review of interventions targeting primary care or community based professionals on cardio-metabolic risk factor control in people with diabetes | Exclusively patients population or general/public/organizational level |
| 112 | Steinman | 2006 | Improving antibiotic selection - A systematic review and quantitative analysis of quality improvement strategies | Knowledge product(s) not specified |
| 113 | Thepwongsa | 2014 | Online continuing medical education (CME) for GPs: does it work? A systematic review | Knowledge product(s) not specified |
| 114 | Vöhringer | 2016 | Healthcare team training programs aimed at improving depression management in primary care: A systematic review | Knowledge product(s) not specified |
| 115 | Bravo | 2008 | Assessing the effectiveness of interventions to promote advance directives among older adults: a systematic review and multi-level analysis | Exclusively patients population or general/public/organizational level |
| 116 | Kinnersley | 2007 | Interventions before consultations for helping patients address their information needs | Knowledge product(s) not specified |
| 117 | Mills | 2018 | Comparative Effectiveness of Implementation Strategies for Blood Pressure Control in Hypertensive Patients: A Systematic Review and Meta-analysis | Exclusively patients population or general/public/organizational level |
| 118 | Mitchell | 2018 | Models of nutrition-focused continuing education programs for nurses: a systematic review of the evidence | Knowledge product(s) not specified |
| 119 | Mostofian | 2015 | Changing Physician Behavior: What Works? | Wrong design |
| 120 | Nguyen | 2019 | Communication training and its effects on carer and care-receiver outcomes in dementia settings: A systematic review | Knowledge product(s) not specified |
| 121 | O'Brien | 2007 | Educational outreach visits: effects on professional practice and health care outcomes | Knowledge product(s) not specified |
| 122 | Oczkowski | 2016 | Communication tools for end-of-life decision-making in ambulatory care settings: A systematic review and meta-analysis | Exclusively patients population or general/public/organizational level |
| 123 | Odone | 2015 | Effectiveness of interventions that apply new media to improve vaccine uptake and vaccine coverage | Knowledge product(s) not specified |
| 124 | Pantoja | 2017 | Implementation strategies for health systems in low-income countries: An overview of systematic reviews | Knowledge product(s) not specified |
| 125 | Parrino | 2005 | Controlled trials to improve antibiotic utilization: A systematic review of experience, 1984-2004 | Knowledge product(s) not specified |
| 126 | Patelarou | 2017 | Approaches to teach evidence-based practice among health professionals: an overview of the existing evidence | No implementation outcomes |
| 127 | Patrick | 2017 | Emergency Physician Training on Mild Traumatic Brain Injury: A Systematic Review | Knowledge product(s) not specified |
| 128 | Pearson | 2009 | Do computerised clinical decision support systems for prescribing change practice? A systematic review of the literature (1990-2007) | Knowledge product(s) not specified |
| 129 | Porter-Armstrong | 2018 | Education of healthcare professionals for preventing pressure ulcers | Knowledge product(s) not specified |
| 130 | Posadzki | 2019 | Offline Digital Education for Postregistration Health Professions: Systematic Review and Meta-Analysis by the Digital Health Education Collaboration | Knowledge product(s) not specified |
| 131 | Ramsay | 2002 | Should health professionals screen women for domestic violence? Systematic review | Knowledge product(s) not specified |
| 132 | Reilly | 2016 | Effectiveness, cost effectiveness, acceptability and implementation barriers/enablers of chronic kidney disease management programs for Indigenous people in Australia, New Zealand and Canada: a systematic review of mixed evidence | Exclusively patients population or general/public/organizational level |
| 133 | Ross | 2016 | Factors that influence the implementation of e-health: a systematic review of systematic reviews (an update) | Exclusively patients population or general/public/organizational level |
| 134 | Ryan | 2011 | Consumer-oriented interventions for evidence-based prescribing and medicines use: an overview of systematic reviews | Knowledge product(s) not specified |
| 135 | Salgado | 2013 | Assessing the Implementability of Clinical Pharmacist Interventions in Patients With Chronic Kidney Disease: An Analysis of Systematic Reviews | Exclusively patients population or general/public/organizational level |
| 136 | Sapkota | 2015 | A systematic review of interventions addressing adherence to anti-diabetic medications in patients with type 2 diabetes - Components of interventions | Exclusively patients population or general/public/organizational level |
| 137 | Senore | 2015 | Optimising colorectal cancer screening acceptance: A review | Knowledge product(s) not specified |
| 138 | Siddiqi | 2005 | Getting evidence into practice: what works in developing countries? | Knowledge product(s) not specified |
| 139 | Simons | 2019 | Does evidence-based medicine training improve doctors' knowledge, practice and patient outcomes? A systematic review of the evidence | No implementation outcomes |
| 140 | Sohn | 2004 | Efficacy of educational interventions targeting primary care providers' practice behaviors: An overview of published systematic reviews | Knowledge product(s) not specified |
| 141 | Sorsdahl | 2009 | Interventions for educating traditional healers about STD and HIV medicine | Knowledge product(s) not specified |
| 142 | Spiby | 2009 | A systematic review of education and evidence-based practice interventions with health professionals and breast feeding counsellors on duration of breast feeding | Knowledge product(s) not specified |
| 143 | Steinberg | 2017 | 2017 - Review: Strategies to improve provider adoption and implementation of clinical practice guidelines were assessed | Wrong design |
| 144 | Stratton | 2019 | Educational interventions for primary care providers to improve clinical skin examination for skin cancer | Knowledge product(s) not specified |
| 145 | Suman | 2016 | Effectiveness of multifaceted implementation strategies for the implementation of back and neck pain guidelines in health care: a systematic review | Exclusively patients population or general/public/organizational level |
| 146 | Takiya | 2004 | Meta-analysis of interventions for medication adherence to antihypertensives | Knowledge product(s) not specified |
| 147 | Tchero | 2017 | Telemedicine in Diabetic Foot Care: A Systematic Literature Review of Interventions and Meta-analysis of Controlled Trials | Knowledge product(s) not specified |
| 148 | Thepwongsa | 2014 | Type 2 diabetes continuing medical education for general practitioners: What works? A systematic review | Knowledge product(s) not specified |
| 149 | Thomas | 2010 | The Effectiveness of Tobacco Intervention Education for Health Professional Students' Health Promotion Practice: A Systematic Review | Wrong design |
| 150 | Thompson | 2011 | Do educational interventions improve nurses' clinical decision making and judgement? A systematic review | Knowledge product(s) not specified |
| 151 | Tomlinson | 2013 | How does tele-learning compare with other forms of education delivery? A systematic review of tele-learning educational outcomes for health professionals | No implementation outcomes |
| 152 | Tonkin-Crine | 2017 | Clinician-targeted interventions to influence antibiotic prescribing behaviour for acute respiratory infections in primary care: An overview of systematic reviews | Knowledge product(s) not specified |
| 153 | Tu | 2002 | Can we alter physician behavior by educational methods? Lessons learned from studies of the management and follow-up of hypertension | Knowledge product(s) not specified |
| 154 | Vaona | 2018 | E-learning for health professionals | Knowledge product(s) not specified |
| 155 | Villa ‐ Roel | 2016 | Effectiveness of Educational Interventions to Increase Primary Care Follow-up for Adults Seen in the Emergency Department for Acute Asthma: A Systematic Review and Meta-analysis | Exclusively patients population or general/public/organizational level |
| 156 | Vodicka | 2013 | Reducing antibiotic prescribing for children with respiratory tract infections in primary care: a systematic review | Knowledge product(s) not specified |
| 157 | Vyas | 2017 | A Systematic Review of the Use of Telemedicine in Plastic and Reconstructive Surgery and Dermatology | Not implementation strategy |
| 158 | Wens | 2008 | Educational interventions aiming at improving adherence to treatment recommendations in type 2 diabetes: a sub-analysis of a systematic review of randomised controlled trials | Exclusively patients population or general/public/organizational level |
| 159 | Yen | 2006 | Engaging physicians to change practice | Knowledge product(s) not specified |
| 160 | Yue | 2017 | The effectiveness of nurse education and training for clinical alarm response and management: a systematic review | Knowledge product(s) not specified |
| 161 | Zavala-González | 2017 | Effectiveness of interventions for improving drug prescribing in Primary Health Care | Knowledge product(s) not specified |
| 162 | Zhelev | 2016 | Effectiveness of interventions to reduce ordering of thyroid function tests: a systematic review | Knowledge product(s) not specified |
| 163 | Zhou | 2016 | The Impact of Project ECHO on Participant and Patient Outcomes: A Systematic Review | Knowledge product(s) not specified |
| 164 | Zhou | 2016 | Interventions to optimise the care continuum for chronic viral hepatitis: a systematic review and meta-analyses | Exclusively patients population or general/public/organizational level |
| 165 | Agrawal | 2016 | The physician payments sunshine act - two years of the open payments program | Wrong design |
| 166 | Amberg | 2009 | FOSTERING IT-BASED INNOVATIONS THROUGH INNOVATION-CONDUCIVE IT PROJECT PORTFOLIO MANAGEMENT | Not implementation strategy |
| 167 | Anderson | 2012 | Nurse Residency Programs: An Evidence-Based Review of Theory, Process, and Outcomes | Not implementation strategy |
| 168 | Behera | 2017 | Strategies for retaining health-care professionals in rural areas of India | No implementation outcomes |
| 169 | Brennan | 2011 | The Map of Medicine: a review of evidence for its impact on healthcare | Not implementation strategy |
| 170 | Brouwers | 2011 | What implementation interventions increase cancer screening rates? a systematic review | Knowledge product(s) not specified |
| 171 | Brown | 2013 | Effect of patient education in the management of coronary heart disease: a systematic review and meta-analysis of randomized controlled trials | Exclusively patients population or general/public/organizational level |
| 172 | Brown | 2016 | Community pharmacy-delivered interventions for public health priorities: A systematic review of interventions for alcohol reduction, smoking cessation and weight management, including meta-analysis for smoking cessation | Exclusively patients population or general/public/organizational level |
| 173 | Bryant | 2014 | A systematic review of the quality of reporting in published smoking cessation trials for pregnant women: an explanation for the evidence-practice gap? | Exclusively patients population or general/public/organizational level |
| 174 | Cherrington | 2008 | Applying the community health worker model to diabetes management: using mixed methods to assess implementation and effectiveness | Knowledge product(s) not specified |
| 175 | Cherry | 2010 | What features of educational interventions lead to competence in aseptic insertion and maintenance of CV catheters in acute care? BEME Guide No. 15 | Knowledge product(s) not specified |
| 176 | Colvin | 2013 | A systematic review of qualitative evidence on barriers and facilitators to the implementation of task-shifting in midwifery services | Not implementation strategy |
| 177 | Coverdale | 2011 | Teaching sexual history-taking: A systematic review of educational programs | Knowledge product(s) not specified |
| 178 | Cusack | 2018 | Educational interventions to improve people's understanding of key concepts in assessing the effects of health interventions: a systematic review | Exclusively patients population or general/public/organizational level |
| 179 | Dawson | 2015 | Approaches to improving the contribution of the nursing and midwifery workforce to increasing universal access to primary health care for vulnerable populations: a systematic review | Exclusively patients population or general/public/organizational level |
| 180 | Dennis | 2008 | Chronic disease management in primary care: from evidence to policy | Exclusively patients population or general/public/organizational level |
| 181 | Deylami | 2018 | Systematic review of publicity interventions to increase awareness amongst healthcare professionals and the public to promote earlier diagnosis of type 1 diabetes in children and young people | Knowledge product(s) not specified |
| 182 | Dhippayom | 2014 | How diabetes risk assessment tools are implemented in practice: A systematic review | Knowledge product(s) not specified |
| 183 | El‐Jardali | 2019 | Barriers and facilitators to implementation of essential health benefits package within primary health care settings in low-income and middle-income countries: A systematic review | Not implementation strategy |
| 184 | Fahey | 2005 | Educational and organisational interventions used to improve the management of hypertension in primary care: a systematic review | Exclusively patients population or general/public/organizational level |
| 185 | Gagliardi | 2016 | Patient-mediated knowledge translation (PKT) interventions for clinical encounters: a systematic review | Exclusively patients population or general/public/organizational level |
| 186 | Ghisi | 2014 | A systematic review of patient education in cardiac patients: do they increase knowledge and promote health behavior change? | Exclusively patients population or general/public/organizational level |
| 187 | Gibson | 2015 | Enablers and barriers to the implementation of primary health care interventions for Indigenous people with chronic diseases: a systematic review | Not implementation strategy |
| 188 | Gold | 2006 | How can osteoporosis patients benefit more from their therapy? Adherence issues with bisphosphonate therapy | Exclusively patients population or general/public/organizational level |
| 189 | Goutier | 2014 | Strategies to Enhance Adoption of Ventilator-Associated Pneumonia Prevention Interventions: A Systematic Literature Review | Knowledge product(s) not specified |
| 190 | Adam | 2015 | Educational interventions for cancer pain. A systematic review of systematic reviews with nested narrative review of randomized controlled trials | Wrong design |
| 191 | Anderson | 2015 | Community coalition-driven interventions to reduce health disparities among racial and ethnic minority populations | Exclusively patients population or general/public/organizational level |
| 192 | Aslakson | 2016 | Assessment tools for palliative care | Not implementation strategy |
| 193 | Audibert | 2017 | No. 348-Joint SOGC-CCMG Guideline: Update on Prenatal Screening for Fetal Aneuploidy, Fetal Anomalies, and Adverse Pregnancy Outcomes | Exclusively patients population or general/public/organizational level |
| 194 | Baker | 2017 | Clinical and cost effectiveness of nurse-led self-management interventions for patients with copd in primary care: A systematic review | Exclusively patients population or general/public/organizational level |
| 195 | Banbury | 2014 | Rapid review of applications of e-health and remote monitoring for rural residents | Not implementation strategy |
| 196 | Bird | 2019 | Moving stroke rehabilitation evidence into practice: a systematic review of randomized controlled trials | Secondary setting |
| 197 | Brody | 2013 | A Review of Interprofessional Dissemination and Education Interventions for Recognizing and Managing Dementia | Knowledge product(s) not specified |
| 198 | Bryant | 2013 | The evidence-practice gap in oncology: Are we contributing to implementation science? | Knowledge product(s) not specified |
| 199 | Burstein | 2019 | Communication interventions to promote the public's awareness of antibiotics: a systematic review | Exclusively patients population or general/public/organizational level |
| 200 | Cadogan | 2015 | The effectiveness of interventions to improve laboratory requesting patterns among primary care physicians: a systematic review | Knowledge product(s) not specified |
| 201 | Cant | 2010 | Simulation-based learning in nurse education: systematic review | Not implementation strategy |
| 202 | Carson | 2012 | Training health professionals in smoking cessation | No implementation outcomes |
| 203 | Carter | 2005 | Postnatal home visits from healthcare professionals show promise for preventing postnatal depression | Exclusively patients population or general/public/organizational level |
| 204 | Catling-Paull | 2011 | Non-clinical interventions that increase the uptake and success of vaginal birth after caesarean section: a systematic review | Knowledge product(s) not specified |
| 205 | Cheng | 2014 | Technology-enhanced simulation and pediatric education: A meta-analysis | Knowledge product(s) not specified |
| 206 | Ciciriello | 2013 | Multimedia educational interventions for consumers about prescribed and over-the-counter medications | Exclusively patients population or general/public/organizational level |
| 207 | Colthart | 2008 | The effectiveness of self-assessment on the identification of learner needs, learner activity, and impact on clinical practice: BEME Guide no. 10 | Not implementation strategy |
| 208 | Cooper Robbins | 2011 | School-based vaccination: a systematic review of process evaluations | Not implementation strategy |
| 209 | Coster | 2009 | Cochrane reviews of educational and self-management interventions to guide nursing practice: a review | Exclusively patients population or general/public/organizational level |
| 210 | Couper | 2013 | Debriefing to improve outcomes from critical illness: A systematic review and meta-analysis | Knowledge product(s) not specified |
| 211 | Covvey | 2019 | Barriers and facilitators to shared decision-making in oncology: a systematic review of the literature | Not implementation strategy |
| 212 | Dadich | 2010 | From bench to bedside: Methods that help clinicians use evidence-based practice | Wrong design |
| 213 | Dave | 2019 | Which community-based HIV initiatives are effective in achieving UNAIDS 90-90-90 targets? A systematic review and meta-analysis of evidence (2007-2018) | No implementation outcomes |
| 214 | Davis | 2018 | A systematic review of clinic and community intervention to increase fecal testing for colorectal cancer in rural and low-income populations in the United States - How, what and when? | Exclusively patients population or general/public/organizational level |
| 215 | Davy | 2015 | Factors influencing the implementation of chronic care models: A systematic literature review | Not implementation strategy |
| 216 | de Silva | 2016 | Community-based population-level interventions for promoting child oral health | Exclusively patients population or general/public/organizational level |
| 217 | Dean | 2010 | A systematic review of interventions to enhance medication adherence in children and adolescents with chronic illness | Exclusively patients population or general/public/organizational level |
| 218 | Dobler | 2019 | Impact of decision aids used during clinical encounters on clinician outcomes and consultation length: a systematic review | Not implementation strategy |
| 219 | Droste | 2014 | Review article: Emergency department data sharing to reduce alcohol-related violence: a systematic review of the feasibility and effectiveness of community-level interventions | Secondary setting |
| 220 | Easthall | 2013 | A meta-analysis of cognitive-based behaviour change techniques as interventions to improve medication adherence | Exclusively patients population or general/public/organizational level |
| 221 | Feltner | 2014 | Transitional care interventions to prevent readmissions for persons with heart failure: a systematic review and meta-analysis | Exclusively patients population or general/public/organizational level |
| 222 | Flynn | 2015 | Primary Care Interventions to Prevent or Treat Traumatic Stress in Childhood: A Systematic Review | Not implementation strategy |
| 223 | Francke | 2008 | Factors influencing the implementation of clinical guidelines for health care professionals: A systematic meta-review | No implementation outcomes |
| 224 | French | 2019 | Barriers and facilitators in understanding ADHD in primary care | Not implementation strategy |
| 225 | Garrard | 2019 | Comprehensive geriatric assessment in primary care: a systematic review | Exclusively patients population or general/public/organizational level |
| 226 | Garzon-Orjuela | 2018 | Effectiveness of knowledge translation and appropriation social (KT) interventions to clinical practice guidelines to patients and community (systematic review) | Exclusively patients population or general/public/organizational level |
| 227 | Gianola | 2016 | Reporting of rehabilitation intervention for low back pain in randomized controlled trials: Is the treatment fully replicable? | Exclusively patients population or general/public/organizational level |
| 228 | Giguère | 2012 | Printed educational materials: effects on professional practice and healthcare outcomes | Knowledge product(s) not specified |
| 229 | Gillette | 2016 | Inhaler Technique in Children With Asthma: A Systematic Review | Exclusively patients population or general/public/organizational level |
| 230 | Glenton | 2013 | The effects, safety and acceptability of compact, pre-filled, autodisable injection devices when delivered by lay health workers | Exclusively patients population or general/public/organizational level |
| 231 | Goetz | 2013 | The impact of price transparency on medical decisions and practice: A systematic review of the literature | Knowledge product(s) not specified |
| 232 | Goossens | 2018 | Barriers and facilitators to the provision of preconception care by healthcare providers: A systematic review | Not implementation strategy |
| 233 | Gravel | 2006 | Barriers and facilitators to implementing shared decision-making in clinical practice: a systematic review of health professionals' perceptions | Not implementation strategy |
| 234 | Grindrod | 2006 | What interventions should pharmacists employ to impact health practitioners' prescribing practices? | Wrong design |
| 235 | Guo | 2002 | Effects of office system and educational interventions in increasing the delivery of preventive health services: A meta-analysis | Knowledge product(s) not specified |
| 236 | Hamilton | 2017 | Primary care providers' cancer genetic testing-related knowledge, attitudes, and communication behaviors: A systematic review and research agenda | Not implementation strategy |
| 237 | Harrod | 2014 | Interventions for primary prevention of suicide in university and other post-secondary educational settings | Exclusively patients population or general/public/organizational level |
| 238 | Health Quality | 2013 | Electronic tools for health information exchange: an evidence-based analysis | Not implementation strategy |
| 239 | Hitzeman | 2012 | Interventions to increase cervical cancer screening rates | Exclusively patients population or general/public/organizational level |
| 240 | Horodyska | 2015 | Good practice characteristics of diet and physical activity interventions and policies: an umbrella review | Wrong design |
| 241 | Horvath | 2017 | Preterm birth: The role of knowledge transfer and exchange | Wrong design |
| 242 | Househ | 2016 | The role of short messaging service in supporting the delivery of healthcare: An umbrella systematic review | Wrong design |
| 243 | Howells | 2016 | Clinical impact of lifestyle interventions for the prevention of diabetes: an overview of systematic reviews | Exclusively patients population or general/public/organizational level |
| 244 | Hu | 2016 | Interventions to reduce childhood antibiotic prescribing for upper respiratory infections: systematic review and meta-analysis | Knowledge product(s) not specified |
| 245 | Hui | 2014 | Integration of oncology and palliative care (PC): A systematic review | Not implementation strategy |
| 246 | Inghels |  | Effect of organizational models of provider-initiated testing and counseling (PITC) in health facilities on adult HIV testing coverage in sub-Saharan Africa | Exclusively patients population or general/public/organizational level |
| 247 | Jenkins | 2015 | Effectiveness of interventions designed to reduce the use of imaging for low-back pain: a systematic review | Knowledge product(s) not specified |
| 248 | Jennings | 2018 | General practitioners' knowledge, attitudes, and experiences of managing behavioural and psychological symptoms of dementia: A mixed-methods systematic review | Not implementation strategy |
| 249 | Kadu | 2015 | Facilitators and barriers of implementing the chronic care model in primary care: a systematic review | Not implementation strategy |
| 250 | Kahn | 2013 | Interventions for implementation of thromboprophylaxis in hospitalized medical and surgical patients at risk for venous thromboembolism | Other: Old version. An updated version is included. |
| 251 | Kahn | 2018 | Interventions for implementation of thromboprophylaxis in hospitalized patients at risk for venous thromboembolism | Secondary setting |
| 252 | Kaltenthaler | 2014 | The effectiveness of sexual health interventions for people with severe mental illness: A systematic review | No implementation outcomes |
| 253 | Karavetian | 2007 | Dietary educational interventions for management of hyperphosphatemia in hemodialysis patients: a systematic review and meta-analysis | Exclusively patients population or general/public/organizational level |
| 254 | Kim | 2018 | Interventions promoting exclusive breastfeeding up to six months after birth: A systematic review and meta-analysis of randomized controlled trials | Exclusively patients population or general/public/organizational level |
| 255 | Kirolos | 2014 | Interventions to Improve Hospice and Palliative Care Referral: A Systematic Review | Knowledge product(s) not specified |
| 256 | Kominiarek | 2018 | Targeting obstetric providers in interventions for obesity and gestational weight gain: A systematic review | Knowledge product(s) not specified |
| 257 | Kunstler | 2019 | Changing prescribing behaviours with educational outreach: an overview of evidence and practice | Wrong design |
| 258 | Kurlander | 2016 | How Efficacious Are Patient Education Interventions to Improve Bowel Preparation for Colonoscopy? A Systematic Review | Exclusively patients population or general/public/organizational level |
| 259 | Kwint | 2013 | The Relationship between the Extent of Collaboration of General Practitioners and Pharmacists and the Implementation of Recommendations Arising from Medication Review | Knowledge product(s) not specified |
| 260 | Langbecker | 2015 | Systematic review of interventions to improve the provision of information for adults with primary brain tumors and their caregivers | Knowledge product(s) not specified |
| 261 | Lau | 2012 | Impact of electronic medical record on physician practice in office settings: a systematic review | Not implementation strategy |
| 262 | Lavielle | 2018 | Methods to improve medication adherence in patients with chronic inflammatory rheumatic diseases: A systematic literature review | Exclusively patients population or general/public/organizational level |
| 263 | Légaré | 2016 | Improving decision making about genetic testing in the clinic: An overview of effective knowledge translation interventions | Knowledge product(s) not specified |
| 264 | Legare | 2012 | Patients' Perceptions of Sharing in Decisions A Systematic Review of Interventions to Enhance Shared Decision Making in Routine Clinical Practice | Other: Already included by the earlier search |
| 265 | Licqurish | 2018 | Interventions for maximizing quality communication in cancer care: A systematic review of systematic reviews | Wrong design |
| 266 | Liew | 2019 | Reappraising the Efficacy and Acceptability of Multicomponent Interventions for Caregiver Depression in Dementia: The Utility of Network Meta-Analysis | Not implementation strategy |
| 267 | Liu | 2017 | Health education for patients with acute coronary syndrome and type 2 diabetes mellitus: an umbrella review of systematic reviews and meta-analyses | Exclusively patients population or general/public/organizational level |
| 268 | McCall | 2018 | Characteristics and efficacy of digital health education: An overview of systematic reviews | Wrong design |
| 269 | McKillop | 2017 | Understanding the Attributes of Implementation Frameworks to Guide the Implementation of a Model of Community-based Integrated Health Care for Older Adults with Complex Chronic Conditions: A Metanarrative Review | Exclusively patients population or general/public/organizational level |
| 270 | Mesner | 2016 | Implementation interventions to improve the management of non-specific low back pain: a systematic review | Knowledge product(s) not specified |
| 271 | Higgins | 2004 | A systematic review of the effectiveness of interventions to help older people adhere to medication regimes | Exclusively patients population or general/public/organizational level |
| 272 | Hill | 2017 | Roles, responsibilities and characteristics of lay community health workers involved in diabetes prevention programmes: A systematic review | Knowledge product(s) not specified |
| 273 | Hilliard | 2018 | Educating trainees on the use of electronic communication with patients: A systematic review | Not implementation strategy |
| 274 | Hu | 2011 | Barriers to screening for hepatitis B virus infection in Asian Americans | Exclusively patients population or general/public/organizational level |
| 275 | Jordan | 2017 | Implementation strategies for guidelines at ICUs: a systematic review | Secondary setting |
| 276 | Kastner | 2018 | Complex interventions can increase osteoporosis investigations and treatment: a systematic review and meta-analysis | Exclusively patients population or general/public/organizational level |
| 277 | Kim | 2016 | Health-Literacy-Sensitive Diabetes Self-Management Interventions: A Systematic Review and Meta-Analysis | Exclusively patients population or general/public/organizational level |
| 278 | Kwan | 2004 | Improving the efficiency of delivery of thrombolysis for acute stroke: a systematic review | Knowledge product(s) not specified |
| 279 | Lall | 2019 | Influences on the Implementation of Mobile Learning for Medical and Nursing Education: Qualitative Systematic Review by the Digital Health Education Collaboration | No implementation outcomes |
| 280 | Lewis | 2019 | Barriers and facilitators of pediatric shared decision-making: a systematic review | Not implementation strategy |
| 281 | Long | 2018 | Barriers and Facilitators of Engaging Community Health Workers in Non-Communicable Disease (NCD) Prevention and Control in China: A Systematic Review (2006-2016) | Exclusively patients population or general/public/organizational level |
| 282 | Lumbiganon | 2012 | Antenatal breastfeeding education for increasing breastfeeding duration | Exclusively patients population or general/public/organizational level |
| 283 | Maritim | 2019 | Factors shaping the implementation of the SAFE strategy for trachoma using the Consolidated Framework for Implementation Research: a systematic review | No implementation outcomes |
| 284 | Meiklejohn | 2016 | A Systematic Review of the Impact of Multi-Strategy Nutrition Education Programs on Health and Nutrition of Adolescents | No implementation outcomes |
| 285 | Pazol | 2015 | Impact of Contraceptive Education on Contraceptive Knowledge and Decision Making A Systematic Review | Knowledge product(s) not specified |
| 286 | Pazol | 2018 | Impact of Contraceptive Education on Knowledge and Decision Making: An Updated Systematic Review | Exclusively patients population or general/public/organizational level |
| 287 | Reisman | 2016 | Newborn Resuscitation Training in Resource-Limited Settings: A Systematic Literature Review | Knowledge product(s) not specified |
| 288 | Robinson | 2008 | Review article: improving adherence to medication in patients with inflammatory bowel disease | Exclusively patients population or general/public/organizational level |
| 289 | Schaepe | 2015 | Educational interventions in peritoneal dialysis: A narrative review of the literature | No implementation outcomes |
| 290 | Silverberg | 2017 | A review of antimicrobial stewardship training in medical education | Knowledge product(s) not specified |
| 291 | Simpson | 2005 | Do guidelines guide pneumonia practice? A systematic review of interventions and barriers to best practice in the management of community-acquired pneumonia | Knowledge product(s) not specified |
| 292 | Sjoding | 2017 | Translating evidence into practice in acute respiratory distress syndrome: Teamwork, clinical decision support, and behavioral economic interventions | No implementation outcomes |
| 293 | Soril | 2018 | Behaviour modification interventions to optimise red blood cell transfusion practices: A systematic review and meta-analysis | Knowledge product(s) not specified |
| 294 | Squires | 2014 | Are multifaceted interventions more effective than single-component interventions in changing health-care professionals' behaviours? An overview of systematic reviews | Wrong design |
| 295 | Stander | 2018 | Training programmes to improve evidence uptake and utilisation by physiotherapists: a systematic scoping review | Knowledge product(s) not specified |
| 296 | Turrillas | 2018 | A Systematic Review of Training in Symptom Management in Palliative Care Within Postgraduate Medical Curriculums | Knowledge product(s) not specified |
| 297 | Ugalde | 2019 | A systematic review of cancer caregiver interventions: Appraising the potential for implementation of evidence into practice | Not implementation strategy |
| 298 | Urquhart | 2009 | Nursing record systems: effects on nursing practice and healthcare outcomes | Not implementation strategy |
| 299 | Vollmar | 2016 | Interventions to improve primary dementia care | No implementation outcomes |
| 300 | Wagle | 2018 | Caregiver-Based Interventions to Optimize Medication Safety in Vulnerable Elderly Adults: A Systematic Evidence-Based Review | Knowledge product(s) not specified |
| 301 | Walsh | 2016 | Effectiveness of the STOPP/START (Screening tool of older persons' potentially inappropriate prescriptions/ screening tool to alert doctors to the right treatment) criteria: Systematic review and meta-analysis of randomized controlled studies | Exclusively patients population or general/public/organizational level |
| 302 | Walters | 2005 | Effectiveness of workshop training for psychosocial addiction treatments: A systematic review | Knowledge product(s) not specified |
| 303 | Wand | 2011 | Evaluating the effectiveness of educational interventions to prevent delirium | Knowledge product(s) not specified |
| 304 | Wang | 2016 | Increasing Coverage of Hepatitis B Vaccination in China: A Systematic Review of Interventions and Implementation Experiences | No implementation outcomes |
| 305 | Ying | 2018 | Effect of multicomponent interventions on competence of family caregivers of people with dementia: A systematic review | Knowledge product(s) not specified |
| **Search in references** | | | | |
| 306 | Akl | 2013 | Educational games for health professionals | Knowledge product(s) not specified |
| 307 | Amin | 2012 | Strategies to optimize the prevention of venous thromboembolism: process improvement practices | Knowledge product(s) not specified |
| 308 | Arditi | 2017 | Computer-generated reminders delivered on paper to healthcare professionals; effects on professional practice and health care outcomes | Knowledge product(s) not specified |
| 309 | Austin | 2015 | Tools to Promote Shared Decision Making in Serious Illness A Systematic Review | Not implementation strategy |
| 310 | Baker | 2010 | Tailored interventions to overcome identified barriers to change: effects on professional practice and health care outcomes | Knowledge product(s) not specified |
| 311 | Berube | 2018 | Strategies to translate knowledge related to common musculoskeletal conditions into physiotherapy practice: a systematic review | Knowledge product(s) not specified |
| 312 | Bighelli | 2016 | Implementation of treatment guidelines for specialist mental health care | Exclusively patients population or general/public/organizational level |
| 313 | Borab | 2017 | Use of Computerized Clinical Decision Support Systems to Prevent Venous Thromboembolism in Surgical Patients A Systematic Review and Meta-analysis | Exclusively patients population or general/public/organizational level |
| 314 | Boss | 2016 | Shared Decision Making and Choice for Elective Surgical Care: A Systematic Review | Secondary setting |
| 315 | Bower | 2005 | Managing common mental health disorders in primary care: conceptual models and evidence base | Wrong design |
| 316 | Bright | 2012 | Effect of clinical decision-support systems: a systematic review | Knowledge product(s) not specified |
| 317 | Cahill | 2009 | Bridging the Guideline-Practice Gap in Critical Care Nutrition: A Review of Guideline Implementation Studies | Secondary setting |
| 318 | Davey | 2013 | Interventions to improve antibiotic prescribing practices for hospital inpatients | Secondary setting |
| 319 | Davis | 1995 | Changing physician performance. A systematic review of the effect of continuing medical education strategies | No implementation outcomes |
| 320 | Delpierre | 2004 | A systematic review of computer-based patient record systems and quality of care: more randomized clinical trials or a broader approach? | Knowledge product(s) not specified |
| 321 | Donnellan. | 2013 | Health professionals' adherence to stroke clinical guidelines: A review of the literature | Wrong design |
| 322 | Doumit | 2007 | Local opinion leaders: effects on professional practice and health care outcomes | Knowledge product(s) not specified |
| 323 | Flodgren. | 2011 | Local opinion leaders: effects on professional practice and health care outcomes | Knowledge product(s) not specified |
| 324 | Forsetlund | 2009 | Continuing education meetings and workshops: effects on professional practice and health care outcomes | Knowledge product(s) not specified |
| 325 | Gagnon | 2016 | A systematic review of knowledge translation (KT) in pediatric pain: Focus on health care providers. | Secondary setting |
| 326 | Garg | 2005 | Effects of computerized clinical decision support systems on practitioner performance and patient outcomes - A systematic review | Knowledge product(s) not specified |
| 327 | Giguere | 2012 | Printed educational materials: effects on professional practice and healthcare outcomes | Knowledge product(s) not specified |
| 328 | Greenhalgh | 2004 | Diffusion of innovations in service organizations: Systematic review and recommendations | Exclusively patients population or general/public/organizational level |
| 329 | Griffin | 2004 | Effect on health-related outcomes of interventions to alter the interaction between patients and practitioners: A systematic review of trials | Not implementation strategy |
| 330 | Grimshaw | 1993 | Effect of clinical guidelines on medical practice: a systematic review of rigorous evaluations | Knowledge product(s) not specified |
| 331 | Grimshaw | 2004 | Effectiveness and efficiency of guideline dissemination and implementation strategies | Knowledge product(s) not specified |
| 332 | Grimshaw | 2006 | Toward evidence-based quality improvement. Evidence (and its limitations) of the effectiveness of guideline dissemination and implementation strategies 1966-1998 | Knowledge product(s) not specified |
| 333 | Hakkennes | 2008 | Guideline implementation in allied health professions: a systematic review of the literature | Knowledge product(s) not specified |
| 334 | Heideman | 2005 | Interventions to improve management of anxiety disorders in general practice: a systematic review | No implementation outcomes |
| 335 | Huis | 2012 | A systematic review of hand hygiene improvement strategies: a behavioural approach | Not implementation strategy |
| 336 | Hunt | 1998 | Effects of computer-based clinical decision support systems on physician performance and patient outcomes: a systematic review | Knowledge product(s) not specified |
| 337 | Ivers | 2012 | Audit and feedback: effects on professional practice and healthcare outcomes | Knowledge product(s) not specified |
| 338 | Jamtvedt | 2006 | Audit and feedback: effects on professional practice and health care outcomes | Knowledge product(s) not specified |
| 339 | Kahn | 2018 | Interventions for implementation of thromboprophylaxis in hospitalized medical and surgical patients at risk for venous thromboembolism | Secondary setting |
| 340 | Kaplan | 2001 | Evaluating informatics applications - clinical decision support systems literature review | Knowledge product(s) not specified |
| 341 | Kawamoto | 2003 | Clinical decision support provided within physician order entry systems: a systematic review of features effective for changing clinician behavior | Knowledge product(s) not specified |
| 342 | Kawamoto | 2005 | Improving clinical practice using clinical decision support systems: a systematic review of trials to identify features critical to success | Knowledge product(s) not specified |
| 343 | Kroenke | 2000 | Interventions to improve provider diagnosis and treatment of mental disorders in primary care - A critical review of the literature | Wrong design |
| 344 | LaRocca | 2012 | The effectiveness of knowledge translation strategies used in public health: a systematic review | No implementation outcomes |
| 345 | Lewin | 2001 | Interventions for providers to promote a patient-centred approach in clinical consultations | Secondary setting |
| 346 | Middleton | 2011 | Implementation of evidence-based treatment protocols to manage fever, hyperglycaemia, and swallowing dysfunction in acute stroke (QASC): a cluster randomised controlled trial | Exclusively patients population or general/public/organizational level |
| 347 | Mugford | 1991 | EFFECTS OF FEEDBACK OF INFORMATION ON CLINICAL-PRACTICE - A REVIEW | Wrong design |
| 348 | Neumeyer-Gromen | 2004 | Disease management programs for depression - A systematic review and meta-analysis of randomized controlled trials | No implementation outcomes |
| 349 | Noonan | 2014 | Knowledge translation and implementation in spinal cord injury: a systematic review | Knowledge product(s) not specified |
| 350 | Novins | 2013 | Dissemination and implementation of evidence-based practices for child and adolescent mental health: a systematic review | Knowledge product(s) not specified |
| 351 | O'Brien | 2007 | Educational outreach visits: effects on professional practice and health care outcomes | Knowledge product(s) not specified |
| 352 | Oxman | 1995 | No magic bullets: a systematic review of 102 trials of interventions to improve professional practice | Knowledge product(s) not specified |
| 353 | Patwardhan | 2014 | Intervention research to enhance community pharmacists' cognitive services: A systematic review | Knowledge product(s) not specified |
| 354 | Pawloski | 2019 | A Systematic Review of Clinical Decision Support Systems for Clinical Oncology Practice | Secondary setting |
| 355 | Randell | 2007 | Effects of computerized decision support systems on nursing performance and patient outcomes: a systematic review | Knowledge product(s) not specified |
| 356 | Rao | 2007 | Communication interventions make a difference in conversations between physicians and patients - A systematic review of the evidence | Not implementation strategy |
| 357 | Shojania | 2009 | The effects of on-screen, point of care computer reminders on processes and outcomes of care. (Review) | Knowledge product(s) not specified |
| 358 | Shojania | 2010 | Effect of point-of-care computer reminders on physician behaviour: a systematic review | Knowledge product(s) not specified |
| 359 | Smith | 2014 | Contextual Frameworks for Research on the Implementation of Complex System Interventions | Wrong design |
| 360 | Smithson | 2015 | Standardized patients in pharmacy education: An integrative literature review | Wrong design |
| 361 | Smits | 2002 | Problem based learning in continuing medical education: a review of controlled evaluation studies | Knowledge product(s) not specified |
| 362 | Solberg | 2000 | Guideline implementation: what the literature doesn't tell us | Wrong design |
| 363 | Thompson | 2011 | Do educational interventions improve nurses' clinical decision making and judgement? A systematic review | No implementation outcomes |
| 364 | Tooher | 2005 | A systematic review of strategies to improve prophylaxis for venous thromboembolism in hospitals | Secondary setting |
| 365 | Trogrlic | 2015 | A systematic review of implementation strategies for assessment, prevention, and management of ICU delirium and their effect on clinical outcomes | Secondary setting |
| 366 | van Boeijen | 2005 | Efficacy of self-help manuals for anxiety disorders in primary care: a systematic review | Exclusively patients population or general/public/organizational level |
| 367 | Wensing | 1994 | Single and combined strategies for implementing changes in primary care: a literature review | Wrong design |
| 368 | Zwarenstein | 2009 | Interprofessional collaboration: effects of practice-based interventions on professional practice and healthcare outcomes | Knowledge product(s) not specified |
| 369 | Albrecht | 2016 | Systematic Review of Knowledge Translation Strategies to Promote Research Uptake in Child Health Settings | Other : This reference was “Already included”. We found it in “references of another included review”. |
| 370 | Baker | 2015 | Tailored interventions to address determinants of practice | Other : This reference was “Already included”. We found it in “references of another included review”. |
| 371 | Chaillet | 2006 | Evidence-based strategies for implementing guidelines in obstetrics - A systematic review | Other : This reference was “Already included”. We found it in “references of another included review”. |
| 372 | Flodgren | 2016 | Tools developed and disseminated by guideline producers to promote the uptake of their guidelines | Other : This reference was “Already included”. We found it in “references of another included review”. |
| 373 | Gagnon | 2009 | Interventions for promoting information and communication technologies adoption in healthcare professionals | Other : This reference was “Already included”. We found it in “references of another included review”. |
| 374 | Hoomans | 2007 | The methodological quality of economic evaluations of guideline implementation into clinical practice: A systematic review of empiric studies | Other : This reference was “Already included”. We found it in “references of another included review”. |
| 375 | Ista | 2013 | Do implementation strategies increase adherence to pain assessment in hospitals? A systematic review | Other : This reference was “Already included”. We found it in “references of another included review”. |
| 376 | Jones | 2015 | Translating knowledge in rehabilitation: systematic review | Other : This reference was “Already included”. We found it in “references of another included review”. |
| 377 | Legare | 2012 | Patients' Perceptions of Sharing in Decisions A Systematic Review of Interventions to Enhance Shared Decision Making in Routine Clinical Practice | Other : This reference was “Already included”. We found it in “references of another included review”. |
| 378 | Perrier | 2011 | Interventions Encouraging the Use of Systematic Reviews in Clinical Decision-Making: A Systematic Review | Other : This reference was “Already included”. We found it in “references of another included review”. |
| 379 | Scott | 2012 | Systematic review of knowledge translation strategies in the allied health professions | Other : This reference was “Already included”. We found it in “references of another included review”. |
| 380 | Thompson | 2007 | Interventions aimed at increasing research use in nursing: a systematic review | Other : This reference was “Already included”. We found it in “references of another included review”. |
| 381 | van der Wees | 2008 | Multifaceted strategies may increase implementation of physiotherapy clinical guidelines: a systematic review | Other : This reference was “Already included”. We found it in “references of another included review”. |
| 382 | Watkins | 2015 | Effectiveness of implementation strategies for clinical guidelines to community pharmacy: a systematic review | Other : This reference was “Already included”. We found it in “references of another included review”. |
