# Supplementary material for: Implementation Strategies for Knowledge Products in Primary Health Care: Systematic Review of Systematic Reviews
Source: Interact J Med Res. 2022 Jul 11;11(2):e38419. doi: 10.2196/38419 (PMC9315889; doi:10.2196/38419)
Supplement: Multimedia Appendix 4 [file ijmr_v11i2e38419_app4.doc]

**General characteristics of included reviews (N=81)**

| **№** | **Author and Year** | **Title** | **Objectives of the review** | **Health domain** | **Population** | **Designs of primary studies** | **AMSTAR score** |
| --- | --- | --- | --- | --- | --- | --- | --- |
| **1** | **Abdullah 2014 [40]** | Measuring the Effectiveness of Mentoring as a Knowledge Translation Intervention for Implementing Empirical Evidence: A Systematic Review | To determine the effectiveness of mentoring as a KT intervention designed to increase the use of empirical evidence by healthcare professionals in clinical practice. | Primary and secondary healthcare services | HCP | RCT  nRCT  Before and After (without control) | Moderate |
| **2** | **Al Zoubi 2018 [41]** | The effectiveness of interventions designed to increase the uptake of clinical practice guidelines and best practices among musculoskeletal professionals: a systematic review | To summarize and evaluate evidence about the effectiveness of KT interventions to improve the uptake and application of clinical practice guidelines and best practices for musculoskeletal (MSK) disorders among MSK professionals. | Primary and secondary healthcare services | HCP and patients | RCT | Low |
| **3** | **Albrecht 2016 [42]** | Systematic Review of Knowledge Translation Strategies to Promote Research Uptake in Child Health Settings | To identify and synthesize the evidence on interventions aimed at putting research into child health settings. | Primary and secondary healthcare services | HCP | RCT  nRCT  Other | Critically low |
| **4** | **Bacci 2019 [43]** | Community pharmacist patient care services: A systematic review of approaches used for implementation and evaluation | To describe: (1) implementation strategies, and (2) dissemination and implementation (D&I) outcomes used to evaluate patient care services in community pharmacies. | Primary healthcare services | HCP and patients | Specific designs not reported | Critically low |
| **5** | **Baker 2015 [44]** | Tailored interventions to address determinants of practice (Review) | To determine whether tailored intervention strategies are effective in improving professional practice and healthcare outcomes. | Primary and secondary healthcare services | HCP | RCT | High |
| **6** | **Barwick 2012 [45]** | Knowledge translation efforts in child and youth mental health: a systematic review | To assess the current state of scientific knowledge regarding the effectiveness of KT strategies or interventions for CYMH. | Primary healthcare services | HCP | RCT  nRCT  ITS  Before and After (without control) | Critically low |
| **7** | **Boersma 2015 [46]** | The art of successful implementation of psychosocial interventions in residential dementia care: a systematic review of the literature based on the RE-AIM framework | To obtain insight into strategies for successful implementation of psychosocial interventions in the daily residential dementia care. | Primary healthcare services | HCP and patients | Other | Critically low |
| **8** | **Bright 2012 [47]** | Effect of Clinical Decision-Support Systems A Systematic Review | To evaluate the effect of CDSSs on clinical outcomes, health care processes, workload and efficiency, patient satisfaction, cost, and provider use and implementation. | Primary and secondary healthcare services | HCP and patients | RCT | Critically low |
| **9** | **Brusamento 2012 [48]** | Assessing the effectiveness of strategies to implement clinical guidelines for the management of chronic diseases at primary care level in EU Member States: A systematic review | To evaluate the effectiveness of strategies to implement clinical guidelines for chronic disease management in primary care in EU Member States. | Primary healthcare services | HCP and patients | RCT  nRCT  Before and After (without control) | Low |
| **10** | **Bywood 2009 [49]** | Effectiveness of opinion leaders for getting research into practice in the alcohol and other drugs field: Results from a systematic literature review | To evaluate the effectiveness of opinion leaders for specifically changing the work practices of their colleagues and to determine how this strategy may be used in the AOD context. | Primary and secondary healthcare services | HCP | RCT | Critically low |
| **11** | **Campbell 2019 [50]** | Knowledge Translation Strategies Used by Healthcare Professionals in Child Health Settings: An Updated Systematic Review | To provide an update on current evidence identifying KT interventions implementing research into child health settings | Primary healthcare services | HCP | RCT  nRCT | Critically low |
| **12** | **Chaillet 2006 [51]** | Evidence-based strategies for implementing guidelines in obstetrics - A systematic review | 1) to estimate which strategies are better adapted and most effective for implementing clinical practice guidelines in the specific field of obstetric care and 2) to identify specific barriers to change and facilitators in obstetric care. | Primary and secondary healthcare services | HCP and patients | RCT  nRCT  ITS | Critically low |
| **13** | **Ciliska 2005 [52]** | Diffusion and dissemination of evidence-based dietary strategies for the prevention of cancer | To determine what strategies have been evaluated to disseminate cancer control interventions that promote the uptake of adult healthy diet. | Primary healthcare services | HCP and patients | RCT  Cohort  Other | Critically low |
| **14** | **Colquhoun 2017 [53]** | A systematic review of interventions to increase the use of standardized outcome measures by rehabilitation professionals | To investigate the types and effectiveness of interventions studied that are aimed at increasing knowledge about, attitudes towards, and use of standardized outcome measures in the core rehabilitation professionals: Occupational Therapy, Physiotherapy, Speech Language Pathology. | Primary and secondary healthcare services | HCP | RCT  nRCT  Cohort  Before and After (without control) | Critically low |
| **15** | **Davis 1997 [54]** | Translating guidelines into practice. A systematic review of theoretic concepts, practical experience and research evidence in the adoption of clinical practice guidelines | To recommend effective strategies for implementing clinical practice guidelines (CPGs). | Primary and secondary healthcare services | HCP | Number and designs of primary studies not reported | Critically low |
| **16** | **De Angelis 2016 [55]** | Information and Communication Technologies for the Dissemination of Clinical Practice Guidelines to Health Professionals: A Systematic Review | To summarize the evidence pertaining to the use of ICTs for the dissemination of CPGs to health professionals. Specifically, with this review we sought to provide new knowledge on health professionals ‘perceived usability and change in practice behavior when using ICTs to disseminate CPGs. | Primary and secondary healthcare services | HCP | RCT  nRCT | Low |
| **17** | **Dexheimer 2008 [56]** | Prompting clinicians about preventive care measures: a systematic review of randomized controlled trials | To update the study by Balas et al.,17 which included 16 preventive care measures from the US Preventive Task Force, and to examine whether the amount of computerized reminder systems for preventive care have changed as clinicians increasingly utilize electronic health record systems when providing patient care. | Primary healthcare services | HCP and patients | RCT | Critically low |
| **18** | **Dexheimer 2014 [57]** | A systematic review of the implementation and impact of asthma protocols | To determine the most prevalent method of guideline implementation (paper, computer-generated, or computerized), as reported in the literature; establish which methods significantly improved clinical care; and identify the factors most commonly associated with a successful and sustainable asthma guideline implementation. | Primary and secondary healthcare services | HCP and patients | RCT  Before and After (without control) | Critically low |
| **19** | **Dwamena 2012 [58]** | Interventions for providers to promote a patient-centred approach in clinical consultations | To assess the effects of interventions for healthcare providers that aim to promote a patient-centred approach in clinical consultations. | Primary and secondary healthcare services | HCP and patients | RCT | High |
| **20** | **Espallargues 2000 [59]** | Provision of feedback on perceived health status to health care professionals - A systematic review of its impact | To assess the impact on the pro- cess and the outcomes of care of feeding back information on perceived health status to health care professionals in clinical practice. | Primary and secondary healthcare services | HCP and patients | RCT | Critically low |
| **21** | **Flodgren 2013 [60]** | Interventions to improve professional adherence to guidelines for prevention of device-related infections | To assess the effectiveness of different interventions, alone or in combination, which target healthcare professionals or healthcare organisations to improve professional adherence to infection control guidelines on device-related infection rates and measures of adherence. | Primary and secondary healthcare services | HCP | RCT  ITS | High |
| **22** | **Flodgren 2016 [61]** | Tools developed and disseminated by guideline producers to promote the uptake of their guidelines (Review) | To evaluate the effectiveness of implementation tools developed and disseminated by guideline producers, which accompany or follow the publication of a CPG, to promote uptake. A secondary objective is to determine which approaches to guideline implementation are most effective. | Primary and secondary healthcare services | HCP | RCT | High |
| **23** | **Flodgren 2017 [62]** | Interventions to change the behaviour of health professionals and the organisation of care to promote weight reduction in children and adults with overweight or obesity | To assess the effects of strategies to change the behaviour of health professionals or the organisation of care compared to standard care, to promote weight reduction in children and adults with overweight or obesity. | Primary and secondary healthcare services | HCP and patients | RCT  Other | High |
| **24** | **Flodgren 2019 [63]** | Local opinion leaders: effects on professional practice and healthcare outcomes (Review) | To assess the effectiveness of local opinion leaders in improving healthcare professionals' compliance with evidence-based practice and patient outcomes. | Primary and secondary healthcare services | HCP | RCT | High |
| **25** | **Forman-Hoffman 2017 [64]** | Quality improvement, implementation, and dissemination strategies to improve mental health care for children and adolescents: a systematic review | (1) assess the effectiveness of quality improvement, implementation, and dissemination strategies intended to improve the mental health care of children and adolescents;  (2) examine harms associated with these strategies; and  (3) determine whether effectiveness or harms differ for subgroups based on system, organizational, practitioner, or patient characteristics. | Primary and secondary healthcare services | HCP and patients | RCT  nRCT  ITS  Cohort | High |
| **26** | **Gagnon 2009 [65]** | Interventions for promoting information and communication technologies adoption in healthcare professionals (Review) | To assess the effectiveness of interventions to promote the adoption of ICT by healthcare professionals. | Primary and secondary healthcare services | HCP | RCT  ITS | High |
| **27** | **Gifford 2007 [66]** | Managerial Leadership for Nurses’ Use of Research Evidence: An Integrative Review of the Literature | (1) To describe leadership activities of nurse managers that influence nurses’ use of research evidence; and  (2) to identify interventions aimed at supporting nurse managers to influence research use in clinical nursing practice. | Primary and secondary healthcare services | HCP | RCT  Before and After (without control)  Other | Critically low |
| **28** | **Gould 2017 [67]** | Interventions to improve hand hygiene compliance in patient care | 1. To assess the short- and long-term success of strategies to improve hand hygiene compliance in patient care.  2. To determine whether a sustained increase in hand hygiene compliance can reduce rates of health care-associated infection. | Primary and secondary healthcare services | HCP | RCT  nRCT  ITS | High |
| **29** | **Goveia 2013 [68]** | Educational interventions to improve the meaningful use of Electronic Health Records: A review of the literature: BEME Guide No. 2 | To determine which educational interventions or what aspects of training are effective to improve meaningful use of the EHR in healthcare professionals. | Primary and secondary healthcare services | HCP | Cohort  Other | Critically low |
| **30** | **Gross 2001 [69]** | Implementing Practice Guidelines for Appropriate Antimicrobial Usage: A Systematic Review | To conduct a systematic review of guideline implementation studies for improving appropriate use of antimicrobial agents and to determine which implementation methods appear to improve the outcome of appropriate antimicrobial use. | Primary and secondary healthcare services | HCP and patients | RCT  nRCT  Cohort  Before and After (without control)  Other | Critically low |
| **31** | **Haggman-Laitila 2016 [70]** | A systematic review of the outcomes of educational interventions relevant to nurses with simultaneous strategies for guideline implementation | To systematically review the literature on the outcomes of educational interventions relevant to nurses with regard to guideline implementation. | Primary and secondary healthcare services | HCP | nRCT  Before and After (without control) | Critically low |
| **32** | **Hamade 2019 [71]** | Interventions to improve the use of EMRs in primary health care: a systematic review and meta-analysis | To review the literature to identify interventions and their effect on improving EMR use in primary healthcare settings | Primary healthcare services | HCP | RCT  nRCT  Cohort  Other | Critically low |
| **33** | **Heselmans 2009 [72]** | Effectiveness of electronic guideline-based implementation systems in ambulatory care settings - a systematic review | To systematically and comprehensively search the literature for studies evaluating the effectiveness of computer-based guideline implementation systems in ambulatory care settings with the multidimensionality of the guideline (the guideline needed to consist of several aspects or steps) and real-time interaction with the system during consultation as important inclusion criteria. | Primary and secondary healthcare services | HCP and patients | RCT  nRCT  ITS | Low |
| **34** | **Hoomans 2007 [73]** | The Methodological Quality of Economic Evaluations of Guideline Implementation into Clinical Practice: A Systematic Review of Empiric Studies | To assess advances in the economic evaluations of guideline implementation. | Primary and secondary healthcare services | HCP | RCT  nRCT  ITS | Critically low |
| **35** | **Imamura 2017 [74]** | A systematic review of implementation strategies to deliver guidelines on obstetric care practice in low-and middle-income countries | To perform a systematic review to assess whether such guideline implementation interventions improve maternal health care in LMICs. | Primary and secondary healthcare services | HCP | RCT  nRCT | Low |
| **36** | **Ince 2016 [75]** | A systematic review of the implementation of recommended psychological interventions for schizophrenia: Rates, barriers, and improvement strategies | To explore the implementation of the recommendations for psychological therapies by examining the published data regarding the rates of implementation of CBT and FI, and reviewing reported barriers against implementation, as well as strategies that have been attempted to improve implementation. | Primary and secondary healthcare services | HCP | RCT  Other | Critically low |
| **37** | **Ista 2013 [76]** | Do implementation strategies increase adherence to pain assessment in hospitals? A systematic review | To systematically review empirical evidence about the effectiveness of implementations strategies for the improvement of nurses ‘adherence to pain assessment recommendations in hospitalised patients. | Primary and secondary healthcare services | HCP | RCT  nRCT  ITS  Before and After (without control) | Critically low |
| **38** | **Jeffery 2015 [77]** | Interventions to improve adherence to cardiovascular disease guidelines: a systematic review | To synthesize the available research evidence about the effectiveness of interventions that target healthcare providers to improve adherence to CVD prevention and treatment guidelines and clinical outcomes. | Primary and secondary healthcare services | HCP and patients | RCT | Low |
| **39** | **Jensen 2016 [78]** | Systematic review of the cost-effectiveness of implementing guidelines on low back pain management in primary care: is transferability to other countries possible? | To identify, summarise and quality assess the available literature on the cost-effectiveness of implementing low back pain guidelines in primary care.  The secondary aim is to assess the transferability of the results to determine whether. | Primary healthcare services | HCP and patients | Other | Critically low |
| **40** | **Jones 2014 [79]** | Effectiveness of interventions to increase hepatitis C testing uptake among high-risk groups: a systematic review | To examine the effectiveness of interventions aimed at increasing uptake of case finding and testing among high-risk groups and health professionals involved in the promotion or provision of HCV testing. | Primary and secondary healthcare services | HCP and patients | RCT  nRCT | Low |
| **41** | **Jones 2015 [80]** | Translating Knowledge in Rehabilitation: Systematic Review | To identify, to assess, and to evaluate the effects of KT interventions on physical therapy, occupational therapy, and speech language pathology. | Primary and secondary healthcare services | HCP | RCT  nRCT  ITS  Before and After (without control)  Other | Low |
| **42** | **Kovacs 2018 [81]** | Systematic Review and Meta-analysis of the Effectiveness of Implementation Strategies for Non-communicable Disease Guidelines in Primary Health Care | To evaluate the effectiveness of interventions to improve guideline adherence of PCPs in the primary care setting. | Primary healthcare services | HCP | RCT  nRCT  Before and After (without control) | Critically low |
| **43** | **Légaré 2012 [82]** | Patients’ Perceptions of Sharing in Decisions A Systematic Review of Interventions to Enhance Shared Decision Making in Routine Clinical Practice | To evaluate patients ‘perceptions of the effectiveness of interventions to improve health professionals’ adoption of shared decision making in routine clinical practice. | Primary and secondary healthcare services | HCP | RCT | Critically low |
| **44** | **Lineker 2010 [83]** | Educational Interventions for Implementation of Arthritis Clinical Practice Guidelines in Primary Care: Effects on Health Professional Behavior | To provide a review and synthesis of studies evaluating the influence of educational programs designed to implement CPG for osteoarthritis (OA) and rheumatoid arthritis (RA) in primary care. | Primary healthcare services | HCP | RCT  nRCT | Critically low |
| **45** | **Luangasanatip 2015 [84]** | Comparative efficacy of interventions to promote hand hygiene in hospital: systematic review and network meta-analysis | To evaluate the relative efficacy of the World Health Organization 2005 campaign (WHO-5) and other interventions to promote hand hygiene among healthcare workers in hospital settings and to summarize associated information on use of resources. | Primary and secondary healthcare services | HCP | RCT  nRCT  ITS | Moderate |
| **46** | **Medves 2010 [85]** | Systematic review of practice guideline dissemination and implementation strategies for healthcare teams and team-based practice | To synthesis the literature relevant to guideline dissemination and implementation strategies for healthcare teams and team-based practice. | Primary and secondary healthcare services | HCP and patients | RCT  Cohort  Other | Critically low |
| **47** | **Menon 2009 [86]** | Strategies for rehabilitation professionals to move evidence-based knowledge into practice: a systematic review | To examine the effectiveness of single or multicomponent knowledge translation interventions for improving knowledge, attitudes, and practice behaviours of rehabilitation clinicians. | Primary healthcare services | HCP | RCT  Before and After (without control)  Other | Critically low |
| **48** | **Murthy 2012 [87]** | Interventions to improve the use of systematic reviews in decision-making by health system managers, policy makers and clinicians (Review) | To identify and assess the effects of information products based on the findings of systematic review evidence and organisational supports and processes designed to support the uptake of systematic review evidence by health system managers, policy makers and healthcare professionals. | Primary and secondary healthcare services | HCP | RCT  ITS | Moderate |
| **49** | **Nilsen 2006 [88]** | Effectiveness of strategies to implement brief alcohol intervention in primary healthcare A systematic review | To review systematically the available literature on implementation of brief alcohol interventions in primary healthcare in order to determine the effectiveness of the implementation efforts by the health care providers. | Primary healthcare services | HCP | RCT  nRCT | Critically low |
| **50** | **Noonan 2014 [89]** | Knowledge translation and implementation in spinal cord injury: a systematic review | To conduct a systematic review examining the effectiveness of knowledge translation (KT) interventions in changing clinical practice and patient outcomes. | Primary and secondary healthcare services | HCP and patients | Before and After (without control)  Other | Low |
| **51** | **Novins 2013 [90]** | Dissemination and implementation of evidence-based practices for child and adolescent mental health: a systematic review | To identify key findings from empirical studies examining the dissemination and implementation of EBPs for child and adolescent mental health. | Primary healthcare services | HCP and patients | RCT  nRCT  ITS  Before and After (without control)  Other | Critically low |
| **52** | **Okelo 2013 [91]** | Interventions to modify health care provider adherence to asthma guidelines: a systematic review | To assess the effect of interventions to improve health care providers’ adherence to asthma guidelines on health care process and clinical outcomes. | Primary healthcare services | HCP and patients | RCT  nRCT  Before and After (without control) | Critically low |
| **53** | **Ospina 2013 [92]** | A systematic review of the effectiveness of knowledge translation interventions for chronic noncancer pain management | To systematically locate and assess the evidence regarding the effectiveness of KT interventions for chronic noncancer pain management. | Primary and secondary healthcare services | HCP and patients | RCT  nRCT  Before and After (without control) | Moderate |
| **54** | **Pearson 2009 [93]** | Do computerised clinical decision support systems for prescribing change practice? A systematic review of the literature (1990-2007) | To examine the impact of CDSSs in targeting specific aspects of prescribing, namely initiating treatment (Before and After (without control) drug selection has taken place), monitoring patients on existing therapy and stopping treatment. | Primary and secondary healthcare services | HCP | RCT  nRCT  ITS | Low |
| **55** | **Perrier 2011 [94]** | Interventions Encouraging the Use of Systematic Reviews in Clinical Decision-Making: A Systematic Review | To systematically review the evidence on the impact of interventions for seeking, appraising, and applying evidence from systematic reviews in decision-making by clinicians. | Primary and secondary healthcare services | HCP | RCT | Moderate |
| **56** | **Perry 2011 [95]** | Effects of educational interventions on primary dementia care: A systematic review | To determine more precisely whether educational interventions for GPs and other primary care providers (PCPs) can be effective in improving their knowledge, attitudes and, most importantly, the quality of primary dementia care. | Primary healthcare services | HCP | RCT  Before and After (without control) | Critically low |
| **57** | **Pham 2019 [96]** | Strategies for implementing shared decision making in elective surgery by health care practitioners: A systematic review | To summarize relevant international scientific evidence on strategies aimed at facilitating or improving health care practitioners' adoption of SDM in elective surgery. The review evaluated the effectiveness of these strategies and described the characteristics of identified strategies. | Primary healthcare services | HCP and patients | RCT  nRCT | Critically low |
| **58** | **Powell 2014 [97]** | A Systematic Review of Strategies for Implementing Empirically Supported Mental Health Interventions | To characterize studies that test the effectiveness of implementation strategies in order to consolidate what has been learned about the effectiveness of implementation strategies and (perhaps more importantly) inform future research on the development, refinement, and testing of implementation strategies in mental health service settings. | Primary healthcare services | HCP and patients | RCT  nRCT | Critically low |
| **59** | **Rosen 2016 [98]** | A Review of Studies on the System-Wide Implementation of Evidence-Based Psychotherapies for Posttraumatic Stress Disorder in the Veterans Health Administration | (1) summarize recent research on adoption and sustainment of CPT and PE in the VHA system, using EPIS framework as the primary organizing framework, with a particular emphasis on ﬁndings related to variations in the inner context that occur in a system-wide implementation  (2) identify key lessons learned that can inform ongoing implementation efforts in large systems such as VHA, and  (3) inform the agenda for future research by identifying key aspects of EBP implementation and sustainment which have been under-researched in VHA. | Primary healthcare services | HCP and patients | RCT  Other | Critically low |
| **60** | **Scott 2012 [99]** | Systematic review of knowledge translation strategies in the allied health professions | 1. To systematically locate, assess, and report on studies from each respective allied health profession [1] that have investigated the effects of KT interventions;  2. To evaluate the interventions used to translate research into practice in terms of changes at the healthcare system, health provider, and/or patient level;  3. To describe how the interventions worked and the modifying variables relevant to the respective context (i.e., for whom does the intervention work, under what circumstances, and in what manner) [34];  4. To provide possible strategies to facilitate KT for allied healthcare professionals and decision makers responsible for policy and institution/unit protocols in healthcare settings;  5. To offer guidance for KT researchers in terms of the development of KT interventions for interprofessional healthcare teams. | Primary and secondary healthcare services | HCP | RCT  ITS  Cohort  Before and After (without control)  Other | Low |
| **61** | **Shanbhag 2018 [100]** | Effectiveness of implementation interventions in improving physician adherence to guideline recommendations in heart failure: a systematic review | The primary objective of our review was to examine the effectiveness of implementation interventions in increasing physician adherence to the specified HF guideline recommendations. Our secondary objectives were to assess the effect of implementation interventions on clinical outcomes, and to identify process and contextual factors that influence implementation success. | Primary and secondary healthcare services | HCP and patients | RCT  ITS  Cohort  Before and After (without control)  Other | Low |
| **62** | **SHIFFMAN 1999 [101]** | Computer-based Guideline Implementation Systems: A Systematic Review of Functionality and Effectiveness | To review the effectiveness of the computer-based interventions in influencing clinicians’ behavior and changing patient outcomes. | Primary and secondary healthcare services | HCP | RCT  nRCT  ITS | Critically low |
| **63** | **Siddiqui 2011 [102]** | The role of physician reminders in faecal occult blood testing for colorectal cancer screening | To examine whether the use of reminders to physicians increases the uptake of FOB testing | Primary healthcare services | HCP | RCT | Critically low |
| **64** | **Smeets 2007 [103]** | Effectiveness and costs of implementation strategies to reduce acid suppressive drug prescriptions: a systematic review | To evaluate the effectiveness of intervention methods for implementation of dyspepsia guidelines with the objective to reduce the volume and costs of ASD prescriptions. | Primary and secondary healthcare services | HCP and patients | RCT  Cohort | Critically low |
| **65** | **Smolders 2008 [104]** | Knowledge Transfer and Improvement of Primary and Ambulatory Care for Patients with Anxiety | To summarize current evidence on the effectiveness of knowledge transfer and interventions for improving the recognition and management of anxiety in primary and ambulatory mental health care; and to provide evidence-based advice and recommendations for mental health policy and practice. | Primary healthcare services | HCP and patients | RCT  Before and After (without control) | Critically low |
| **66** | **Soumerai 1989 [105]** | Improving drug prescribing in primary care - a critical analysis of the experimental literature | To review critically what is known about the effectiveness and efficiency of these approaches (printed educational materials, government warnings, prescription audits plus feedback, reminders at the time of prescribing, public-interest face-to-face "detailing”, etc) to improving prescribing practices in office settings, and to suggest the most promising methods for adoption and further research. | Primary healthcare services | HCP and patients | RCT  ITS  Before and After (without control)  Other | Critically low |
| **67** | **Souza 2011 [106]** | Computerized clinical decision support systems for primary preventive care: A decision-maker-researcher partnership systematic review of effects on process of care and patient outcomes | To review randomized controlled trials (RCTs) assessing the effects of CCDSSs for PPC on process of care, patient outcomes, harms, and costs. | Primary healthcare services | HCP and patients | RCT | Low |
| **68** | **Sunderji 2018 [107]** | Advancing Integrated Care through Psychiatric Workforce Development: A Systematic Review of Educational Interventions to Train Psychiatrists in Integrated Care | To identify all integrated care training experiences that have been evaluated in published and unpublished sources; examine their goals, contents, methods and outcomes; and distill, where possible, evidence informed recommendations for psychiatric training. | Primary and secondary healthcare services | HCP | Other | Low |
| **69** | **Thomas 1999b [108]** | Guidelines in professions allied to medicine (Review) | To identify and assess the effects of studies of the introduction of clinical practice guidelines in nursing (including health visiting), midwifery and other professions allied to medicine. | Primary and secondary healthcare services | HCP | RCT  nRCT  ITS | Moderate |
| **70** | **Thompson 2007 [109]** | Interventions aimed at increasing research use in nursing: a systematic review | To assess the evidence on interventions aimed explicitly at increasing research use in nursing practice. | Primary and secondary healthcare services | HCP | RCT  nRCT | Low |
| **71** | **Tudor Car 2019 [110]** | Health professions digital education on clinical practice guidelines: a systematic review by Digital Health Education collaboration | To evaluate the effectiveness of digital education in improving the adoption of clinical practice guidelines. | Primary and secondary healthcare services | HCP | RCT | Critically low |
| **72** | **Unverzagt 2014 [111]** | Strategies for guideline implementation in primary care focusing on patients with cardiovascular disease: a systematic review | ‘does the use of strategies for guideline implementation in primary care focusing on patients with CVD improve physician adherence and which strategy is the best? | Primary healthcare services | HCP and patients | RCT | Low |
| **73** | **van Steenkiste 2008 [112]** | Systematic review of implementation strategies for risk tables in the prevention of cardiovascular diseases | To systematically review the literature on health professionals’ performance with respect to cardiovascular risk tables, in order to search for implementation strategies that enable professionals to use cardiovascular risk tables effectively in the prevention of CVD. | Primary and secondary healthcare services | HCP | RCT  Cohort  Before and After (without control) | Critically low |
| **74** | **Watkins 2015 [113]** | Effectiveness of implementation strategies for clinical guidelines to community pharmacy: a systematic review | To describe the implementation strategies used, describe the resulting outcomes and to assess the effectiveness of the strategies | Primary healthcare services | HCP | RCT  nRCT | Low |
| **75** | **Wees 2008 [114]** | Multifaceted strategies may increase implementation of physiotherapy clinical guidelines: a systematic review | To assess the effectiveness of strategies to increase the implementation of physiotherapy clinical guidelines. | Primary and secondary healthcare services | HCP | RCT | Critically low |
| **76** | **Weinman 2007 [115]** | Effects of implementation of psychiatric guidelines on provider performance and patient outcome: systematic review | To summarize the evidence pertaining to benefits of mental health guidelines in the view of specific implementation strategies. | Primary and secondary healthcare services | HCP | RCT  nRCT  Before and After (without control) | Critically low |
| **77** | **Wensing 1998 [116]** | Implementing guidelines and innovations in general practice: which interventions are effective? | This review evaluates the effectiveness of interventions in influencing the implementation of guidelines and adoption of innovations in general practice | Primary and secondary healthcare services | HCP and patients | RCT  Before and After (without control) | Critically low |
| **78** | **Wilbur 2018 [117]** | Systematic Review of Standardized Patient Use in Continuing Medical Education | To determine the effectiveness of standardized patients (SP) in continuing medical education (CME) programs. | Primary and secondary healthcare services | HCP | RCT | Critically low |
| **79** | **Wilson 2016 [118]** | Knowledge translation studies in paediatric emergency medicine: A systematic review of the literature | To describe and assess the interventions used in emergency department settings | Primary and secondary healthcare services | HCP and patients | RCT  ITS  Before and After (without control)  Other | Critically low |
| **80** | **Wuchner 2014 [119]** | Integrative Review of Implementation Strategies for Translation of Research-Based Evidence by Nurses | To synthesize and critique experimental and/or quasi-experimental research that has evaluated implementation strategies for translation of research-based evidence into nursing practice in order to identify successful approaches for future use. | Primary and secondary healthcare services | HCP | RCT  Before and After (without control) | Critically low |
| **81** | **Zaher 2012 [120]** | Practice-based small group learning programs | To describe and evaluate the formats and content of and the effects on practice of practice-based small group learning (PBSGL) programs involving FPs. | Primary and secondary healthcare services | HCP | RCT  Other | Critically low |

HCP: healthcare providers. NA: not applicable. RCT: randomized controlled trials. nRCT; non-RCT. ITS: interrupted times series.
