# Supplementary material for: Implementation Strategies for Knowledge Products in Primary Health Care: Systematic Review of Systematic Reviews
Source: Interact J Med Res. 2022 Jul 11;11(2):e38419. doi: 10.2196/38419 (PMC9315889; doi:10.2196/38419)
Supplement: Multimedia Appendix 5 [file ijmr_v11i2e38419_app5.doc]

**Knowledge products, implementation strategies and outcomes measured in included reviews (N=81)**

| **№** | **Author and Year** | **Title** | **KP type** | **Single KP type** | **Implementation strategies**  **(EPOC categories)** | **Outcomes measured** |
| --- | --- | --- | --- | --- | --- | --- |
| **1** | **Abdullah 2014 [40]** | Measuring the Effectiveness of Mentoring as a Knowledge Translation Intervention for Implementing Empirical Evidence: A Systematic Review | Multiple | NA | Audit and feedback  Educational materials  Educational meetings  Educational outreach visits, or academic detailing  Local consensus processes  Local opinion leaders | - Conceptual knowledge use (i.e., practitioner’s knowledge): Knowledge - Conceptual knowledge use (i.e. practitioner’s attitudes/beliefs): Knowledge - Instrumental knowledge use (i.e., practitioner’s behavior or practice): Adoption - Practitioner outcomes: Acceptability |
| **2** | **Al Zoubi 2018 [41]** | The effectiveness of interventions designed to increase the uptake of clinical practice guidelines and best practices among musculoskeletal professionals: a systematic review | Single | Clinical guidelines | Educational materials  Educational meetings  Educational outreach visits, or academic detailing  Local opinion leaders  Reminders | - Knowledge about the guidelines: Knowledge - Self-confidence: Attitudes - Adherence to guidelines: Adoption - Change in clinical practice: Adoption - Clinician-patient communication: Adoption |
| **3** | **Albrecht 2016 [42]** | Systematic Review of Knowledge Translation Strategies to Promote Research Uptake in Child Health Settings | Multiple | NA | Audit and feedback  Educational materials  Educational meetings  Educational outreach visits, or academic detailing  Inter professional education  Reminders | - HCP professional/process outcomes (summary checklist scores on 3 simulated evaluation cases): Performance in a test situation - HCP/Process outcomes (attitudes about pain in children): Attitudes - HCP/Process outcomes (summary knowledge about pain in children): Knowledge - HCP/Process outcomes (# of pain assessments, analgesic administration) (improvement of pain mgmt techniques): Adoption - HCP/Process outcome (increased use of written asthma action plan) (mesure of improved asthma management): Adoption - HCP/Process outcomes (4 clinical skills related to medical decision making and consultations): Knowledge - HCP/Process outcomes (self-perceived competency on 5 comfort & knowledge/skill with process issues measures): Attitudes - HCP/Process outcomes (summary knowledge score): Knowledge - HCP/Process outcome (increase in clinic-specific proportions of adolescent girls screened for chlamydia trachomatis): Adoption - HCP/Process outcomes (increased percentage of 12–14-year olds receiving sealed secondary permanent molars): Adoption - HCP/Process outcome (increase chlamydia trachomatis screening rate): Adoption - HCP professional/process outcomes (Self-efficacy): Attitudes - HCP/Process outcomes (increased proportion of pediatricians who use computers and digital libraries digital libraries; increased # of times pediatricians use computers and digital libraries): Adoption - HCP/Process outcomes (4 factors related to intention to administer antipyretic agents to febrile children) (targeting inappropriate prescribing): Adoption - HCP/Process outcomes (overall guideline adherence; appropriateness of antibiotic prescription): Adoption - HCP/Process outcomes (proportion of patients with AOM receiving antibiotics prescription; proportion of patients receiving penicillin V; number of antibiotic prescriptions dispensed) (targeting overprescribing): Adoption - HCP/Process outcomes (4 self-reported domains of frequency & usefulness of child behavior management strategies; 5 observed categories of child behavior management skills): Appropriateness - Economic outcomes: Implementation Cost - HCP professional/process outcomes (Changes in communication & counseling behavior): Adoption - HCP professional/process outcomes (Measures of office visit length): Adoption - HCP/process outcome (reduced duration of therapy below typical 10-day course): Adoption - HCP/Process outcomes (proportion of prescriptions dispensed in accordance with evidence); Adoption - HCP/Process outcomes (2 measures of use of titration trials, systematic monitoring of medication treatment over time systematic monitoring of medication effectiveness): Adoption - HCP/Process outcomes (knowledge measures): Knowledge - HCP/Process outcomes (4 immunization compliance scores, 9 preventive care compliance scores, overall compliance score) (compliance with guidelines): Adoption |
| **4** | **Bacci 2019 [43]** | Community pharmacist patient care services: A systematic review of approaches used for implementation and evaluation | Multiple | NA | Tailored interventions | - Acceptability: Acceptability - Adoption: Adoption - Feasibility: Feasability - Cost: Implementation Cost - Appropriateness: Appropriateness - Fidelity: Fidelity - Sustainability: Sustainability - Penetration: Penetration |
| **5** | **Baker 2015 [44]** | Tailored interventions to address determinants of practice (Review) | Multiple | NA | Audit and feedback  Educational materials  Educational meetings  Educational outreach visits, or academic detailing  Local opinion leaders  Reminders  Tailored interventions | - Implementation of recommended practice (Nb of patients in 100 receiving recommended practice): Adoption |
| **6** | **Barwick 2012 [45]** | Knowledge translation efforts in child and youth mental health: a systematic review | Multiple | NA | Communities of practice  Educational materials  Educational meetings  Local opinion leaders | - Guideline implementation, practice change: Adoption - Knowledge: Knowledge |
| **7** | **Boersma 2015 [46]** | The art of successful implementation of psychosocial interventions in residential dementia care: a systematic review of the literature based on the RE-AIM framework | Single | Behavioural health intervention | Educational games  Educational materials  Educational meetings  Educational outreach visits, or academic detailing  Clinical practice guidelines  Inter professional education  Local opinion leaders  Managerial supervision  Tailored interventions | - RE-AIM: Reach (individual): Penetration - RE-AIM: Efficacy (individual): Fidelity - RE-AIM: Adoption (organization): Adoption - RE-AIM: Implementation (organization): Fidelity - RE-AIM: Maintenance (individual and organization): Sustainability |
| **8** | **Bright 2012 [47]** | Effect of Clinical Decision-Support Systems A Systematic Review | Single | Decision support tools | Feedback  Clinical practice guidelines  Reminders | - Economic outcomes: Cost: Implementation Cost - Economic outcomes: Cost-effectiveness: Implementation Cost - Use and implementation outcomes: Health care provider acceptance: Acceptability - Use and implementation outcomes: Health care provider satisfaction: Acceptability - Use and implementation outcomes: Health care provider use: Adoption - Use and implementation outcomes: Implementation: Fidelity - Relationship-centered: patient satisfaction: Acceptability |
| **9** | **Brusamento 2012 [48]** | Assessing the effectiveness of strategies to implement clinical guidelines for the management of chronic diseases at primary care level in EU Member States: A systematic review | Single | Clinical guidelines | Feedback  Educational materials  Educational outreach visits, or academic detailing  Reminders | - Process of care: Adoption |
| **10** | **Bywood 2009 [49]** | Effectiveness of opinion leaders for getting research into practice in the alcohol and other drugs field: Results from a systematic literature review | Multiple | NA | Continuous quality improvement  Educational materials  Educational meetings  Local opinion leaders | - Process outcomes to assess utilization of the innovation: Adoption |
| **11** | **Campbell 2019 [50]** | Knowledge Translation Strategies Used by Healthcare Professionals in Child Health Settings: An Updated Systematic Review | Multiple | NA | Audit and feedback  Educational materials  Educational meetings  Educational outreach visits, or academic detailing  Patient mediated interventions  Reminders | - Change at the professional/process: Adoption - Change at the patient level: Acceptability - Change at the economic level: Implementation Cost |
| **12** | **Chaillet 2006 [51]** | Evidence-based strategies for implementing guidelines in obstetrics - A systematic review | Single | Clinical guidelines | Audit and feedback  Continuous quality improvement  Educational materials  Educational outreach visits, or academic detailing  Local opinion leaders  Reminders  Tailored interventions | - Reducing overall caesarean section rates: Feasibility |
| **13** | **Ciliska 2005 [52]** | Diffusion and dissemination of evidence-based dietary strategies for the prevention of cancer | Single | Behavioural health intervention | Educational materials  Educational meetings  Educational outreach visits, or academic detailing  Local opinion leaders | - Numbers of training sessions conducted: Fidelity - Numbers of physicians trained: Penetration - Counts of peer-education strategies according to gender and ethnicity: Fidelity |
| **14** | **Colquhoun 2017 [53]** | A systematic review of interventions to increase the use of standardized outcome measures by rehabilitation professionals | Single | Decision support tools | Audit and feedback  Educational materials  Educational meetings  Clinical practice guidelines  Managerial supervision  Reminders | - Use of standardized outcomes mesure: Adoption - Attitudes towards standardized outcomes measure: Attitudes |
| **15** | **Davis 1997 [54]** | Translating guidelines into practice. A systematic review of theoretic concepts, practical experience and research evidence in the adoption of clinical practice guidelines | Single | Clinical guidelines | Audit and feedback  Educational materials  Educational outreach visits, or academic detailing  Local opinion leaders  Reminders | - Physician performance (adoption of clinical guidelines): Adoption |
| **16** | **De Angelis 2016 [55]** | Information and Communication Technologies for the Dissemination of Clinical Practice Guidelines to Health Professionals: A Systematic Review | Single | Clinical guidelines | Audit  Feedback  Audit and feedback  Educational games  Educational materials  Educational meetings  Educational outreach visits, or academic detailing  Reminders | - Usability: Appropriateness - Practice behavior: Knowledge |
| **17** | **Dexheimer 2008 [56]** | Prompting clinicians about preventive care measures: a systematic review of randomized controlled trials | Single | Management health intervention | Reminders | - Uptake of preventive care procedure: Adoption |
| **18** | **Dexheimer 2014 [57]** | A systematic review of the implementation and impact of asthma protocols | Single | Clinical guidelines | Patient mediated interventions  Reminders | - HCP performance: Compliance with provided guidelines or prescribing guidelines: Adoption |
| **19** | **Dwamena 2012 [58]** | Interventions for providers to promote a patient-centred approach in clinical consultations | Single | Behavioural health intervention | Educational materials  Educational meetings  Clinical practice guidelines  Reminders | - Consultation process: Adoption - Patient satisfaction: Satisfaction - Patient health behaviour: Acceptability |
| **20** | **Espallargues 2000 [59]** | Provision of feedback on perceived health status to health care professionals - A systematic review of its impact | Single | Management health intervention | Feedback  Educational meetings | - Process of care (use of health services, diagnostic, treatment: Adoption - Usefulness to clinicians: Appropriateness - Patient outcomes: Performance in a test situation |
| **21** | **Flodgren 2013 [60]** | Interventions to improve professional adherence to guidelines for prevention of device-related infections | Single | Management health intervention | Feedback  Audit and feedback  Educational materials  Educational meetings  Educational outreach visits, or academic detailing  Clinical practice guidelines  Local consensus processes  Local opinion leaders  Reminders | - Compliance with guideline for device-related infections: Adoption - Proportion/rate of invasive device-related infections: Performance in a test situation |
| **22** | **Flodgren 2016 [61]** | Tools developed and disseminated by guideline producers to promote the uptake of their guidelines (Review) | Multiple | NA | Educational materials  Educational meetings  Reminders  Tailored interventions | - Measure of healthcare professional adherence to guidelines: Adoption |
| **23** | **Flodgren 2017 [62]** | Interventions to change the behaviour of health professionals and the organisation of care to promote weight reduction in children and adults with overweight or obesity | Multiple | NA | Audit  Feedback  Audit and feedback  Monitoring the performance of the delivery of healthcare  Educational materials  Educational meetings  Clinical practice guidelines  Local consensus processes  Patient mediated interventions  Reminders  Tailored interventions | - Health practitioners’ satisfaction: Acceptability - Costs: Implementation Cost - Participant outcomes Satisfaction with provider practice or healthcare provision: Acceptability |
| **24** | **Flodgren 2019 [63]** | Local opinion leaders: effects on professional practice and healthcare outcomes (Review) | Multiple | NA | Feedback  Audit and feedback  Continuous quality improvement  Educational materials  Educational meetings  Educational outreach visits, or academic detailing  Local opinion leaders  Reminders | - Main comparison: Local opinion leaders alone or w. other intervention(s) compared w. no intervention, a single int., or the same single or more intervention(s) Compliance with evidence-based practice (Absolute improvement in compliance): Adoption - Secondary comparison 1: Local opinion leaders alone compared w. no intervention Compliance with evidence-based practice (Absolute improvement in compliance): Adoption - Secondary comparison 2: Local opinion leaders alone compared w. a single int.Compliance with evidence-based practice (Absolute improvement in compliance) : Adoption - Secondary comparison 3: Local opinion leaders w. a single or more other intervention(s) compared w. the same single or more intervention(s) Compliance with evidence-based practice (Absolute improvement in compliance): Adoption - Secondary comparison 4: Local opinion leaders w. a single or more other intervention(s) compared w. no intervention Compliance with evidence-based practice (Absolute improvement in compliance): Adoption |
| **25** | **Forman-Hoffman 2017 [64]** | Quality improvement, implementation, and dissemination strategies to improve mental health care for children and adolescents: a systematic review | Multiple | NA | Audit and feedback  Continuous quality improvement  Educational materials  Educational meetings  Educational outreach visits, or academic detailing  Inter professional education  Reminders  Routine patient reported outcome | - Implementation competence (competent delivery of all components of an EBP program): Fidelity - Improved adherence to EPB or guidelines: Adoption - Improved morale, engagement and stress: Attitudes |
| **26** | **Gagnon 2009 [65]** | Interventions for promoting information and communication technologies adoption in healthcare professionals (Review) | Single | Technological health intervention | Feedback  Audit and feedback  Educational materials  Educational meetings  Educational outreach visits, or academic detailing | - Objective measures of the adoption or use of the ICT application by healthcare professionals: Adoption - Objective measure of general clinical performance or process outcome (e.g. number of tests ordered or decision to prescribe a particular drug): Adoption - Healthcare professionals’ knowledge: Knowledge - Healthcare professionals’ attitudes: Attitudes - Healthcare professionals’ satisfaction: Satisfaction |
| **27** | **Gifford 2007 [66]** | Managerial Leadership for Nurses’ Use of Research Evidence: An Integrative Review of the Literature | Multiple | NA | Audit  Educational games  Educational materials  Educational meetings  Clinical practice guidelines  Inter professional education  Local consensus processes  Local opinion leaders  Managerial supervision  Reminders | - Process outcomes: Adoption - Research use: Adoption evidence-based practice: Adoption |
| **28** | **Gould 2017 [67]** | Interventions to improve hand hygiene compliance in patient care | Single | Behavioural health intervention | Feedback  Reminders | - Compliance with handwashing: Adoption - Compliance to recommendations for hand hygiene: Adoption |
| **29** | **Goveia 2013 [68]** | Educational interventions to improve the meaningful use of Electronic Health Records: A review of the literature: BEME Guide No. 2 | Single | Technological health intervention | Feedback  Clinical incident reporting  Educational meetings | - Use of EHR: Adoption - Participant satisfaction: Satisfaction |
| **30** | **Gross 2001 [69]** | Implementing Practice Guidelines for Appropriate Antimicrobial Usage: A Systematic Review | Single | Clinical guidelines | Feedback  Monitoring the performance of the delivery of healthcare  Educational materials  Educational meetings  Educational outreach visits, or academic detailing  Local consensus processes  Local opinion leaders  Reminders | - Appropriate antimicrobial use: Adoption |
| **31** | **Haggman-Laitila 2016 [70]** | A systematic review of the outcomes of educational interventions relevant to nurses with simultaneous strategies for guideline implementation | Single | Clinical guidelines | Audit  Feedback  Audit and feedback  Educational games  Educational materials  Educational meetings  Clinical practice guidelines  Inter professional education  Local consensus processes  Reminders | - Positive changes in nurses’ attitudes: Attitudes - Improvement in nurses’knowledge base: Knowledge - Nurses’ enhanced confidence in using guidelines and changing practices: Acceptability - Self-reported improvement in nurses’evidence-based decision making and care practices based on guidelines: Adoption - Improvement in nurses’ quality of care: Adoption |
| **32** | **Hamade 2019 [71]** | Interventions to improve the use of EMRs in primary health care: a systematic review and meta-analysis | Single | Management health intervention | Feedback  Monitoring the performance of the delivery of healthcare  Continuous quality improvement  Educational materials  Educational meetings  Educational outreach visits, or academic detailing  Clinical practice guidelines  Reminders | - Use of EMR functions: Adoption |
| **33** | **Heselmans 2009 [72]** | Effectiveness of electronic guideline-based implementation systems in ambulatory care settings - a systematic review | Single | Clinical guidelines | Feedback | - Physician adherence or compliance to CPGs: Adoption |
| **34** | **Hoomans 2007 [73]** | The Methodological Quality of Economic Evaluations of Guideline Implementation into Clinical Practice: A Systematic Review of Empiric Studies | Single | Clinical guidelines | Audit and feedback  Educational materials  Educational meetings  Educational outreach visits, or academic detailing  Clinical practice guidelines  Local consensus processes  Local opinion leaders  Reminders | - Costs of guideline implementation: Implementation Cost - Health-care professional outcomes (to change the behavior of health-care professionals): Adoption |
| **35** | **Imamura 2017 [74]** | A systematic review of implementation strategies to deliver guidelines on obstetric care practice in low-and middle-income countries | Single | Clinical guidelines | Audit and feedback  Educational materials  Educational meetings  Educational outreach visits, or academic detailing  Local consensus processes  Local opinion leaders | - Uptake of recommended obstétric care pratice: Adoption - Management of obstetric emergencies: Adoption |
| **36** | **Ince 2016 [75]** | A systematic review of the implementation of recommended psychological interventions for schizophrenia: Rates, barriers, and improvement strategies | Single | Clinical guidelines | Educational materials  Educational meetings  Educational outreach visits, or academic detailing  Local consensus processes  Local opinion leaders  Managerial supervision | - Rates of implementation for cognitive behavioural therapy (CBT) and family intervention (FI): Adoption |
| **37** | **Ista 2013 [76]** | Do implementation strategies increase adherence to pain assessment in hospitals? A systematic review | Single | Clinical guidelines | Audit  Feedback  Audit and feedback  Educational materials  Educational meetings  Educational outreach visits, or academic detailing  Local consensus processes  Local opinion leaders  Reminders  Tailored interventions | - Adherence rates (percentages) for all types of pain assessment (e.g. Assessment, reassessment after treatment intervention): Adoption |
| **38** | **Jeffery 2015 [77]** | Interventions to improve adherence to cardiovascular disease guidelines: a systematic review | Single | Clinical guidelines | Audit  Feedback  Audit and feedback  Monitoring the performance of the delivery of healthcare  Continuous quality improvement  Educational materials  Educational meetings  Educational outreach visits, or academic detailing  Clinical practice guidelines  Inter professional education  Local opinion leaders  Reminders  Tailored interventions | - Adherence to CVD guidelines Follow-up: 6–24 months: Adoption - Adherence to CVD guidelines Follow-up: 7–27 months: Adoption - Adherence to CVD guidelines Follow-up: median 6 months: Adoption |
| **39** | **Jensen 2016 [78]** | Systematic review of the cost-effectiveness of implementing guidelines on low back pain management in primary care: is transferability to other countries possible? | Single | Clinical guidelines | Feedback  Educational meetings  Clinical practice guidelines | - Cost effectiveness of implementing guidelines: Implementation Cost |
| **40** | **Jones 2014 [79]** | Effectiveness of interventions to increase hepatitis C testing uptake among high-risk groups: a systematic review | Single | Technological health intervention | Educational materials  Clinical practice guidelines | - Uptake of hepatitis C virus testing: Adoption |
| **41** | **Jones 2015 [80]** | Translating Knowledge in Rehabilitation: Systematic Review | Multiple | NA | Audit  Audit and feedback  Educational materials  Educational meetings  Educational outreach visits, or academic detailing  Local consensus processes  Local opinion leaders  Reminders | - Professional/process: Adoption |
| **42** | **Kovacs 2018 [81]** | Systematic Review and Meta-analysis of the Effectiveness of Implementation Strategies for Non-communicable Disease Guidelines in Primary Health Care | Single | Clinical guidelines | Audit  Feedback  Audit and feedback  Educational materials  Educational meetings  Educational outreach visits, or academic detailing  Local opinion leaders  Patient mediated interventions  Reminders | - Prescription: Adoption - Diagnostic behaviour: Adoption - Patient counselling: Adoption - Knowledge: Knowledge |
| **43** | **Légaré 2012 [82]** | Patients’ Perceptions of Sharing in Decisions A Systematic Review of Interventions to Enhance Shared Decision Making in Routine Clinical Practice | Single | Behavioural health intervention | Audit and feedback  Educational meetings  Educational outreach visits, or academic detailing  Patient mediated interventions | - Health professionals’ adoption of shared decision making: Adoption |
| **44** | **Lineker 2010 [83]** | Educational Interventions for Implementation of Arthritis Clinical Practice Guidelines in Primary Care: Effects on Health Professional Behavior | Single | Clinical guidelines | Feedback  Audit and feedback  Educational materials  Educational meetings  Educational outreach visits, or academic detailing  Reminders | - Behavioral outcomes that ensured actual knowledge utilization: Adoption |
| **45** | **Luangasanatip 2015 [84]** | Comparative efficacy of interventions to promote hand hygiene in hospital: systematic review and network meta-analysis | Single | Behavioural health intervention | Feedback  Educational materials  Educational meetings | - Compliance with hand hygiene: Adoption |
| **46** | **Medves 2010 [85]** | Systematic review of practice guideline dissemination and implementation strategies for healthcare teams and team-based practice | Multiple | NA | Audit and feedback  Educational materials  Educational meetings  Educational outreach visits, or academic detailing  Clinical practice guidelines  Local consensus processes  Local opinion leaders  Managerial supervision  Patient mediated interventions  Reminders | - Economic outcomes: Implementation Cost |
| **47** | **Menon 2009 [86]** | Strategies for rehabilitation professionals to move evidence-based knowledge into practice: a systematic review | Single | Management health intervention | Feedback  Educational materials  Educational meetings  Educational outreach visits, or academic detailing  Clinical practice guidelines  Local opinion leaders  Reminders | - HCP attitudes towards EBP: Attitudes - HCP knowledge: Knowledge - Practice behaviours: Adoption |
| **48** | **Murthy 2012 [87]** | Interventions to improve the use of systematic reviews in decision-making by health system managers, policy makers and clinicians (Review) | Multiple | NA | Audit and feedback  Educational materials  Educational meetings  Educational outreach visits, or academic detailing | - Utilisation of research (Compliance with EBP for obstetrical practices): Adoption - Utilisation of research (Rates of surgery for glue ear): Adoption - Utilisation of research (SSRI prescription): Adoption - Utilisation of research (access to Cochrane Pregnancy and Childbirth Reviews and a short video): Adoption - Utilisation of research (planning a programme related to healthy body weight promotion): Adoption - Utilisation of healthcare resources: costs: Implementation Cost - Knowledge: Knowledge - Perceived understanding and ease of use: Satisfaction - Preferences and attitudes: Attitudes |
| **49** | **Nilsen 2006 [88]** | Effectiveness of strategies to implement brief alcohol intervention in primary healthcare A systematic review | Single | Behavioural health intervention | Audit  Educational materials  Educational meetings  Educational outreach visits, or academic detailing  Managerial supervision  Reminders  Tailored interventions | - Material utilization rate: Adoption - Screening rate: Adoption - Brief alcohol intervention rate: Adoption |
| **50** | **Noonan 2014 [89]** | Knowledge translation and implementation in spinal cord injury: a systematic review | Multiple | NA | Audit  Feedback  Educational materials  Educational meetings  Clinical practice guidelines  Inter professional education  Local opinion leaders  Reminders | - Clinician behavior change: Acceptability |
| **51** | **Novins 2013 [90]** | Dissemination and implementation of evidence-based practices for child and adolescent mental health: a systematic review | Multiple | NA | Feedback  Monitoring the performance of the delivery of healthcare  Communities of practice  Clinical practice guidelines | - NR: Fidelity - NR: Implementation Cost - NR: Adoption |
| **52** | **Okelo 2013 [91]** | Interventions to modify health care provider adherence to asthma guidelines: a systematic review | Single | Clinical guidelines | Audit  Feedback  Audit and feedback  Continuous quality improvement  Educational materials  Educational meetings  Local consensus processes  Local opinion leaders  Reminders  Tailored interventions | - Health care process outcomes (prescription for controller medecines + self-management education/asthma action plan): Adoption - Clinical outcomes (ED visit/hospitalization + Missed days of school/work): Performance in a test situation |
| **53** | **Ospina 2013 [92]** | A systematic review of the effectiveness of knowledge translation interventions for chronic noncancer pain management | Single | Management health intervention | Audit and feedback  Educational materials  Educational meetings  Educational outreach visits, or academic detailing  Local opinion leaders  Reminders | - Change in Non-cancer chronic pain management practice: Adoption - HCP Knowledge: Knowledge - Clinical outcomes: Performance in a test situation - Patient Knowledge: Knowledge |
| **54** | **Pearson 2009 [93]** | Do computerised clinical decision support systems for prescribing change practice? A systematic review of the literature (1990-2007) | Single | Decision support tools | Audit and feedback | - Prescribing change pratices (initiating, monitoring and stopping therapy): Adoption |
| **55** | **Perrier 2011 [94]** | Interventions Encouraging the Use of Systematic Reviews in Clinical Decision-Making: A Systematic Review | Single | Behavioural health intervention | Audit and feedback  Educational materials  Educational meetings  Educational outreach visits, or academic detailing  Local opinion leaders  Reminders | - Professional performance (e.g., prescribing patterns, use of diagnostic tests): Adoption - Healthcare providers’ satisfaction: Satisfaction - Healthcare providers’ knowledge: Knowledge - Healthcare providers’ attitudes: Attitudes |
| **56** | **Perry 2011 [95]** | Effects of educational interventions on primary dementia care: A systematic review | Single | Management health intervention | Educational materials  Educational meetings  Educational outreach visits, or academic detailing  Reminders | - Health care outcome: Performance in a test situation - Health professional’s behaviour, performance or practice: Adoption - Learning and knowledge: Knowledge - Attitudes: Attitudes |
| **57** | **Pham 2019 [96]** | Strategies for implementing shared decision making in elective surgery by health care practitioners: A systematic review | Single | Management health intervention | Feedback  Educational materials  Educational meetings  Local opinion leaders  Reminders | - The review included studies that reported measures of decision processes and decision outcome (eg, decision quality), measures that have been highlighted by the international Patient Decision Aids Standards group as vital in SDM.16 Studies that examined both decision process and decision outcomes were included, as were those that measured decision process only: Acceptability - The review included studies that reported measures of decision processes and decision outcome (eg, decision quality), measures that have been highlighted by the international Patient Decision Aids Standards group as vital in SDM.16 Studies that examined both decision process and decision outcomes were included, as were those that measured decision process only: Acceptability |
| **58** | **Powell 2014 [97]** | A Systematic Review of Strategies for Implementing Empirically Supported Mental Health Interventions | Multiple | NA | Feedback  Audit and feedback  Educational materials  Educational meetings  Educational outreach visits, or academic detailing  Clinical practice guidelines  Local opinion leaders  Reminders  Tailored interventions | - Implementation outcomes: Acceptability: Acceptability - Implementation outcomes: Cost: Implementation Cost - Implementation outcomes: Fidelity: Fidelity - Implementation outcomes: Penetration: Penetration - Implementation outcomes: Sustainability: Sustainability - Implementation outcomes: Uptake: Adoption - Implementation outcomes: Feasability: Feasability - Client outcome: Satisfaction: Acceptability |
| **59** | **Rosen 2016 [98]** | A Review of Studies on the System-Wide Implementation of Evidence-Based Psychotherapies for Posttraumatic Stress Disorder in the Veterans Health Administration | Single | Behavioural health intervention | Educational meetings | - Implementation Outcomes: Reach (RE-AIM): Penetration - Implementation Outcomes: Effectiveness: Adoption |
| **60** | **Scott 2012 [99]** | Systematic review of knowledge translation strategies in the allied health professions | Multiple | NA | Audit and feedback  Educational meetings  Educational outreach visits, or academic detailing  Local opinion leaders  Reminders | - Change at the professional/process level: Adoption - Change at the economic level: Implementation Cost |
| **61** | **Shanbhag 2018 [100]** | Effectiveness of implementation interventions in improving physician adherence to guideline recommendations in heart failure: a systematic review | Single | Clinical guidelines | Audit and feedback  Educational materials  Educational meetings  Educational outreach visits, or academic detailing  Inter professional education  Reminders | - Process outcomes: measures that assess guideline-consistent activities undertaken by a provider: Adoption |
| **62** | **SHIFFMAN 1999 [101]** | Computer-based Guideline Implementation Systems: A Systematic Review of Functionality and Effectiveness | Single | Clinical guidelines | Reminders | - Improvement in provider documentation: Adoption - Provider adherence to the guidelines: Adoption - Clinician satisfaction: Satisfaction |
| **63** | **Siddiqui 2011 [102]** | The role of physician reminders in faecal occult blood testing for colorectal cancer screening | Single | Management health intervention | Reminders | - Uptake of FOB testingAdoption |
| **64** | **Smeets 2007 [103]** | Effectiveness and costs of implementation strategies to reduce acid suppressive drug prescriptions: a systematic review | Single | Pharmacological health intervention | Feedback  Educational materials  Educational meetings  Educational outreach visits, or academic detailing  Clinical practice guidelines  Local consensus processes  Patient mediated interventions | - Number of prescription and diagnostic tests: Adoption |
| **65** | **Smolders 2008 [104]** | Knowledge Transfer and Improvement of Primary and Ambulatory Care for Patients with Anxiety | Single | Management health intervention | Feedback  Audit and feedback  Educational meetings  Educational outreach visits, or academic detailing  Local consensus processes | - Clinical processAdoption use of ressources: Adoption |
| **66** | **Soumerai 1989 [105]** | Improving drug prescribing in primary care - a critical analysis of the experimental literature | Multiple | NA | Audit  Feedback  Educational materials  Educational outreach visits, or academic detailing  Local opinion leaders  Reminders | - Improving physician drug prescribing: Adoption - Knowledge: Knowledge |
| **67** | **Souza 2011 [106]** | Computerized clinical decision support systems for primary preventive care: A decision-maker-researcher partnership systematic review of effects on process of care and patient outcomes | Single | Management health intervention | Audit and feedback  Reminders | - Process of care: Adoption - Patient outcomes: Performance in a test situation - Costs of developing, implementing, and maintaining a CCDSS: Implementation Cost |
| **68** | **Sunderji 2018 [107]** | Advancing Integrated Care through Psychiatric Workforce Development: A Systematic Review of Educational Interventions to Train Psychiatrists in Integrated Care | Single | Management health intervention | Continuous quality improvement  Educational meetings | - Buy-in to integrated care among diverse stakeholders: Acceptability - Leaner experience: Satisfaction - Learner awareness,Knowlegde: Knowledge - Learner skill, developpement: Adoption - Communication and coordination of care: Adoption |
| **69** | **Thomas 1999b [108]** | Guidelines in professions allied to medicine (Review) | Single | Clinical guidelines | Educational materials  Educational outreach visits, or academic detailing  Clinical practice guidelines  Local consensus processes  Local opinion leaders | - Staff satisfaction: Acceptability - Changes in processes of care: Adoption - Economic outcomes: Costs: Implementation Cost |
| **70** | **Thompson 2007 [109]** | Interventions aimed at increasing research use in nursing: a systematic review | Multiple | NA | Educational meetings  Local opinion leaders | - Research utilization: Adoption |
| **71** | **Tudor Car 2019 [110]** | Health professions digital education on clinical practice guidelines: a systematic review by Digital Health Education collaboration | Single | Clinical guidelines | Educational materials | - Knowledge: Knowledge - Skill: Performance in a test situation - Satisfaction: Satisfaction - Change in professional practice or behaviour (Change in inappropriate screening ; quality of practice ; proportion of patients screened & treated ; proportion of tests, examinations, & treatments compliant w. guidelines ; proportion of patients who received all/ most/ at least one intervention per guideline ; proportion of patients screened & rate of medication intensification for HbA1c, BP and LDL ; rate of medication intensification and lifestyle counseling ; self-reported practice change) : Adoption |
| **72** | **Unverzagt 2014 [111]** | Strategies for guideline implementation in primary care focusing on patients with cardiovascular disease: a systematic review | Single | Clinical guidelines | Audit and feedback  Educational meetings  Patient mediated interventions  Reminders | - Physician adherence to guideline: Adoption |
| **73** | **van Steenkiste 2008 [112]** | Systematic review of implementation strategies for risk tables in the prevention of cardiovascular diseases | Single | Behavioural health intervention | Audit and feedback  Educational materials  Educational meetings  Educational outreach visits, or academic detailing  Patient mediated interventions  Reminders | - Use of cardiovascular risk tables: Adoption |
| **74** | **Watkins 2015 [113]** | Effectiveness of implementation strategies for clinical guidelines to community pharmacy: a systematic review | Single | Clinical guidelines | Audit and feedback  Educational materials  Educational meetings  Educational outreach visits, or academic detailing  Clinical practice guidelines  Reminders  Tailored interventions | - Economic outcomes: Implementation Cost - Practitioner/process outcome: adherence to recommended practice or guidelines: Fidelity - Satisfaction of both patients and practitioners: Acceptability |
| **75** | **Wees 2008 [114]** | Multifaceted strategies may increase implementation of physiotherapy clinical guidelines: a systematic review | Single | Clinical guidelines | Feedback  Educational games  Educational materials  Educational meetings  Educational outreach visits, or academic detailing  Clinical practice guidelines  Local opinion leaders  Reminders | - Change in professional practice: Adoption - Reduce cost of care: Implementation Cost |
| **76** | **Weinman 2007 [115]** | Effects of implementation of psychiatric guidelines on provider performance and patient outcome: systematic review | Single | Clinical guidelines | Audit and feedback  Continuous quality improvement  Educational materials  Educational outreach visits, or academic detailing  Patient mediated interventions  Reminders  Tailored interventions | - Detection rates of comorbid and medical disorders; probability of receiveing medication, psychotherapy or combined treatment or combination treatment: Adoption - Smoking rate assessment, smoking cessation counselling and documentation rate, nb of smoking cessation prescription: Adoption - Concordance with guidelines: Adoption - Adherence to guideline recommendations: Adoption - Tobacco use diagnostic codes three months BA the intervention as marker for guideline compliance: Adoption - Frequency and dose of antipsychotic medication prescribed: Adoption - Frequency and dose of antipsychotic medication prescribed: Adoption - Depression recognition rates: Adoption - Lab test rate: Adoption - Diagnosis of depression, proportion of patients taking antidepressants, psychiatrist referrals: Adoption - Rate of suicide risk assessment; proportion of patients with Beck Depression Inventory Score: Adoption - Antidepressant dose and duration of treatment: Adoption - Knowledge: Knowledge - Attitudes: Acceptability - Self-reported practice: Adoption - Medication changes, frequency and type of antipsychotic polypharmacy: Adoption - Rate of thyroid function tests and creatinine checks: Adoption - Prescribing patterns, control rates, depression recognition, prescribing and symptomatology: Adoption |
| **77** | **Wensing 1998 [116]** | Implementing guidelines and innovations in general practice: which interventions are effective? | Single | Management health intervention | Feedback  Audit and feedback  Educational materials  Patient mediated interventions  Reminders | - Compliance with protocol: Adoption |
| **78** | **Wilbur 2018 [117]** | Systematic Review of Standardized Patient Use in Continuing Medical Education | Multiple | NA |  | - Acquired knowledge: Knowledge - Performance with standard patients: Performance in a test situation - Behavior change: use of taught smoking cessation communication processes; opioid prescribing rates: Adoption |
| **79** | **Wilson 2016 [118]** | Knowledge translation studies in paediatric emergency medicine: A systematic review of the literature | Multiple | NA | Audit  Feedback  Audit and feedback  Monitoring the performance of the delivery of healthcare  Educational materials  Educational meetings  Clinical practice guidelines  Local consensus processes  Local opinion leaders  Reminders  Tailored interventions | - RE-AIM-Reach: Penetration - RE-AIM-adoption: Adoption - RE-AIM-implementation: Fidelity - RE-AIM-maintenance: Sustainability - ED physician satisfaction: Acceptability - Patient satisfaction: Acceptability - Compliance with guideline: Appropriateness |
| **80** | **Wuchner 2014 [119]** | Integrative Review of Implementation Strategies for Translation of Research-Based Evidence by Nurses | Multiple | NA | Audit and feedback  Educational materials  Educational meetings  Educational outreach visits, or academic detailing  Inter professional education  Local consensus processes  Local opinion leaders  Reminders  Tailored interventions | - Compliance of integration of research-based evidence into practice: Adoption |
| **81** | **Zaher 2012 [120]** | Practice-based small group learning programs | Multiple | NA | Educational materials  Educational meetings | - Change in knowledge: Knowledge - Change in skills: Adoption |

KP: knowledge product.
