# Supplementary material for: Implementation Strategies for Knowledge Products in Primary Health Care: Systematic Review of Systematic Reviews
Source: Interact J Med Res. 2022 Jul 11;11(2):e38419. doi: 10.2196/38419 (PMC9315889; doi:10.2196/38419)
Supplement: Multimedia Appendix 6 [file ijmr_v11i2e38419_app6.doc]

**Quality appraisal of the included reviews**

| **№** | **Author and Year** | **Title** | **AMSTAR-2 STANDARDS** | | | | | | | | | | | | | | | | **Overall Score** |
| --- | --- | --- | --- | --- | --- | --- | --- | --- | --- | --- | --- | --- | --- | --- | --- | --- | --- | --- | --- |
|  |  |  | **1** | **2** | **3** | **4** | **5** | **6** | **7** | **8** | **9** | **10** | **11** | **12** | **13** | **14** | **15** | **16** |  |
| 1 | Abdullah 2014 [40] | Measuring the Effectiveness of Mentoring as a Knowledge Translation Intervention for Implementing Empirical Evidence: A Systematic Review | Y | PY | N | Y | Y | Y | Y | Y | Y | N | NMAC | NMAC | Y | Y | NMAC | Y | Moderate |
| 2 | Al Zoubi 2018 [41] | The effectiveness of interventions designed to increase the uptake of clinical practice guidelines and best practices among musculoskeletal professionals: a systematic review | Y | N | Y | Y | Y | Y | Y | Y | Y | N | NMAC | NMAC | Y | Y | NMAC | Y | Low |
| 3 | Albrecht 2016 [42] | Systematic Review of Knowledge Translation Strategies to Promote Research Uptake in Child Health Settings | Y | N | Y | PY | Y | Y | N | PY | N | N | NMAC | NMAC | Y | Y | NMAC | Y | Critically low |
| 4 | Bacci 2019 [43] | Community pharmacist patient care services: A systematic review of approaches used for implementation and evaluation | Y | Y | Y | PY | Y | Y | N | N | N | N | NMAC | NMAC | N | N | NMAC | Y | Critically low |
| 5 | Baker 2015 [44] | Tailored interventions to address determinants of practice (Review) | Y | Y | Y | Y | Y | Y | Y | Y | Y | N | Y | Y | Y | Y | Y | Y | High |
| 6 | Barwick 2012 [45] | Knowledge translation efforts in child and youth mental health: a systematic review | Y | N | N | N | Y | N | N | Y | Y | N | NMAC | NMAC | N | Y | NMAC | Y | Critically low |
| 7 | Boersma 2015 [46] | The art of successful implementation of psychosocial interventions in residential dementia care: a systematic review of the literature based on the RE-AIM framework | Y | N | N | PY | Y | Y | N | Y | N | N | NMAC | NMAC | N | Y | NMAC | Y | Critically low |
| 8 | Bright 2012 [47] | Effect of Clinical Decision-Support Systems A Systematic Review | Y | PY | Y | N | Y | N | N | N | N | N | N | N | N | Y | N | Y | Critically low |
| 9 | Brusamento 2012 [48] | Assessing the effectiveness of strategies to implement clinical guidelines for the management of chronic diseases at primary care level in EU Member States: A systematic review | Y | N | N | PY | Y | Y | Y | Y | Y | N | NMAC | NMAC | Y | Y | NMAC | Y | Low |
| 10 | Bywood 2009 [49] | Effectiveness of opinion leaders for getting research into practice in the alcohol and other drugs field: Results from a systematic literature review | Y | N | Y | PY | N | Y | N | PY | Y | N | NMAC | NMAC | Y | Y | NMAC | N | Critically low |
| 11 | Campbell 2019 [50] | Knowledge Translation Strategies Used by Healthcare Professionals in Child Health Settings: An Updated Systematic Review | Y | N | Y | N | Y | Y | N | PY | Y | N | NMAC | NMAC | Y | Y | NMAC | Y | Critically low |
| 12 | Chaillet 2006 [51] | Evidence-based strategies for implementing guidelines in obstetrics - A systematic review | Y | N | Y | Y | N | Y | N | N | Y | N | NMAC | NMAC | N | N | NMAC | Y | Critically low |
| 13 | Ciliska 2005 [52] | Diffusion and dissemination of evidence-based dietary strategies for the prevention of cancer | N | N | N | Y | N | Y | N | Y | Y | Y | NMAC | NMAC | Y | Y | NMAC | Y | Critically low |
| 14 | Colquhoun 2017 [53] | A systematic review of interventions to increase the use of standardized outcome measures by rehabilitation professionals | N | N | N | PY | Y | Y | N | Y | Y | N | NMAC | NMAC | Y | N | NMAC | Y | Critically low |
| 15 | Davis 1997 [54] | Translating guidelines into practice. A systematic review of theoretic concepts, practical experience and research evidence in the adoption of clinical practice guidelines | Y | N | N | PY | N | N | N | N | N | N | NMAC | NMAC | N | N | NMAC | N | Critically low |
| 16 | De Angelis 2016 [55] | Information and Communication Technologies for the Dissemination of Clinical Practice Guidelines to Health Professionals: A Systematic Review | Y | N | N | Y | Y | N | Y | Y | Y | N | NMAC | NMAC | Y | Y | NMAC | Y | Low |
| 17 | Dexheimer 2008 [56] | Prompting clinicians about preventive care measures: a systematic review of randomized controlled trials | Y | Y | N | PY | Y | Y | N | PY | Y | N | Y | Y | Y | N | N | Y | Critically low |
| 18 | Dexheimer 2014 [57] | A systematic review of the implementation and impact of asthma protocols | Y | N | N | PY | Y | N | N | N | N | N | NMAC | NMAC | Y | Y | NMAC | Y | Critically low |
| 19 | Dwamena 2012 [58] | Interventions for providers to promote a patient-centred approach in clinical consultations | Y | Y | Y | Y | Y | Y | Y | Y | Y | N | Y | Y | Y | Y | Y | Y | High |
| 20 | Espallargues 2000 [59] | Provision of feedback on perceived health status to health care professionals - A systematic review of its impact | Y | N | N | N | Y | N | N | PY | Y | N | Y | N | N | Y | Y | Y | Critically low |
| 21 | Flodgren 2013 [60] | Interventions to improve professional adherence to guidelines for prevention of device-related infections | Y | Y | Y | Y | Y | Y | Y | Y | Y | N | NMAC | NMAC | Y | Y | NMAC | Y | High |
| 22 | Flodgren 2016 [61] | Tools developed and disseminated by guideline producers to promote the uptake of their guidelines (Review) | Y | Y | Y | Y | Y | Y | Y | Y | Y | Y | NMAC | NMAC | Y | Y | NMAC | Y | High |
| 23 | Flodgren 2017 [62] | Interventions to change the behaviour of health professionals and the organisation of care to promote weight reduction in children and adults with overweight or obesity | Y | PY | Y | PY | Y | Y | Y | Y | Y | Y | Y | N | Y | Y | Y | Y | High |
| 24 | Flodgren 2019 [63] | Local opinion leaders: effects on professional practice and healthcare outcomes (Review) | Y | PY | Y | Y | Y | Y | Y | Y | Y | Y | NMAC | NMAC | Y | Y | NMAC | Y | High |
| 25 | Forman-Hoffman 2017 [64] | Quality improvement, implementation, and dissemination strategies to improve mental health care for children and adolescents: a systematic review | Y | Y | Y | Y | Y | Y | Y | PY | Y | N | NMAC | NMAC | Y | Y | NMAC | Y | High |
| 26 | Gagnon 2009 [65] | Interventions for promoting information and communication technologies adoption in healthcare professionals (Review) | Y | Y | N | PY | Y | Y | Y | Y | Y | N | NMAC | NMAC | Y | Y | NMAC | Y | High |
| 27 | Gifford 2007 [66] | Managerial Leadership for Nurses’ Use of Research Evidence: An Integrative Review of the Literature | Y | N | Y | N | Y | N | N | Y | Y | N | NMAC | NMAC | Y | Y | NMAC | N | Critically low |
| 28 | Gould 2017 [67] | Interventions to improve hand hygiene compliance in patient care | Y | Y | Y | Y | Y | Y | Y | Y | Y | Y | NMAC | NMAC | Y | Y | NMAC | Y | High |
| 29 | Goveia 2013 [68] | Educational interventions to improve the meaningful use of Electronic Health Records: A review of the literature: BEME Guide No. 2 | Y | N | N | PY | Y | Y | N | Y | PY | N | NMAC | NMAC | N | N | NMAC | Y | Critically low |
| 30 | Gross 2001 [69] | Implementing Practice Guidelines for Appropriate Antimicrobial Usage: A Systematic Review | Y | N | N | N | N | N | N | PY | N | N | NMAC | NMAC | Y | N | NMAC | N | Critically low |
| 31 | Haggman-Laitila 2016 [70] | A systematic review of the outcomes of educational interventions relevant to nurses with simultaneous strategies for guideline implementation | Y | N | Y | PY | Y | N | N | Y | PY | N | NMAC | NMAC | Y | Y | NMAC | Y | Critically low |
| 32 | Hamade 2019 [71] | Interventions to improve the use of EMRs in primary health care: a systematic review and meta-analysis | Y | N | N | PY | Y | N | N | Y | Y | N | N | N | Y | Y | Y | Y | Critically low |
| 33 | Heselmans 2009 [72] | Effectiveness of electronic guideline-based implementation systems in ambulatory care settings - a systematic review | Y | N | N | PY | Y | Y | Y | PY | Y | N | NMAC | NMAC | Y | Y | NMAC | Y | Low |
| 34 | Hoomans 2007 [73] | The Methodological Quality of Economic Evaluations of Guideline Implementation into Clinical Practice: A Systematic Review of Empiric Studies | Y | N | N | PY | Y | Y | N | N | Y | N | NMAC | NMAC | Y | N | NMAC | Y | Critically low |
| 35 | Imamura 2017 [74] | A systematic review of implementation strategies to deliver guidelines on obstetric care practice in low-and middle-income countries | N | PY | Y | PY | Y | N | N | Y | Y | N | NMAC | NMAC | Y | N | NMAC | Y | Low |
| 36 | Ince 2016 [75] | A systematic review of the implementation of recommended psychological interventions for schizophrenia: Rates, barriers, and improvement strategies | Y | N | Y | PY | N | N | N | Y | Y | N | NMAC | NMAC | N | N | NMAC | N | Critically low |
| 37 | Ista 2013 [76] | Do implementation strategies increase adherence to pain assessment in hospitals? A systematic review | Y | N | Y | PY | Y | Y | N | Y | Y | N | NMAC | NMAC | N | Y | NMAC | Y | Critically low |
| 38 | Jeffery 2015 [77] | Interventions to improve adherence to cardiovascular disease guidelines: a systematic review | Y | PY | N | PY | Y | Y | N | Y | Y | N | Y | Y | Y | Y | Y | Y | Low |
| 39 | Jensen 2016 [78] | Systematic review of the cost-effectiveness of implementing guidelines on low back pain management in primary care: is transferability to other countries possible? | Y | N | N | PY | Y | N | N | Y | Y | Y | NMAC | NMAC | Y | N | NMAC | Y | Critically low |
| 40 | Jones 2014 [79] | Effectiveness of interventions to increase hepatitis C testing uptake among high-risk groups: a systematic review | Y | PY | N | Y | Y | N | N | N | Y | N | NMAC | NMAC | Y | N | NMAC | Y | Low |
| 41 | Jones 2015 [80] | Translating Knowledge in Rehabilitation: Systematic Review | Y | Y | Y | PY | Y | N | N | Y | Y | N | NMAC | NMAC | Y | Y | NMAC | N | Low |
| 42 | Kovacs 2018 [81] | Systematic Review and Meta-analysis of the Effectiveness of Implementation Strategies for Non-communicable Disease Guidelines in Primary Health Care | N | N | N | PY | N | N | N | Y | Y | N | N | N | Y | Y | Y | Y | Critically low |
| 43 | Légaré 2012 [82] | Patients’ Perceptions of Sharing in Decisions A Systematic Review of Interventions to Enhance Shared Decision Making in Routine Clinical Practice | Y | N | N | PY | Y | Y | N | Y | Y | N | NMAC | NMAC | Y | Y | NMAC | Y | Critically low |
| 44 | Lineker 2010 [83] | Educational Interventions for Implementation of Arthritis Clinical Practice Guidelines in Primary Care: Effects on Health Professional Behavior | Y | N | N | PY | N | N | N | PY | N | N | NMAC | NMAC | N | N | NMAC | N | Critically low |
| 45 | Luangasanatip 2015 [84] | Comparative efficacy of interventions to promote hand hygiene in hospital: systematic review and network meta-analysis | N | Y | N | PY | N | N | Y | Y | Y | N | Y | N | Y | Y | Y | Y | Moderate |
| 46 | Medves 2010 [85] | Systematic review of practice guideline dissemination and implementation strategies for healthcare teams and team-based practice | Y | PY | Y | N | Y | Y | N | N | Y | N | NMAC | NMAC | N | N | NMAC | Y | Critically low |
| 47 | Menon 2009 [86] | Strategies for rehabilitation professionals to move evidence-based knowledge into practice: a systematic review | Y | N | N | PY | N | N | N | Y | Y | N | NMAC | NMAC | Y | Y | NMAC | Y | Critically low |
| 48 | Murthy 2012 [87] | Interventions to improve the use of systematic reviews in decision-making by health system managers, policy makers and clinicians (Review) | Y | PY | N | Y | Y | Y | Y | Y | Y | N | NMAC | NMAC | Y | Y | NMAC | Y | Moderate |
| 49 | Nilsen 2006 [88] | Effectiveness of strategies to implement brief alcohol intervention in primary healthcare A systematic review | Y | N | Y | PY | N | N | N | Y | N | N | NMAC | NMAC | Y | Y | NMAC | N | Critically low |
| 50 | Noonan 2014 [89] | Knowledge translation and implementation in spinal cord injury: a systematic review | Y | PY | N | PY | Y | Y | N | Y | Y | N | NMAC | NMAC | Y | N | NMAC | Y | Low |
| 51 | Novins 2013 [90] | Dissemination and implementation of evidence-based practices for child and adolescent mental health: a systematic review | Y | N | N | PY | Y | N | N | Y | PY | Y | NMAC | NMAC | N | N | NMAC | Y | Critically low |
| 52 | Okelo 2013 [91] | Interventions to modify health care provider adherence to asthma guidelines: a systematic review | Y | Y | N | PY | Y | Y | N | Y | Y | N | NMAC | NMAC | N | Y | NMAC | Y | Critically low |
| 53 | Ospina 2013 [92] | A systematic review of the effectiveness of knowledge translation interventions for chronic noncancer pain management | Y | Y | Y | PY | Y | N | Y | Y | Y | N | NMAC | NMAC | Y | Y | NMAC | Y | Moderate |
| 54 | Pearson 2009 [93] | Do computerised clinical decision support systems for prescribing change practice? A systematic review of the literature (1990-2007) | Y | N | Y | PY | N | Y | Y | Y | Y | N | NMAC | NMAC | Y | Y | NMAC | Y | Low |
| 55 | Perrier 2011 [94] | Interventions Encouraging the Use of Systematic Reviews in Clinical Decision-Making: A Systematic Review | Y | N | N | Y | N | Y | N | Y | Y | N | NMAC | NMAC | Y | Y | NMAC | Y | Moderate |
| 56 | Perry 2011 [95] | Effects of educational interventions on primary dementia care: A systematic review | Y | N | N | PY | Y | Y | N | Y | Y | N | NMAC | NMAC | N | Y | NMAC | Y | Critically low |
| 57 | Pham 2019 [96] | Strategies for implementing shared decision making in elective surgery by health care practitioners: A systematic review | Y | Y | N | Y | Y | N | N | Y | Y | N | NMAC | NMAC | N | Y | NMAC | Y | Critically low |
| 58 | Powell 2014 [97] | A Systematic Review of Strategies for Implementing Empirically Supported Mental Health Interventions | Y | N | Y | PY | N | Y | N | Y | N | N | NMAC | NMAC | Y | N | NMAC | Y | Critically low |
| 59 | Rosen 2016 [98] | A Review of Studies on the System-Wide Implementation of Evidence-Based Psychotherapies for Posttraumatic Stress Disorder in the Veterans Health Administration | Y | PY | N | Y | N | Y | N | Y | N | N | NMAC | NMAC | N | N | NMAC | Y | Critically low |
| 60 | Scott 2012 [99] | Systematic review of knowledge translation strategies in the allied health professions | Y | Y | Y | Y | Y | Y | N | Y | Y | N | NMAC | NMAC | Y | Y | NMAC | Y | Low |
| 61 | Shanbhag 2018 [100] | Effectiveness of implementation interventions in improving physician adherence to guideline recommendations in heart failure: a systematic review | Y | Y | Y | PY | Y | Y | N | Y | Y | N | NMAC | NMAC | Y | Y | NMAC | Y | Low |
| 62 | SHIFFMAN 1999 [101] | Computer-based Guideline Implementation Systems: A Systematic Review of Functionality and Effectiveness | Y | N | N | PY | N | Y | N | PY | N | N | NMAC | NMAC | N | Y | NMAC | Y | Critically low |
| 63 | Siddiqui 2011 [102] | The role of physician reminders in faecal occult blood testing for colorectal cancer screening | Y | N | N | PY | Y | Y | Y | Y | Y | N | Y | N | Y | Y | N | Y | Critically low |
| 64 | Smeets 2007 [103] | Effectiveness and costs of implementation strategies to reduce acid suppressive drug prescriptions: a systematic review | Y | N | Y | PY | Y | N | N | PY | N | N | NMAC | NMAC | Y | Y | NMAC | Y | Critically low |
| 65 | Smolders 2008 [104] | Knowledge Transfer and Improvement of Primary and Ambulatory Care for Patients With Anxiety | N | N | Y | N | Y | Y | N | Y | Y | N | Y | N | Y | Y | N | N | Critically low |
| 66 | Soumerai 1989 [105] | Improving drug prescribing in primary care - a critical analysis of the experimental literature | N | N | Y | N | N | N | N | PY | N | N | NMAC | NMAC | N | N | NMAC | N | Critically low |
| 67 | Souza 2011 [106] | Computerized clinical decision support systems for primary preventive care: A decision-maker-researcher partnership systematic review of effects on process of care and patient outcomes | Y | Y | Y | PY | Y | Y | N | PY | Y | Y | NMAC | NMAC | Y | Y | NMAC | Y | Low |
| 68 | Sunderji 2018 [107] | Advancing Integrated Care through Psychiatric Workforce Development: A Systematic Review of Educational Interventions to Train Psychiatrists in Integrated Care | Y | Y | N | PY | Y | Y | N | N | Y | N | NMAC | NMAC | Y | N | NMAC | Y | Low |
| 69 | Thomas 1999b [108] | Guidelines in professions allied to medicine (Review) | Y | PY | N | Y | Y | Y | Y | Y | Y | N | NMAC | NMAC | Y | N | NMAC | Y | Moderate |
| 70 | Thompson 2007 [109] | Interventions aimed at increasing research use in nursing: a systematic review | Y | N | N | Y | Y | Y | Y | Y | Y | N | NMAC | NMAC | Y | Y | NMAC | Y | Low |
| 71 | Tudor Car 2019 [110] | Health professions digital education on clinical practice guidelines: a systematic review by Digital Health Education collaboration | Y | N | N | Y | Y | Y | N | PY | Y | N | Y | N | Y | Y | N | Y | Critically low |
| 72 | Unverzagt 2014 [111] | Strategies for guideline implementation in primary care focusing on patients with cardiovascular disease: a systematic review | Y | Y | N | PY | Y | N | N | Y | Y | N | Y | Y | Y | Y | Y | Y | Low |
| 73 | van Steenkiste 2008 [112] | Systematic review of implementation strategies for risk tables in the prevention of cardiovascular diseases | Y | N | Y | PY | Y | N | N | Y | Y | N | NMAC | NMAC | N | N | NMAC | Y | Critically low |
| 74 | Watkins 2015 [113] | Effectiveness of implementation strategies for clinical guidelines to community pharmacy: a systematic review | Y | Y | Y | PY | Y | Y | N | Y | Y | N | NMAC | NMAC | Y | N | NMAC | Y | Low |
| 75 | Wees 2008 [114] | Multifaceted strategies may increase implementation of physiotherapy clinical guidelines: a systematic review | Y | N | N | PY | N | N | Y | Y | Y | N | NMAC | NMAC | N | Y | NMAC | N | Critically low |
| 76 | Weinman 2007 [115] | Effects of implementation of psychiatric guidelines on provider performance and patient outcome: systematic review | N | N | Y | Y | Y | N | N | Y | Y | N | NMAC | NMAC | Y | Y | NMAC | Y | Critically low |
| 77 | Wensing 1998 [116] | Implementing guidelines and innovations in general practice: which interventions are effective? | Y | N | N | N | Y | N | N | N | N | N | NMAC | NMAC | N | N | NMAC | N | Critically low |
| 78 | Wilbur 2018 [117] | Systematic Review of Standardized Patient Use in Continuing Medical Education | Y | N | N | Y | Y | N | N | Y | Y | N | NMAC | NMAC | N | N | NMAC | Y | Critically low |
| 79 | Wilson 2016 [118] | Knowledge translation studies in paediatric emergency medicine: A systematic review of the literature | Y | N | Y | PY | Y | N | Y | Y | N | N | NMAC | NMAC | Y | N | NMAC | Y | Critically low |
| 80 | Wuchner 2014 [119] | Integrative Review of Implementation Strategies for Translation of Research-Based Evidence by Nurses | Y | N | N | PY | N | N | N | PY | N | N | NMAC | NMAC | N | Y | NMAC | Y | Critically low |
| 81 | Zaher 2012 [120] | Practice-based small group learning programs | N | N | N | N | N | N | N | PY | N | N | NMAC | NMAC | N | N | NMAC | Y | Critically low |

Y=Yes. PY=Partial Yes. N=No. NMAC=No meta-analysis conducted

AMSTAR-2 STANDARDS

1. Did the research questions and inclusion criteria for the review include the components of PICO?

2. Did the report of the review contain an explicit statement that the review methods were established prior to the conduct of the review and did the report justify any significant deviations from the protocol?

3. Did the review authors explain their selection of the study designs for inclusion in the review?

4. Did the review authors use a comprehensive literature search strategy?

5. Did the review authors perform study selection in duplicate?

6. Did the review authors perform data extraction in duplicate?

7. Did the review authors provide a list of excluded studies and justify the exclusions?

8. Did the review authors describe the included studies in adequate detail?

9. Did the review authors use a satisfactory technique for assessing the risk of bias (RoB) in individual studies that were included in the review?

10. Did the review authors report on the sources of funding for the studies included in the review?

11. If meta-analysis was performed did the review authors use appropriate methods for statistical combination of results?

12. If meta-analysis was performed, did the review authors assess the potential impact of RoB in individual studies on the results of the meta-analysis or other evidence synthesis?

13. Did the review authors account for RoB in individual studies when interpreting/discussing the results of the review?

14. Did the review authors provide a satisfactory explanation for, and discussion of, any heterogeneity observed in the results of the review?

15. If they performed quantitative synthesis did the review authors carry out an adequate investigation of publication bias (small study bias) and discuss its likely impact on the results of the review?

16. Did the review authors report any potential sources of conflict of interest, including any funding they received for conducting the review?
